# Experimental and Theoretical Insights into the Effect of Dioldibenzoate Isomers on the Performance of Polypropylene Catalysts

**DOI:** 10.3390/polym16040559

**Published:** 2024-02-19

**Authors:** Huasheng Feng, Changxiu Li, Junling Zhou, Xiaofan Zhang, Shuxuan Tang, Xiangya Xu, Zhihui Song

**Affiliations:** 1 Division of Catalytic Science, SINOPEC (Beijing) Research Institute of Chemical Industry Co., Ltd., Beijing 100013, China; xuxy.bjhy@sinopec.com; 2 Division of Polypropylene Research, SINOPEC (Beijing) Research Institute of Chemical Industry Co., Ltd., Beijing 100013, China; zhoujl.bjhy@sinopec.com (J.Z.); zhangxf.bjhy@sinopec.com (X.Z.); tangshx.bjhy@sinopec.com (S.T.); 3 Division of Polyethylene Research, SINOPEC (Beijing) Research Institute of Chemical Industry Co., Ltd., Beijing 100013, China; songzhh.bjhy@sinopec.com

**Keywords:** Ziegler-Natta catalyst, DFT calculation, electron donor, polypropylene, polymerization

## Abstract

Experimental investigations and density functional theory (DFT) calculations were carried out to study the comprehensive effect of different 3,5-heptanedioldibenzoate (HDDB) optical isomers as the internal electron donor on the catalytic performance of Ziegler−Natta catalysts. The experimental catalytic activity of HDDB has a positive correlation with the relative content of the mesomer incorporated during catalyst preparation, while the hydrogen response of HDDB displayed a negative correlation with the relative content of the mesomer. In order to apply the DFT calculation results to the macroscopic activity of the catalyst, the content of the active centers of the catalyst was analyzed. Assuming that the content of the active centers is proportional to the internal electron donor content of the catalyst, binary linear regression was carried out, which showed a good linear correlation between experimental activity data and internal electron donor content. Furthermore, the fitted activity of the single active centers aligned well with the calculated activation energies. These results revealed that the catalytic activity of polypropylene (PP) catalysts is dependent on both the active center content and the catalytic activity of an individual active center. Additionally, the lower hydrogen response of HDDB leads to a higher molecular weight of polypropylene obtained from the *RS*-containing catalyst compared to the *SS*-containing catalyst. Further study reveals that the hydrogen transfer reactions of 2,4-pentanediol dibenzoate (PDDB)/HDDB are influenced by the orientation of the methyl/ethyl groups in different isomers, which affect the activation energy differences between the hydrogen transfer reaction and the propylene insertion reaction, and finally influence the molecular weight of PP.

## 1. Introduction

Ziegler-Natta (ZN) catalysts have achieved great success in the polyolefin industry since their invention [1,2], with an annual production exceeding 150 million tons [3]. Notably, the production of polypropylene (PP) reached 102.8 million tons in 2021, and more than 90% of polypropylene was produced using ZN catalysts. The ZN catalysts consist of TiCl_4_, MgCl_2_ support, and internal electron donors (IDs), while external electron donors (EDs) and initiators are added during polymerization [4]. Until now, industrial ZN catalysts have been developed to the fifth generation. Starting from the third generation of ZN catalysts, catalytic performance has been improved predominantly through the development of IDs [5]. The IDs for the fifth generation of industrial ZN catalysts are non-phthalate compounds including 1,3-diethers [6,7], succinates [8,9], and diol diesters [10,11,12]. In addition, new electron donors such as sulfonyl amine [13,14,15] and its derived diesters [16] are also expected to be introduced into industrialization.

Despite the remarkable success and advancements of ZN catalysts in the industry, academic research on the mechanism of ZN catalysts is complicated, due to its multi-component disperse structures and the challenges associated with the microstructure characterizations. Recently, several studies involving the exploration of microscopic active centers have been carried out [17,18]. Fan et al. explored the catalyst active center numbers during polymerization reactions using the quenching method [19,20]. Christophe and co-workers investigated the interactions between the support and the electron donor [21]. Unfortunately, experiments on characterizing the precise structures of active centers are generally expensive and difficult. Since the 21st century, a large number of theoretical studies have been applied to the mechanism research of ZN catalysts [22], including explorations on the interactions among each component [23,24,25,26,27,28,29,30,31,32,33,34,35] and the influences of electron donors on the catalytic performance of ZN catalysts [36,37,38,39,40,41,42]. Although earlier computational studies had inconsistent conclusions with the experiment results due to different models and computational methods [43], the polymerization process of propylene in the MgCl_2_/TiCl_4_/ID system has been gradually elucidated in recent years. During this process, TiCl_4_ is adsorbed on a (110)-facet of MgCl_2_ to form a stable active center, and the electron donor close to TiCl_4_ enhances the stereoselectivity of the ZN catalyst. Some studies also revealed that triethylaluminium could affect the stereoselectivity of ZN catalysts [44,45,46].

Compared with the current qualitative research on ZN catalysts that explains the basic mechanism of ZN catalyst systems [47], there is a lack of quantitative simulation for industrial ZN catalysts. Unlike studies on isotacticity, which are easily explored through the comparison of different active energies for a single active center, research on macroscopic properties such as catalytic activity and average molecular weight is rare. Macroscopic property research must consider factors such as the quantity of active centers, various reaction conditions, and changes in the actual reaction environment. Moreover, significant differences in the actual reaction performance would appear when using different preparation methods for the same catalyst composition, even when using the same type of electron donor isomers [43].

Catalysts [11,12,48] containing different isomers of diol diester electron donors have significantly different properties. In our previous study [49], we reported that the ZN catalyst using optical isomers of electron donor PDDB (2,4-pentanedioldibenzoate) has significant differences in its ID content and stereoselectivity. DFT calculations showed that the primary factors contributing to the different content of ID isomers in the catalyst are the distinct conformations observed in the free state and adsorption state of ID molecules, along with different adsorption energies on the MgCl_2_ support.

In this manuscript, we extend the exploration of diol diester IDs from PDDB to HDDB. Apart from the investigations on stereoselectivity, comprehensive theoretical calculations were carried out to elucidate the molecular level understanding of propylene polymerization catalyzed by ZN catalysts, and to investigate the factors contributing to diverse performances including catalytic activity, molecular weight, and other properties.

## 2. Experiment and Calculation Sections

### 2.1. Experiments

#### 2.1.1. Materials

All operations were executed under a nitrogen atmosphere using glove-box and standard Schlenk (Beijing, China) techniques. Propylene and MgCl_2_ were obtained from Sinopec (Beijing, China); AlEt_3_, TiCl_4_, and cyclohexylmethyldimethoxylalkoxysilane (CHMMS) were purchased from J&K Scientific (Beijing, China) without further purification; AlEt_3_ and CHMMS were diluted in n-hexane to 0.5 mol/L and 0.1 mol/L, respectively. All other solvents were purchased from Sinopharm Chemical Reagent Co. Ltd. (Shanghai, China) and dried with 4Å molecular sieves in advance.

#### 2.1.2. Catalyst Preparation

The stereoisomers of HDDB (characterized using ^1^H NMR, see Figures S1 and S2) and the MgCl_2_-supported catalysts [11,12,48] were synthesized or prepared according to the previously reported procedure. A typical synthesis procedure of MgCl_2_-supported catalysts is as follows: First, 4.8 g magnesium chloride, 95 mL toluene, 4 mL epoxy chloropropane, and 12.5 mL tributyl phosphate were added successively to a nitrogen-purged five-necked reactor. The mixture was heated to 60 °C with stirring and maintained at this temperature for 1.5 h. Then, 1.4 g phthalic anhydride was added to the reaction mixture and the reaction was maintained for 1 h. The solution was then cooled down, and 56 mL TiCl_4_ was introduced slowly below −20 °C. After the mixture was heated to 80 °C, 5 mmol of IDs (HDDB with different contents of mesomer and racemate, or others) was added and maintained for 1 h. After removing the supernatant, the residue was washed with toluene. The obtained solid precipitate was treated twice with 40 mL TiCl_4_ and 60 mL toluene at 100 °C for 2 h. After sedimentation and filtration, the residue was washed with 60 mL hexane to obtain the solid catalyst.

#### 2.1.3. Bulk Polymerization Process

Propylene polymerization was carried out in a 5 L stainless steel reactor with liquefied propylene [11,12,48]. The typical procedure for the bulk polymerization of propylene was as follows: A total of 5 mL AlEt_3_, 1 mL CHMMS, about 9 mg of the solid catalyst, and 1.2 SL (standard liter) hydrogen were added. Then, 2.3 L liquid propylene was added to the 5 L stainless steel reactor, which had been replaced with propylene gas completely. The reactor was heated to 70 °C and the polymerization was processed at 70 °C for 1 h. Then, polypropylene (PP) resin powder was obtained after reducing both the temperature and pressure.

#### 2.1.4. Characterization

Nuclear magnetic resonance (^1^H NMR) was characterized using the Bruke dmx300 nuclear magnetic resonance spectrometer (300 MHz, solvent is CDCl_3_, TMS as internal standard, and measuring temperature is 300 K).

The content of *meso*-/*rac*-HDDB was detected using liquid chromatography (LC). The content of *meso*-/*rac*-HDDB in compounds was detected directly. The catalyst was first dissolved in an acidic media and then extracted using ethyl acetate, to be examined. Furthermore, separations were performed on the Waters-600E UPLC H-Class (Waters Corporation, 34 Maple Street, Milford, MA, USA) with a column of ACQUITY UPLC BEH C18 (50 mm × 2.1 mm, 1.7 μm). The absolute content of total HDDB (including mesomer and racemate) in the catalyst was calculated by using a standard curve.

The isotactic index (I.I) of the polymer was calculated using the following method: First, 2 g dried polymer sample is extracted with boiling heptane in an extractor for 6 h, then the residual substance is dried to a constant weight, and the ratio of the weight (g) of residual polymer to two is named as the isotactic index.

The melt flow rate (MFR) of the polymer was measured using the test standard GB/T 3682.1–2018 [50] (corresponds to the standard of ASTM D 1238-13). The MFR is the amount of the polymer (g) flowing through the capillary for 10 min, under a pressure of 2.16 kg at 230 °C.

The molecular weight and its distribution (PD) of the polypropylene were determined using a Waters Alliance V2000 gel permeation chromatograph (GPC) equipped with a refractive index detector, using three Polymer Laboratory MIXED-B columns and 1,2,4-trichlorobenzene as the solvent at 150 °C. The number-average and weight-average molecular weight (*M*_n_ and *M*_w_, respectively) values were evaluated with reference to a polystyrene standard calibration.

### 2.2. Computational Details

All DFT calculations were performed with the Gaussian16 program, Revision C.01. All geometry optimizations of intermediates and transition states were carried out using PBE0-D3/6-311G(d,p) [51,52] in combination with the Becke–Johnson (BJ) damping correlation [53]. Frequency calculations were also conducted at the same level of theory to obtain vibrational frequencies to determine the identity of stationary points as intermediates (no imaginary frequencies) or transition states (only one imaginary frequency). To avoid the errors associated with low frequency, Grimme’s quasi-RRHO [54] model was used for Gibbs free energy calculations using the Sherm 2.3 program [55]. Compared with the default RRHO model provided by Gaussian16, the quasi-RRHO model demonstrated a quantitative accuracy improvement. Gibbs free energies were calculated at 343.15 K and 1 atm for the stable structures of the catalyst and transition state structures. The MgCl_2_ cluster model, derived from *δ*-MgCl_2_ crystals, underwent geometry optimization with the Mg atoms constrained at a fixed distance of 3.6363 Å (Mg···Mg) while the other atoms are free.

## 3. Results and Discussions

### 3.1. The Polymerization Experiments

The polymerization results of the ZN catalysts with different HDDB stereoisomers as IDs are shown in Table 1 (see Table S1 for the polymerization results of PDDB). With the increase in mesomer content (see Figures S3–S16), the ID content, the activity (AC), and stereoselectivity (characterized using the isotactic index, I.I.) of the catalysts, as well as the molecular weight of the polymer product shows an increasing trend, while the hydrogen response (characterized using the melt flow rate, MFR) and the molecular weight distribution (characterized using the polydispersity, PD) exhibit a decreasing or narrowing trend. These results suggest that the stereoisomer content of HDDB in the catalysts plays a crucial role in the catalytic performance during propylene polymerization.

### 3.2. The Active Center Models for DFT Calculations

**Figure 1:**
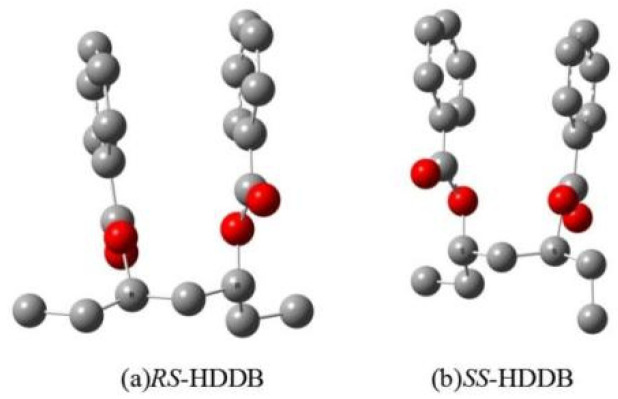
The optimized DFT geometries for the HDDB stereoisomers in the free state (red: oxygen; grey: carbon; hydrogen is omitted).

HDDB has two unequal stereoisomers: racemate and mesomer. In the free state, the free energy of the mesomer is 1.5 kcal/mol higher than that of the racemate isomers. Two oxygen atoms of carbonyl groups in the *RS*-isomer are in the same direction, while those of *SS*-isomers are in the opposite direction (See Figure 1 for the structure of free HDDB).

**Figure 2:**
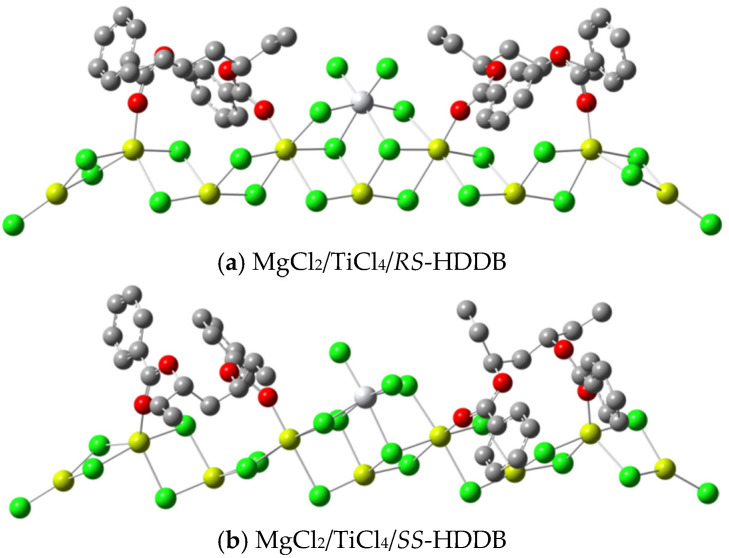
The *trans*-a adsorption modes of HDDB stereoisomers and TiCl_4_ on the (110)-facet of MgCl_2_ (red: oxygen; grey: carbon; green: chlorine; yellow: magnesium; hydrogen is omitted).

Based on our previous work [49], where we compared the adsorption energy of PDDB on MgCl_2_ and the relative energy of various active center models, we assumed that the *trans*-a model (differing only in left/right order as *trans*-a/b) represents the active center structure with the lowest energy for both *RS*-PDDB and *SS*-PDDB. With this model, the structure for the active center with the lowest energy and stereoselectivity were calculated, respectively, revealing that the configuration of donor HDDB resembles that of PDDB (Table 2). Similarly to PDDB, the key difference in the transition state (TS) structures of insertion reactions with the *RS*-HDDB or *SS*-HDDB isomer is the orientation of the ethyl group. For the *RS* isomer, the ethyl group pointed towards the Ti atom, thereby restricting the space near the Ti center, resulting in a large energy difference in the *si*- and *re*-insertion reactions of propylene molecules. Conversely, for the *SS* isomer, the ethyl group is nearly perpendicular to the (110)-facet of MgCl_2_, which has less influence on the active center, resulting in less energy difference for the insertion reaction of propylene. So, in this manuscript, we maintain the use of the *trans*-a model as the active center model for HDDB (See Figure 2 for the active center structure of HDDB).

### 3.3. DFT Calculations about the Catalytic Activity

**Figure 3:**
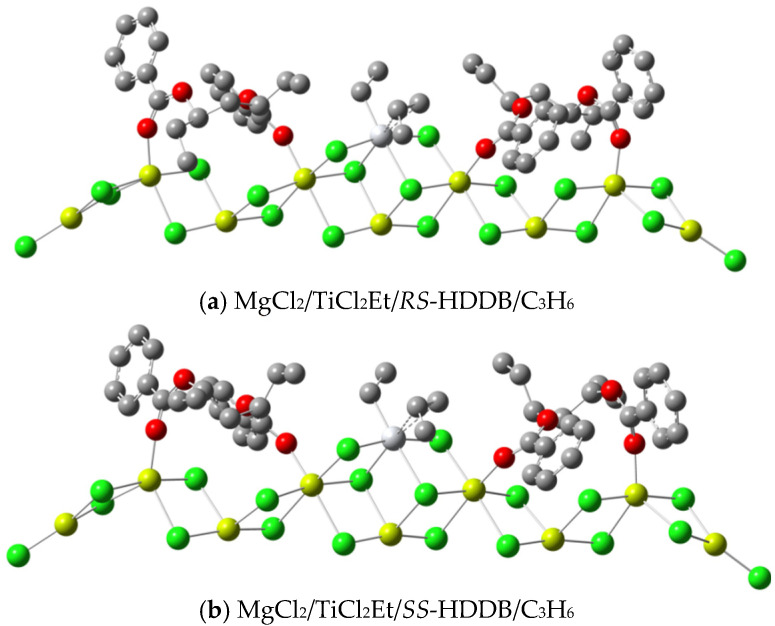
Structures of HDDB–catalyst–propylene complexes (red: oxygen; grey: carbon; green: chlorine; yellow: magnesium; hydrogen is omitted).

**Figure 4:**
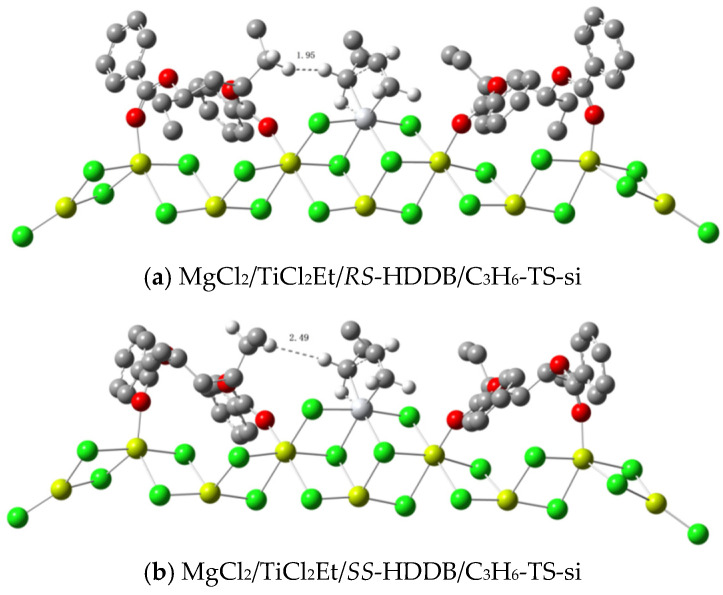
The transition state structures of HDDB–catalyst–propylene (*si*-insertion) complexes (red: oxygen; grey: carbon; green: chlorine; yellow: magnesium; hydrogen is omitted).

Considering that the transition state energy for the *si*-insertion reaction is lower than that of *re*-insertion (see Table 1), the *si*-insertion and propylene–catalyst complex were selected as the reaction channel and energy starting point, respectively to calculate the energy barriers for the active center with a *trans*-a structure. The structures for the HDDB–catalyst complex and HDDB–catalyst–propylene (*si*-insertion) complex are listed in Figure 3 and Figure 4, respectively. According to the calculation results on activation energies, the activity of *SS*-HDDB is slightly higher than that of *RS*-HDDB (see Table 3). The reason for the difference can be seen in Figure 4. The hydrogen atoms of *RS*-HDDB are only 1.95 angstroms apart from the hydrogen atoms of the ethyl group representing the polymer chain. This shows a strong steric effect. The same distance for *SS*-HDDB is 2.49 angstroms, and the interference with the reaction center is almost negligible.

### 3.4. Analysis of Factors Affecting Catalytic Activity

According to the experimental data from Table 1 and Table S1, it is evident that the catalytic activity of the catalyst increases with a higher *RS* isomer content, while the overall IDs would also be increased. We suggest that the adsorption modes for different ID molecules on the MgCl_2_ support are similar during the catalyst preparation processes. Assuming an ideal single-layer adsorption model, where the number of active centers is proportional to the quantity of ID molecules, the model for catalytic activity can be described as

*A*_Tmacro_ = *A_RS_*_macro_ + *A_SS_*_macro_ = *A_RS_C_RS_* + *A_SS_C_SS_* (1)

Here, *A*_Tmacro_ represents the total activity of the catalyst, *A_RS_*_macro_ is the macroscopic activity of the active centers with the *RS* isomer, and *A_SS_*_macro_ is the macroscopic activity of the active centers with the *SS* isomer. *A_RS_* is proportional to the activity of the single *RS* isomer active center, *C_RS_* is the relative content of the *RS* isomer in the catalyst, *A_SS_* is proportional to the activity of the single *SS* isomer active center, and *C_SS_* is the relative content of the *SS* isomer in the catalyst. Additionally, we defined the relative activity ratio of the single *SS* isomer active center to that of the *RS* isomer as *A_SS_*/*A_RS_*.

**Figure 5:**
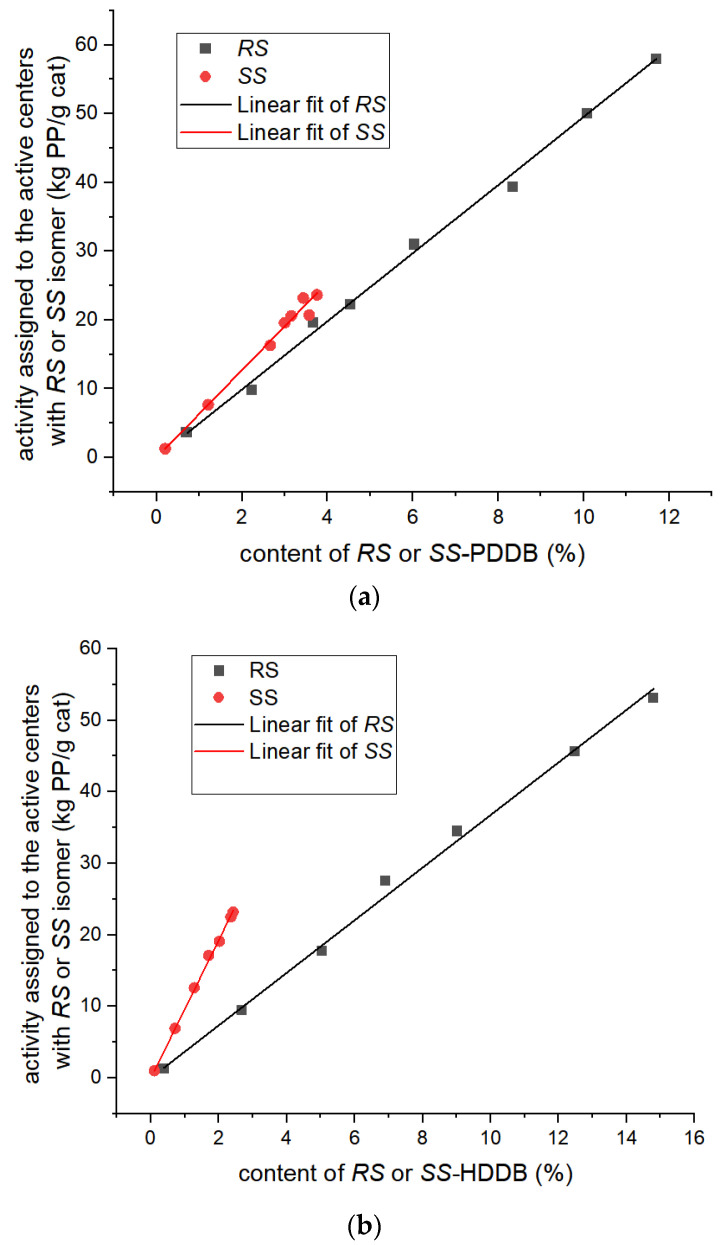
The changes of macroscopic activity for the active centers with (**a**) PDDB and (**b**) HDDB ID when increasing the stereoisomer content. The residuals are distributed according to the relative content percent of the *RS* or *SS* stereoisomers. The slope represents the catalytic activity for the active centers per unit.

The macroscopic activity data and ID isomer content, as presented in Table 1 and Table S1, underwent binary regression analysis and the findings are summarized in Table 4. The outcomes in Table 4 indicate that the catalytic activity for the active center with the *SS* isomer is slightly higher than that of the *RS* isomer for both PDDB and HDDB, which is consistent with the calculated results. Figure 5 shows the changes in macroscopic activity attributed to the active centers when the isomer content increased for both the *RS* and *SS* isomers. Notably, the depicted relationship demonstrates a high degree of linearity, with a regression coefficient exceeding 0.999.

The calculation results indicated that the activity for the active center with the *RS* isomer is lower than that of the *SS* isomer, while the macroscopic activity for the catalyst exhibits a positive correlation with the *RS* content in the experiments. These results revealed that the mesomer of dioldibenzoate improves the experimental catalytic activity of polypropylene (PP) catalysts through increasing the active center content, rather than the catalytic activity of individual active centers. This phenomenon can be attributed to the fact that it is easier for the *RS* isomer to be adsorbed on MgCl_2_ surfaces (see Table 5), resulting in an increase in the content of active centers. This conclusion is also consistent with our previous work [49], where the mesomer ID molecule is easier to be adsorbed on the (110)-facet of the MgCl_2_ support with TiCl_4_, compared with the racemate isomer. For the ratio of TiCl_4_/ID and the catalyst preparation, the procedure remains unchanged in the series of experiments (Entry 1 to Entry 7 in Table 1), and the active center content is reasonably positively proportional to the ID content. The result for the binary regression analysis in Table 4 and Figure 5 is consistent with the calculated relative activity for the active centers with the *SS*/*RS* isomer in Table 3, which reveals that the assumption above is suitable for our catalyst system. These results revealed that the catalytic activity of polypropylene (PP) catalysts is dependent on both the active center content and the catalytic activity of the individual active center.

### 3.5. Hydrogen Transfer Reaction

The chain transfer reaction is quite important in the polymerization process, and the relative rate of the hydrogen transfer reaction to the polymerization affects the molecular weight of polypropylene. Previous studies suggest that the chain transfer to the monomer serves as the primary chain transfer reaction, which could be applied in heterogeneous ZN catalysts [56,57], similar to metallocene [58,59] and non-metallocene systems [60,61]. Under conditions where the monomer concentration is low or the alkylaluminium concentration is relatively high, the chain transfer to the metal and the chain transfer to the cocatalyst may become the dominant chain transfer reactions [62]. In the area of industrial ZN catalysts, the use of hydrogen to control molecular weight makes hydrogenolysis (i.e., the hydrogen transfer reaction) the primary chain transfer reaction [56,63]. The hydrogen transfer reaction can be represented by Equation (2):

Ti–CH_2_–Polymer + H_2_ → Ti–H + CH_3_–Polymer (2)

Considering the existence of H_2_, neglecting other chain transfer reactions (chain transfer to monomer, chain transfer to alkylaluminum, and ethyl *β*-dehydrogenation) and assuming constant concentrations of each reactant, the relationship between the hydrogen transfer reaction and the molecular weight can be expressed as follows:

*M*_w_ α *k*_p_*C*_p_/(*k*_H_*C*_H_) α exp[(∆*G*_H_ − ∆*G*_p_)/*RT*] (3)

where *M*_w_ is molecular weight, *k*_p_ is the rate of propylene insertion reaction, *C*_p_ is the concentration of propylene, *k*_H_ is the rate of hydrogen transfer reaction, *C*_H_ is the concentration of hydrogen, and ∆*G*_H_ and ∆*G*_p_ are the activation energies of the hydrogen transfer reaction and the propylene insertion reaction, respectively.

**Figure 6:**
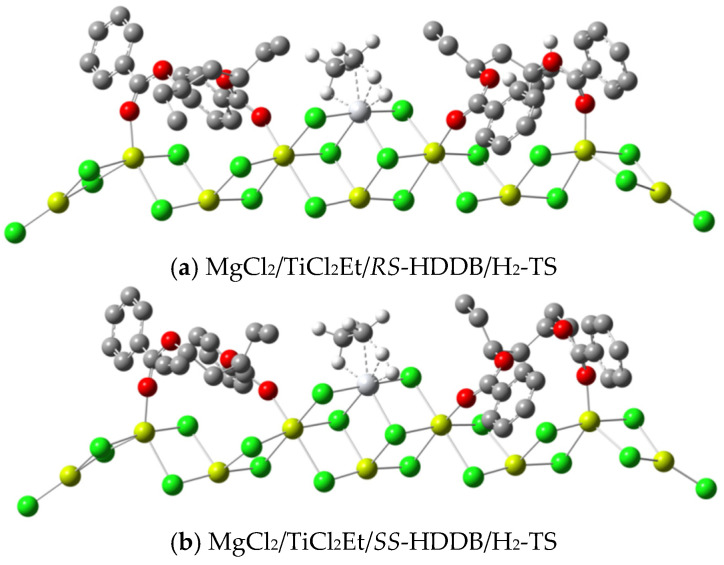
The transition state structures of HDDB stereoisomer catalysts in the hydrogen transfer reaction (red: oxygen; grey: carbon; green: chlorine; yellow: magnesium; hydrogen is omitted).

The hydrogen transfer reaction and propylene insertion reaction compete for the same active center. The difference between their activation energies is positively correlated to the molecular weight of polypropylene. Substituting the propylene molecule with an H_2_ molecule in the model of Figure 4 allowed for the calculation of the hydrogen transfer reaction. The results from the Gibbs energy calculations revealed that there is no stable intermediate complex for H_2_ and the active center. Instead, the hydrogen transfer reaction proceeded directly from a separated state to a transition state. In contrast to Ref. [33], the transfer of polymer chains (represented by the ethyl group in Figure 6) with H_2_ was attributed to the interaction between the *β*-H and Ti center, while the insertion reaction of propylene was attributed to the interaction between the *α*-H and Ti center (see Figure 6 for the transition state structures). The activation energies are listed in Table 6, which suggests that the transfer activation energy of H_2_ at the *RS* active center is higher than that of the *SS* active center for both PDDB and HDDB. This result indicates that the hydrogen transfer reaction in the *RS* isomer system is slightly more challenging than in the *SS* isomer system, consistent with the experimental results where the molecular weight of polypropylene obtained from the *RS*-containing catalyst is higher than that from the *SS*-containing catalyst.

The differences in catalytic activity and activation energy showed that the orientation for the methyl/ethyl groups of *RS* isomers led to the variations in hydrogen transfer reactions, which finally resulted in the distinct hydrogen response and polypropylene molecular weight for the catalysts. Nevertheless, due to the smaller space requirement of the hydrogen transfer reaction than the propylene insertion reaction, the variations in the difficulty of the hydrogen transfer reactions were relatively small. These results show that the variation trend for the activation energy of the hydrogen transfer reaction in different kinds of active centers is consistent with that of the propylene insertion reaction, with the variation range slightly different from each other.

## 4. Conclusions

We prepared ZN catalysts for propylene polymerization using the mixtures of racemate and mesomer isomers of HDDB as IDs. It was observed that the *meso*-isomers preferred to be adsorbed on the catalyst. As the relative content of *meso*-isomer increased, there was a corresponding rise in the total amount of IDs adsorbed on the catalysts. This increase was concomitant with the improvements of isotacticity, catalytic activity, and average molecular weight, indicating that the mesomer ID enhances the performance of the ZN catalyst, surpassing the benefits derived from racemate isomer.

The combination of DFT calculation results from our previous paper [49] with the current study shows a remarkable alignment between the calculated stereoselectivity of PDDB and HDDB stereoisomers and the experimental findings. Notably, the orientation of the 2,4-methyl or 3,5-ethyl group in the optimized structures of MgCl_2_-TiCl_4_-ID varied significantly when employing mesomer or racemate as the ID, which was the critical factor for the catalytic performances, especially for *re*- and *si*-insertion reactions.

After carrying out the binary linear regression analysis for the experimental macroscopic activity of the catalyst and the content of ID molecules, it is evident that the apparent activity of the catalyst was proportional to the ID content, indicating that the ID content was also proportional to the number of active centers. The results showed that the activity of the single *RS* active center was lower than that of the single *SS* active center, which was consistent with the activation energy obtained from DFT calculations. These results revealed that the catalytic activity of polypropylene (PP) catalysts is dependent on both the active center content and the catalytic activity of the individual active center. Additionally, the calculated activation energy for the hydrogen transfer reaction consistently corresponds to the trend observed for the relative values of molecular weight. These results show that the variation trend for the activation energy of the hydrogen transfer reaction in different kinds of active centers is consistent with that of the propylene insertion reaction, with the variation ranges slightly different from each other.

In summary, our comprehensive analysis, incorporating factors such as the adsorption energy of ID, the activation energy difference for *re*-/*si*- insertion reaction at the active center, the activation energy for active centers, and the activation energy for hydrogen transfer reactions calculated through our active center model, aligns remarkably well with the corresponding experimental results including the observed ID content, the relative activity of the catalyst, the stereoselectivity, and the molecular weight. The remarkable consistency supports the appropriateness of our active center model in accurately describing the microstructure of ZN catalysts, which enables us to gain a detailed understanding of the impact derived from electron donors on the performance of ZN catalysts.

Based on the research of this manuscript and our previous paper [49], some insights on designing efficient electron donors have been obtained. Firstly, the electron donors should have enough adsorption energy to be adsorbed on the (110)-facet of the MgCl_2_ support, and the conformation of the free ID molecule should be the same as that of the adsorbed ID molecule, which can produce more active centers during catalyst preparation. Secondly, the substituent groups for the ID molecule neighboring the active center should be close enough to the Ti atom, which could have an appropriate effect on the *re*/*si*-insertion reaction, and can result in higher stereoselectivity. However, the excess steric effect would break down the catalytic activity. The influences for the steric conformation of ID molecules on both steroselectivity (i.e., the *re*/*si*-insertion reaction) and catalytic activity should be considered during the design of novel electron donors.

## Figures and Tables

**Table 1:** The polymerization results for the ZN catalysts with different HDDB stereoisomers as IDs.

Entry	Mesomer (wt/%)		ID ^c^ (wt/%)	AC (kgPP/gcat)	I.I. (%)	MFR (g/10 min)	*M*_n_	*M*_w_	PD
	Compound ^a^	Catalyst ^b^							
1	97.1	99.3	14.9	54.1	98.5	1.8	69,509	482,946	6.9
2	63.0	94.6	13.2	52.6	98.4	2.2	62,469	436,287	7.0
3	48.1	87.6	10.3	47.1	97.5	3.5	57,078	424,112	7.4
4	34.6	80.2	8.6	44.7	97.1	4.6	54,724	414,143	7.6
5	25.0	68.2	7.4	40.2	96.5	6.0	53,043	406,631	7.7
6	16.3	52.5	5.1	32.6	95.4	7.3	48,547	382,967	7.9
7	1.4	15.9	2.4	20.4	92.3	9.4	47,726	380,291	8.0

^a^ relative content of *meso*-HDDB in the same weight HDDB compound added in preparation of catalyst. ^b^ relative content of *meso*-HDDB in catalyst. ^c^ content of total HDDB (including mesomer and racemate) in catalyst.

**Table 2:** Comparison between the calculated stereoselectivity for the transition state in the *si-*/*re*-insertion reaction of propylene, and the isotactic index obtained from the experiments.

ID	TS (*re*) − TS (*si*) ^a^ (kcal/mol)	Calculated Stereoselectivity (%)	I.I. (%, Experiment)
*RS*-PDDB	3.8	99.5	98.9
*SS*-PDDB	0.8	75.5	91.1
*RS*-HDDB	3.2	99.0	98.7
*SS*-HDDB	1.1	82.7	91.2

^a^ The data of PDDB have little difference to Ref. [49] because of the quasi-RRHO model used in Gibbs free energy calculation.

**Table 3:** Comparison between the calculated activation energy (kcal/mol) of *si*-insertion for the PDDB and HDDB isomers, and the calculated relative activity of *SS*/*RS*.

ID	Activation Energy of *si*-Insertion	Calculated Relative Activity of *SS*/*RS*
*RS*-PDDB	11.3	1.2
*SS*-PDDB	11.2	
*RS*-HDDB	11.8	3.2
*SS*-HDDB	11.0	

**Table 4:** The relative ratio of the activity regression values of individual active centers.

ID	*A_SS_*	*A_RS_*	Regression Coefficient	*A_SS_*/*A_RS_*
PDDB	6.3	4.9	0.9992	1.3
HDDB	9.7	3.7	0.9994	2.6

**Table 5:** The adsorption energy (kcal/mol) of internal electron donors on the (110)-facet of MgCl_2_ and TiCl_4_/MgCl_2_.

Adsorption Structure	PDDB ^a^	HDDB
MgCl_2_/2*RS*	96.3	97.9
MgCl_2_/2*SS*	88.5	87.7
TiCl_4_/MgCl_2_/2*RS*	100.5	103.2
TiCl_4_/MgCl_2_/2*SS*	94.4	94.5

^a^ The data of PDDB have little difference to reference [49] because of the different models of processing frequency used in the Gibbs free energy calculation (calculated at 298.15 K and 1 atm).

**Table 6:** Activation energy (kcal/mol) of hydrogen transfer reaction.

ID	∆*G*_H_	∆*G*_H_ − ∆*G*_p_
*RS*-PDDB	16.56	5.22
*SS*-PDDB	16.36	5.16
*RS*-HDDB	16.92	5.09
*SS*-HDDB	15.96	4.94

## Data Availability

The data presented in this study are available on request from the corresponding author.

## Supplementary Materials

The following supporting information can be downloaded at: https://www.mdpi.com/article/10.3390/polym16040559/s1, Figure S1. ^1^H NMR of meso-HDDB. Figure S2. ^1^H NMR of rac-HDDB. Figure S3. LC of Entry1 compound. Figure S4. LC of Entry1 catalyst. Figure S5. LC of Entry2 compound. Figure S6. LC of Entry2 catalyst. Figure S7. LC of Entry3 compound. Figure S8. LC of Entry3 catalyst. Figure S9. LC of Entry4 compound. Figure S10. LC of Entry4 catalyst. Figure S11. LC of Entry5 compound. Figure S12. LC of Entry5 catalyst. Figure S13. LC of Entry6 compound. Figure S14. LC of Entry6 catalyst. Figure S15. LC of Entry7 compound. Figure S16. LC of Entry7 catalyst Figure S17. GPC of Entry 1 PP powder. Figure S18. GPC of Entry 2 PP powder. Figure S19. GPC of Entry 3 PP powder Figure S20. GPC of Entry 4 PP powder. Figure S21. GPC of Entry 5 PP powder. Figure S22. GPC of Entry 6 PP powder. Figure S23. GPC of Entry 7 PP powder. Table S1. The polymerization results for the catalysts with different PDDB stereoisomers as ID. NMR, LC, GPC, and DFT calculation data (PDF).

## References

[1] (1955). Method of Selectively Polymerising α-olefins.

[2] (1968). Separation of Macromolecules Having Different Stereoregularities from Polypropylenes Comprising Mixtures of Such Macromolecules.

[3] Kumawat J., Gupta V.K. (2020). Fundamental Aspects of Heterogeneous Ziegler-Natta Olefin Polymerization Catalysis: An Experimental and Computational Overview. Polym. Chem..

[4] Pasquini N. (2005). Polypropylene Handbook.

[5] Kaminsky W. (2013). Polyolefins: 50 Years after Ziegler and Natta I—Polyethylene and Polypropylene.

[6] Morini G., Albizzati E., Balbontin G., Mingozzi I., Sacchi M.C., Forlini F., Tritto I. (1996). Microstructure Distribution of Polypropylenes Obtained in the Presence of Traditional Phthalate/Silane and Novel Diether Donors:  A Tool for Understanding the Role of Electron Donors in MgCl_2_-Supported Ziegler-Natta Catalysts. Macromolecules.

[7] Sacchi M.C., Forlini F., Tritto I., Locatelli P., Morini G., Noristi L., Albizzati E. (1996). Polymerization Stereochemistry with Ziegler-Natta Catalysts Containing Dialkylpropane Diethers:  A Tool for Understanding Internal/External Donor Relationships. Macromolecules.

[8] Cecchin G., Morini G., Pelliconi A. (2001). Polypropene Product Innovation by Reactor Granule Technology. Macromol. Symp..

[9] Zaccaria F., Vittoria A., Correa A., Ehm C., Budzelaar P.H.M., Busico V., Cipullo R. (2018). Internal Donors in Ziegler-Natta Systems: Is Reduction by AlR_3_ a Requirement for Donor Clean-Up?. ChemCatChem.

[10] (2008). Solid Catalyst Component for Polymerization of Olefins, Catalyst Comprising the Same and Use Thereof.

[11] Gao M., Liu H., Wang J., Li C., Ma J., Wei G. (2004). Novel MgCl_2_-Supported Catalyst Containing Diol Dibenzoate Donor for Propylene Polymerization. Polymer.

[12] Sun Z., Liu H., Gao M. (2010). Performance of Catalyst with Different 2,4-Pentanediol Diester as Internal Donor. Petrochem. Technol..

[13] (2013). Olefin Polymerization Catalyst and Preparation Method and Use Thereof.

[14] Li H.S., Yi J.J., Cui C.M. (2008). Bis(Trifluoromethylsulfonyl)-Phenylamines as Internal Donors for Ziegler-Natta Polymerization Catalysts. China Pet. Process. Petrochem. Technol..

[15] Wang L., Yin B.Z., Yi J.J., Cui C.M. (2013). Propylene Polymerization Catalysts with Sulfonyl Amines as Internal Electron Donors. China Pet. Process. Petrochem. Technol..

[16] Matta A., Chammingkwan P., Singh B.K., Terano M., Kaneko T., Taniike T. (2018). Truxillic and Truxinic Acid-based, Bio-derived Diesters as Potent Internal Donor in Ziegler-Natta Catalyst for Propylene Polymerization. Appl. Catal. A-Genet..

[17] Patil H., Karthikeyan S., Kote V., Sengupta P., Samanta P., Kadam P., Natarajan Venkateswaran N., Gupta V. (2023). An Insight into Ziegler-Natta Catalyst Active Site Distribution for Polyolefins: Application of Jitter Differential Evolution. Polym. Bull..

[18] Piovano A., Wada T., Amodio A., Takasao G., Ikeda T., Zhu D., Terano M., Chammingkwan P., Groppo E., Taniike T. (2021). Formation of Highly Active Ziegler-Natta Catalysts Clarified by a Multifaceted Characterization Approach. ACS Catal..

[19] Shen X., Hu J., Fu Z., Lou J., Fan Z. (2013). Counting the Number of Active Centers in MgCl_2_-Supported Ziegler-Natta Catalysts by Quenching with 2-Thiophenecarbonyl Chloride and Study on the Initial Kinetics of Propylene Polymerization. Catal. Commun..

[20] Weng Y., Jiang B., Fu Z., Fan Z. (2018). Mechanism of Internal and External Electron Donor Effects on Propylene Polymerization with MgCl_2_-Supported Ziegler-Natta Catalyst: New Evidences Based on Active Center Counting. J. Appl. Polym. Sci..

[21] Yakimov A., Xu J., Searles K., Liao W., Antinucci G., Friederichs N., Busico V., Copéret C. (2021). DNP-SENS Formulation Protocols to Study Surface Sites in Ziegler-Natta Catalyst MgCl_2_ Supports Modified with Internal Donors. J. Phys. Chem. C.

[22] Bahri-Laleh N., Hanifpour A., Mirmohammadi S.A., Poater A., Nekoomanesh-Haghighi M., Talarico G., Cavallo L. (2018). Computational Modeling of Heterogeneous Ziegler-Natta Catalysts for Olefins Polymerization. Prog. Polym. Sci..

[23] Credendino R., Busico V., Causà M., Barone V., Budzelaar P.H.M., Zicovich-Wilson C. (2009). Periodic DFT Modeling of Bulk and Surface Properties of MgCl_2_. Phys. Chem. Chem. Phys..

[24] Stukalov D.V., Zilberberg I.L., Zakharov V.A. (2009). Surface Species of Titanium(IV) and Titanium(III) in MgCl_2_-Supported Ziegler-Natta Catalysts. A Periodic Density Functional Theory Study. Macromolecules.

[25] Stukalov D.V., Zakharov V.A. (2009). Active Site Formation in MgCl_2_–Supported Ziegler-Natta Catalysts. A Density Functional Theory Study. J. Phys. Chem. C.

[26] Brambilla L., Zerbi G., Piemontesi F., Nascetti S., Morini G. (2007). Structure of MgCl_2_–TiCl_4_ Complex in Co-milled Ziegler-Natta Catalyst Precursors with Different TiCl_4_ Content: Experimental and Theoretical Vibrational Spectra. J. Mol. Catal. A Chem..

[27] D’Amore M., Credendino R., Budzelaar P.H.M., Causá M., Busico V. (2012). A Periodic Hybrid DFT Approach (Including Dispersion) to MgCl_2_–Supported Ziegler-Natta Catalysts–1: TiCl_4_ Adsorption on MgCl_2_ Crystal Surfaces. J. Catal..

[28] Breuza E., Antinucci G., Budzelaar P.H.M., Busico V., Correa A., Ehm C. (2018). MgCl_2_-Supported Ziegler-Natta Catalysts: A DFT-D “Flexible-Cluster” Approach to Internal Donor Adducts. J. Phys. Chem. C.

[29] Vanka K., Singh G., Iyer D., Gupta V.K. (2010). DFT Study of Lewis Base Interactions with the MgCl_2_ Surface in the Ziegler-Natta Catalytic System: Expanding the Role of the Donors. J. Phys. Chem. C.

[30] Cavallo L., Del Piero S., Ducéré J.-M., Fedele R., Melchior A., Morini G., Piemontesi F., Tolazzi M. (2007). Key Interactions in Heterogeneous Ziegler-Natta Catalytic Systems:  Structure and Energetics of TiCl_4_–Lewis Base Complexes. J. Phys. Chem. C..

[31] Cavallo L., Ducéré J.M., Fedele R., Melchior A., Mimmi M.C., Morini G., Piemontesi F., Tolazzi M. (2008). Ziegler-Natta Catalytic Systems: Calorimetric and DFT Study on TiCl_4_-Lewis Base Interactions. J. Therm. Anal. Calorim..

[32] Cavallo L., Fedele R., Morini G., Ducéré J.-M., Melchior A., Correa A., Piemontesi F., Tolazzi M. (2007). An Empirical Correction Term to Density Functional Theory for the Description of the TiCl_4_-Lewis Base Complexes. Macromol. Symp..

[33] Bahri-Laleh N., Nekoomanesh-Haghighi M., Mirmohammadi S.A. (2012). A DFT Study on the Effect of Hydrogen in Ethylene and Propylene Polymerization Using a Ti-Based Heterogeneous Ziegler-Natta Catalyst. J. Organomet. Chem..

[34] Bahri-Laleh N. (2016). Interaction of Different Poisons with MgCl_2_/TiCl_4_ Based Ziegler-Natta Catalysts. Appl. Surf. Sci..

[35] Guo X., Shao Y., Luo J., Liu Z., Liu B. (2022). The Atomic Defects on the (104) and (110) Surfaces of the MgCl_2_-Supported Ziegler-Natta Catalyst: A Periodic DFT Study. Catal. Sci. Technol..

[36] Credendino R., Liguori D., Fan Z., Morini G., Cavallo L. (2015). Toward a Unified Model Explaining Heterogeneous Ziegler-Natta Catalysis. ACS Catal..

[37] Guo X., Cui L., Wang Y., Yi J., Sun J., Liu Z., Liu B. (2021). Mechanistic Study on Effect of Electron Donors in Propylene Polymerization Using the Ziegler-Natta Catalyst. J. Phys. Chem. C.

[38] Guo X., Cui L., Yi J., Liu Z., Liu B. (2022). Understanding the Role of Sulfonyl Amine Donors in Propylene Polymerization Using MgCl_2_-Supported Ziegler-Natta Catalyst. J. Phys. Chem. C.

[39] Guo X., Liu Z., Fan Z., Liu B. (2023). New Insights into the Nature of Ti(II) and Ti(III) Active Sites in the Heterogeneous Ziegler-Natta Catalyst. J. Phys. Chem. C.

[40] Kumawat J., Kumar Gupta V., Vanka K. (2014). The Nature of the Active Site in Ziegler-Natta Olefin Polymerization Catalysis Systems—A Computational Investigation. Eur. J. Inorg. Chem..

[41] Taniike T., Terano M. (2012). Coadsorption Model for First-Principle Description of Roles of Donors in Heterogeneous Ziegler-Natta Propylene Polymerization. J. Catal..

[42] Ratanasak M., Parasuk V. (2016). Understanding the Roles of Novel Electron Donors in Ziegler-Natta Catalyzed Propylene Polymerization. RSC Adv..

[43] Milanesi M., Piovano A., Wada T., Zarupski J., Chmamingkwan P., Taniike T., Groppo E. (2023). Influence of the Synthetic Procedure on the Properties of Three Ziegler-Natta Catalysts with the same 1,3-Diether Internal Donor. Catal. Today.

[44] Nikolaeva M., Mikenas T., Matsko M., Zakharov V. (2016). Effect of AlEt3 and an External Donor on the Distribution of Active Sites According to Their Stereospecificity in Propylene Polymerization over TiCl_4_/MgCl_2_ Catalysts with Different Titanium Content. Macromol. Chem. Phys..

[45] Vittoria A., Antinucci G., Zaccaria F., Cipullo R., Busico V. (2020). Monitoring the Kinetics of Internal Donor Clean-up from Ziegler-Natta Catalytic Surfaces: An Integrated Experimental and Computational Study. J. Phys. Chem. C.

[46] Khatri V., Sahoo U., Kaur S., Rani R., Singh G., Kapur G.S., Kashyap H. (2020). Control of Ziegler-Natta Catalyst Activity by the Structural Design of Alkoxysilane-Based External Donors. New J. Chem..

[47] Raj K., Gupta V., Vanka K. (2023). The Potential Role of Lewis Acid–Base Adducts in Enhancing Stereoselectivity in Ziegler-Natta Catalysts: A DFT Study. J. Phys. Chem. C.

[48] (2015). Catalyst Component for Olefin Polymerization Reaction and Catalyst Comprising Same.

[49] Li C., Feng H., Liu H., Zhuang Z., Zhou J., Liu D. (2023). Effect of Dioldibenzoate Isomers as Electron Donors on the Performances of Ziegler-Natta Polypropylene Catalysts: Experiments and Calculations. J. Phys. Chem. C.

[50] (2018). Plastics, Thermoplastic—Determination of Melt Mass Flow Rate (MFR) and Melt Volume Flow Rate (MVR), Part 1: Standard Method.

[51] Adamo C., Barone V. (1999). Toward Reliable Density Functional Methods without Adjustable Parameters: The PBE0 Model. J. Chem. Phys..

[52] Perdew J.P., Burke K., Ernzerhof M. (1996). Generalized Gradient Approximation Made Simple. Phys. Rev. Lett..

[53] Grimme S. (2006). Semiempirical GGA-type Density Functional Constructed with a Long-Range Dispersion Correction. J. Comput. Chem..

[54] Grimme S. (2012). Supramolecular Binding Thermodynamics by Dispersion-Corrected Density Functional Theory. Chem.-Eur. J..

[55] Lu T., Chen X. (2021). Shermo: A General Code for Calculating Molecular Thermochemistry Properties. Comput. Theor. Chem..

[56] Chadwick J.C., Heere J.J.R., Sudmeijer O. (2000). Factors Influencing Chain Transfer with Monomer and with Hydrogen in Propene Polymerization Using MgCl_2_-Supported Ziegler-Natta Catalysts. Macromol. Chem. Phys..

[57] Kojoh S., Tsutsui T., Kashiwa N., Itoh M., Mizuno A. (1998). Effect of An External Donor upon Chain-transfer Reactions in Propylene Polymerization with a MgCl_2_-Supported Titanium Catalyst System. Polymer.

[58] Cavallo L., Guerra G. (1996). A Density Functional and Molecular Mechanics Study Of β-Hydrogen Transfer in Homogeneous Ziegler-Natta Catalysis. Macromolecules.

[59] Talarico G., Budzelaar P.H.M. (2008). Variability of Chain Transfer to Monomer Step in Olefin Polymerization. Organometallics.

[60] Busico V., Cipullo R., Pellecchia R., Ronca S., Roviello G., Talarico G. (2006). Design of Stereoselective Ziegler-Natta Propene Polymerization Catalysts. Proc. Natl. Acad. Sci. USA.

[61] Gibson V., Spitzmesser S.K. (2003). Advances in Non-Metallocene Olefin Polymerization Catalysis. Chem. Rev..

[62] Yu Y., Fu Z., Fan Z. (2012). Chain Transfer Reactions of Propylene Polymerization Catalyzed by AlEt_3_ Activated TiCl_4_/MgCl_2_ Catalyst Under very Low Monomer Addition Rate. J. Mol. Catal. A Chem..

[63] Kissin Y.V., Rishina L.A., Vizen E.I. (2002). Hydrogen Effects in Propylene Polymerization Reactions with Titanium-Based Ziegler-Natta Catalysts. II. Mechanism of the Chain-Transfer Reaction. J. Polym. Sci. Pol. Chem..

