# Supplementary material for: Experimental and Theoretical Insights into the Effect of Dioldibenzoate Isomers on the Performance of Polypropylene Catalysts
Source: Polymers (Basel). 2024 Feb 19;16(4):559. doi: 10.3390/polym16040559 (PMC10892235; doi:10.3390/polym16040559)
Supplement: Supplementary file 1 [file polymers-16-00559-s001.zip › polymers-2843891-supplementary.pdf]

Table of contents

1. ^{1}H NMR of HDDB compound ··· S2
2. The polymerization results for the catalysts with different PDDB stereoisomers ··· S3
3. LC of compound and catalyst ··· S4
4. GPC of PP powder ··· S11
5. Cartestian Coordinates(Å) for the optimized and TS structures ··· S15

1. ^{1}H NMR of HDDB compound($CDCl_3$, 300 MHz)

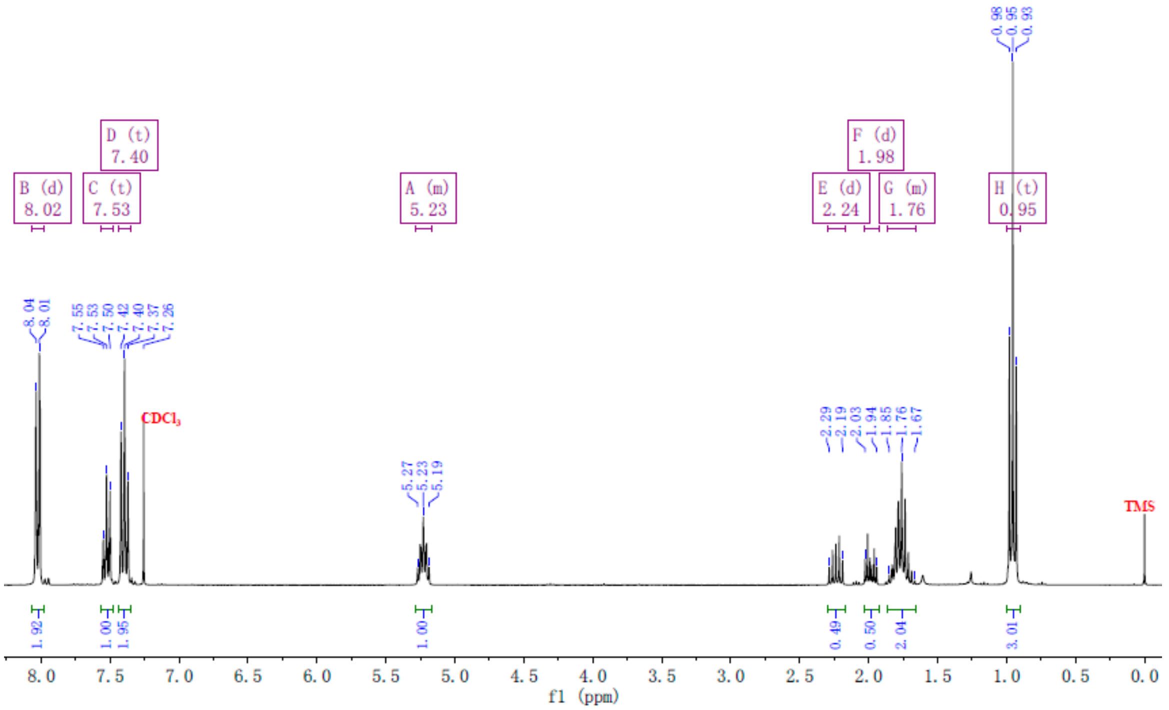

Figure S1. ^{1}H NMR of meso-HDDB.

δ 0.95 (t, J = 7.4 Hz, 6H), 1.66-1.86 (m, 4H), 1.98(dt, J = 14.5, 5.4 Hz, 1H), 2.24(dt, J = 14.6, 7.3 Hz, 1H), 5.17-5.29 (m, 2H), 7.40 (t, J = 7.5 Hz, 4H), 7.53 (t, J = 7.4 Hz, 2H), 8.02 (d, J = 7.0 Hz, 4H).

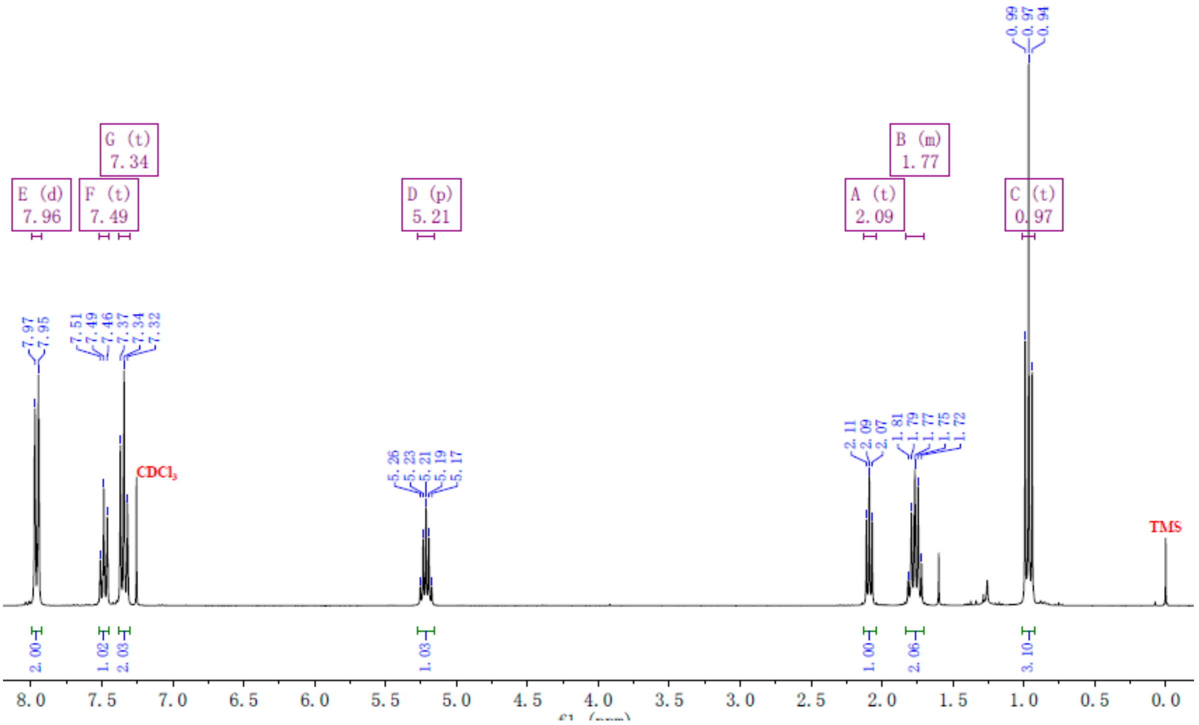

Figure S2. ^{1}H NMR of rac-HDDB.

δ 0.97 (t, J = 7.4 Hz, 6H), 1.70-1.83(m, 4H), 2.09 (t, J = 6.1 Hz, 2H), 5.21 (p, J = 6.1 Hz, 2H), 7.34 (t, J = 7.6 Hz, 4H), 7.49 (t, J = 7.4 Hz, 2H), 7.96 (d, J = 7.0 Hz, 2H).

2. The polymerization results for the catalysts with different PDDB stereoisomers

Table S1. The polymerization results for the catalysts with different PDDB stereoisomers as ID.

Entry	mesomer (wt/%)		ID (wt/%)	AC (kgPP/gcat)	I.I. (%)	MFR (g/10min)	M_n	M_w	PD
	Compound	Catalyst							
1	96.2	98.3	11.9	59.2	98.7	1.0	72856	537635	7.4
2	65.2	89.3	11.3	57.7	98.3	1.8	72603	534948	7.4
3	52.9	75.8	11.0	55.7	97.9	2.5	68286	530360	7.8
4	42.1	65.7	9.2	51.6	97.6	3.2	64902	512170	7.9
5	28.5	54.7	8.3	45.9	97.0	4.4	57343	456695	8.0
6	21.5	51.6	7.1	42.8	96.6	4.9	56986	454862	8.0
7	16.3	38.4	5.8	30.5	95.5	6.0	43362	361405	8.3
8	2.1	34.6	3.7	23.2	93.9	8.3	42799	364760	8.5

3. LC of compound and catalyst

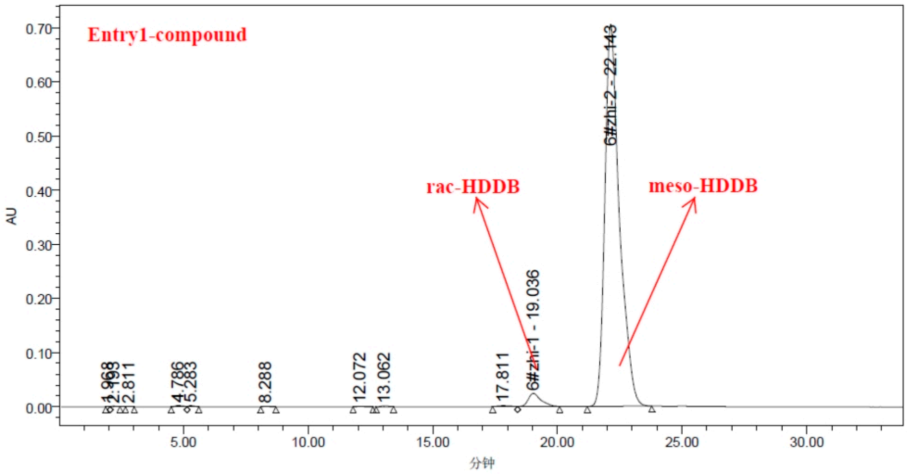

色谱峰结果

	名字	保留时间	面积	峰高	含量	单位
1		1.968	4307	741		
2		2.193	11177	792		
3		2.811	6577	690		
4		4.786	44139	2326		
5		5.283	28950	2136		
6		8.288	9455	558		
7		12.072	14386	625		
8		13.062	26050	1310		
9		17.811	49584	1698		
10	6#zhi-1	19.036	863601	24362		
11	6#zhi-2	22.143	28687173	706252		

Result

	Name	RT	Area	Peak height	Content	Unit
1						
2						
3						
4						
5						
6						
7						
8						
9						
10	6#zhi-1					
11	6#zhi-2					

Figure S3. LC of Entry1 compound.

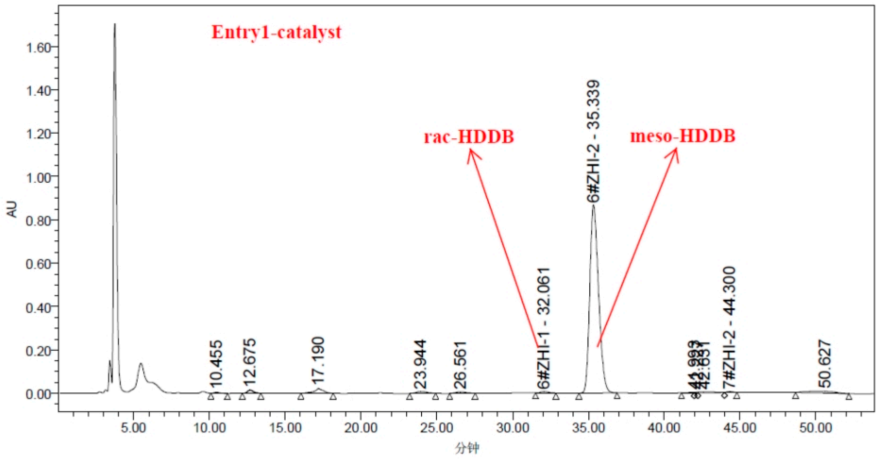

色谱峰结果

		名字	保留时间	面积	峰高	含量	单位
	1		10.455	112497	4822		
	2		12.675	379000	14531		
	3		17.190	900515	21838		
	4	DIBP	19.292				
	5	DNBP	21.136				
	6		23.944	398695	8821		
	7		26.561	304994	6510		
rac-HDDB	8	6#ZHI-1	32.061	241254	7028		
meso-HDDB	9	6#ZHI-2	35.339	34959597	867795		
	10	7#ZHI-1	39.500				
	11		41.993	60784	2390		
	12		42.227	36939	2858		

Result

	Name	RT	Area	Peak height	Content	Unit
1						
2						
3						
4	DIBP					
5	DNBP					
6						
7						
8	6#ZHI-1					
9	6#ZHI-2					
10	7#ZHI-1					
11						
12						

Figure S4. LC of Entry1 catalyst.

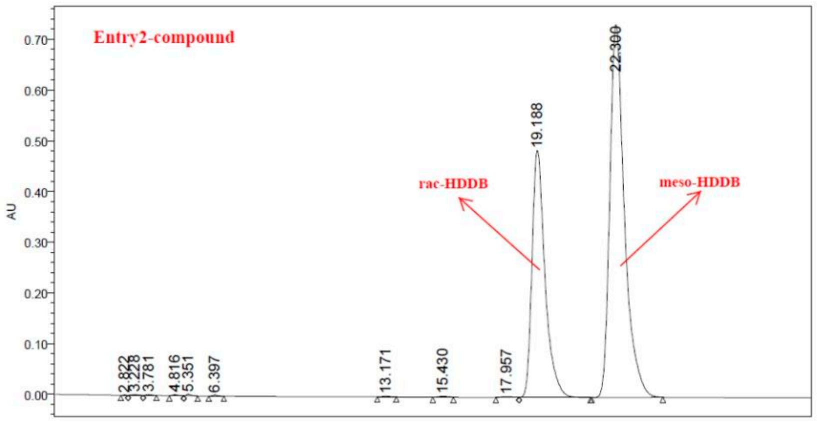

色谱峰结果

	名字	保留时间	面积	峰高	%面积	含量	单位
1		2.822	4939	453	0.01		
2		3.228	25506	1288	0.05		
3		3.781	26226	1869	0.06		
4		4.816	30061	1774	0.06		
5		5.351	39840	2985	0.08		
6		6.397	28546	2018	0.06		
7		13.171	34132	1603	0.07		
8		15.430	41239	1710	0.09		
9		17.957	53551	1820	0.11		
10		19.188	17442079	487372	36.77		
11		22.300	29703261	734997	62.63		

Result

	Name	RT	Area	Peak height	%Area	Content	Unit
1							
2							
3							
4							
5							
6							
7							
8							
9							
10							
11							

Figure S5. LC of Entry2 compound.

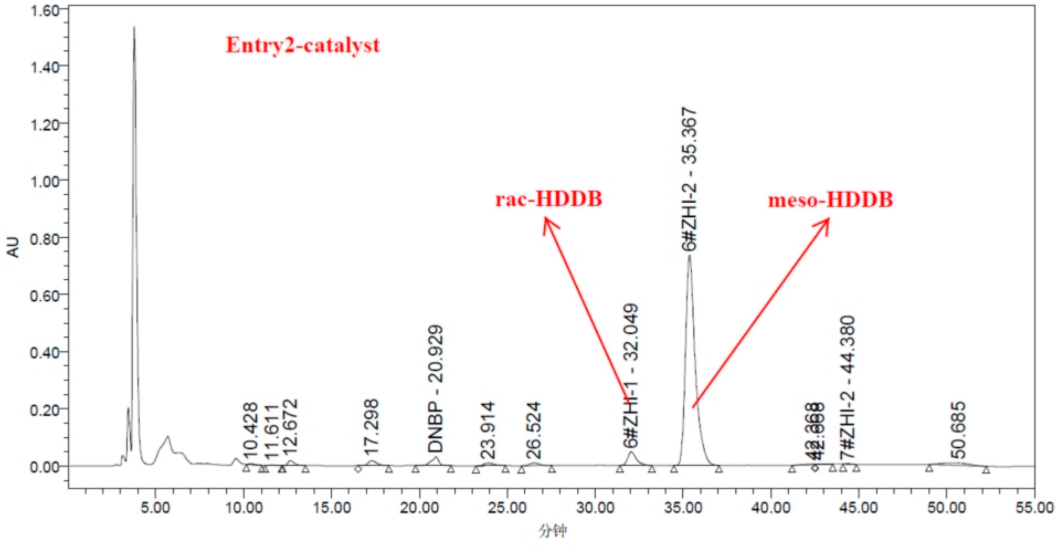

色谱峰结果

	名字	保留时间	面积	峰高	含量	单位
1		10.428	46195	2125		
2		11.611	44154	2040		
3		12.672	441256	17535		
4		17.298	598912	17615		
5	DIBP	19.292				
6	DNBP	20.929	943812	30253		
7		23.914	393215	9563		
8		26.524	385041	8969		
9	6#ZHI-1	32.049	1605332	47196		
10	6#ZHI-2	35.367	28158507	734902		
11	7#ZHI-1	39.500				
12		42.368	117686	2579		

Result

	Name	RT	Area	Peak height	Content	Unit
1						
2						
3						
4						
5	DIBP					
6	DNBP					
7						
8						
9	6#ZHI-1					
10	6#ZHI-2					
11	7#ZHI-1					
12						

Figure S6. LC of Entry2 catalyst.

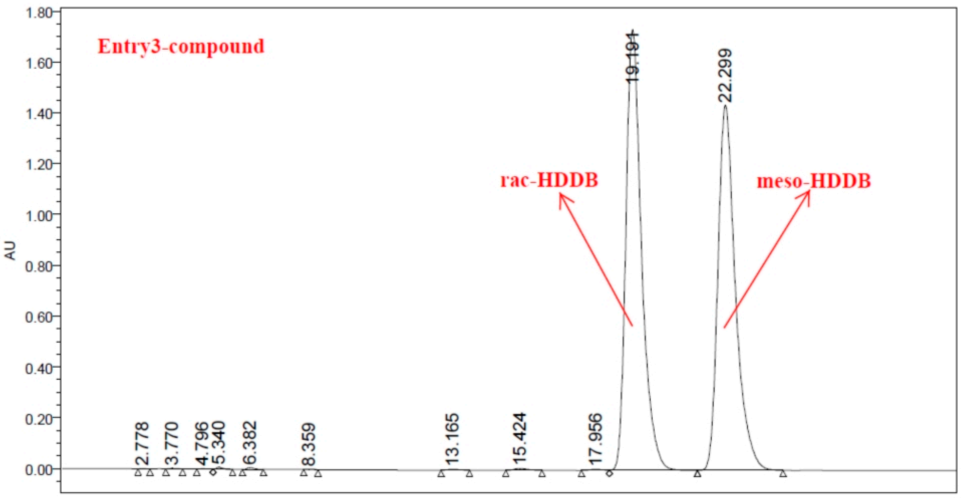

色谱峰结果

	名字	保留时间	面积	峰高	% 面积	含量	单位
1		2.778	8929	953	0.01		
2		3.770	29573	1885	0.02		
3		4.796	24725	1466	0.02		
4		5.340	98372	7333	0.08		
5		6.382	105031	7324	0.09		
6		8.359	9386	659	0.01		
7		13.165	81710	3368	0.07		
8		15.424	209875	7312	0.17		
9		17.956	112136	3800	0.09		
10		19.191	63167033	1734792	51.59		
11		22.299	58582743	1437479	47.85		

Result

	Name	RT	Area	Peak height	%Area	Content	Unit
1							
2							
3							
4							
5							
6							
7							
8							
9							
10							
11							

Figure S7. LC of Entry3 compound.

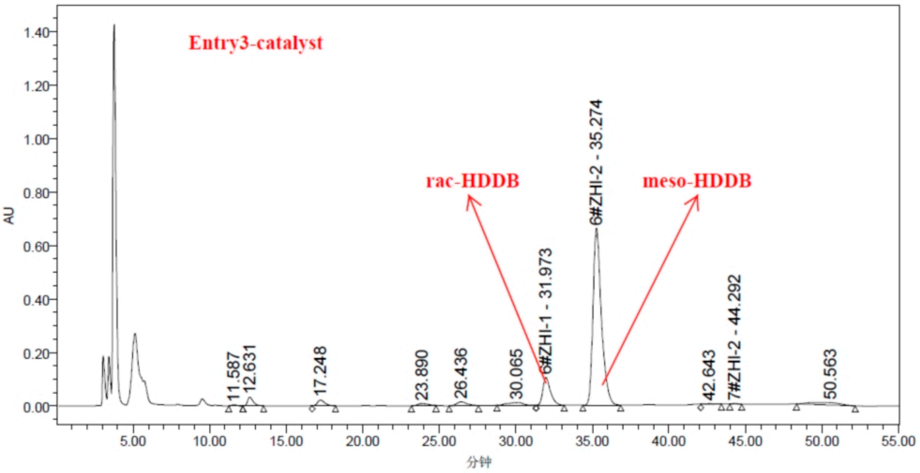

色谱峰结果

	名字	保留时间	面积	峰高	含量	单位
1		11.587	70604	3154		
2		12.631	814530	32167		
3		17.248	741740	21932		
4	DIBP	19.292				
5	DNBP	21.136				
6		23.890	368583	8716		
7		26.436	643833	14024		
8		30.065	711973	9672		
9	6#ZHI-1	31.973	3551565	103404		
10	6#ZHI-2	35.274	25184152	662117		
11	7#ZHI-1	39.500				
12		42.643	132619	2561		

Result

	Name	RT	Area	Peak height	Content	Unit
1						
2						
3						
4	DIBP					
5	DNBP					
6						
7						
8						
9	6#ZHI-1					
10	6#ZHI-2					
11	7#ZHI-1					
12						

Figure S8. LC of Entry3 catalyst.

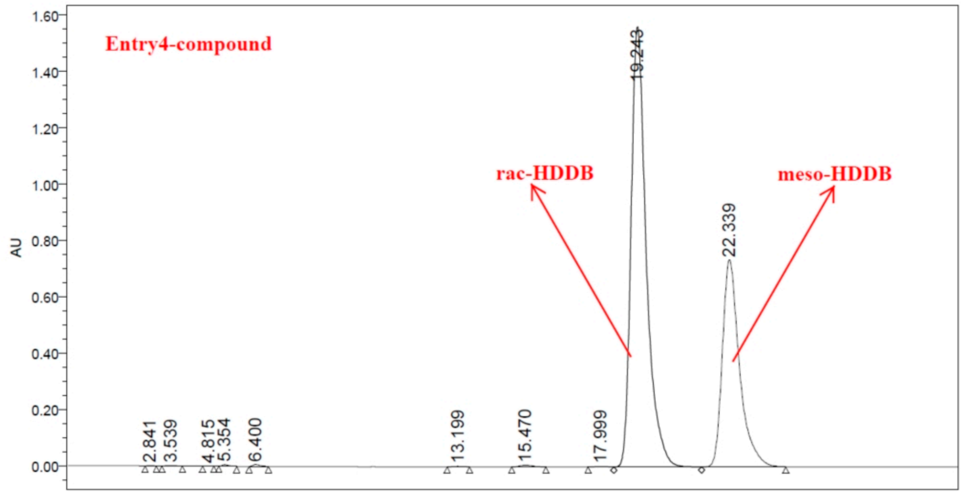

色谱峰结果

	名字	保留时间	面积	峰高	%面积	含量	单位
1		2.841	6489	560	0.01		
2		3.539	26979	1523	0.03		
3		4.815	6274	549	0.01		
4		5.354	70504	5445	0.08		
5		6.400	97120	6736	0.11		
6		13.199	37756	1767	0.04		
7		15.470	177041	6402	0.21		
8		17.999	54850	1943	0.06		
9		19.243	55553953	1563136	64.99		
10		22.339	29443833	735214	34.45		

Result

	Name	RT	Area	Peak height	%Area	Content	Unit
1							
2							
3							
4							
5							
6							
7							
8							
9							
10							

Figure S9. LC of Entry4 compound.

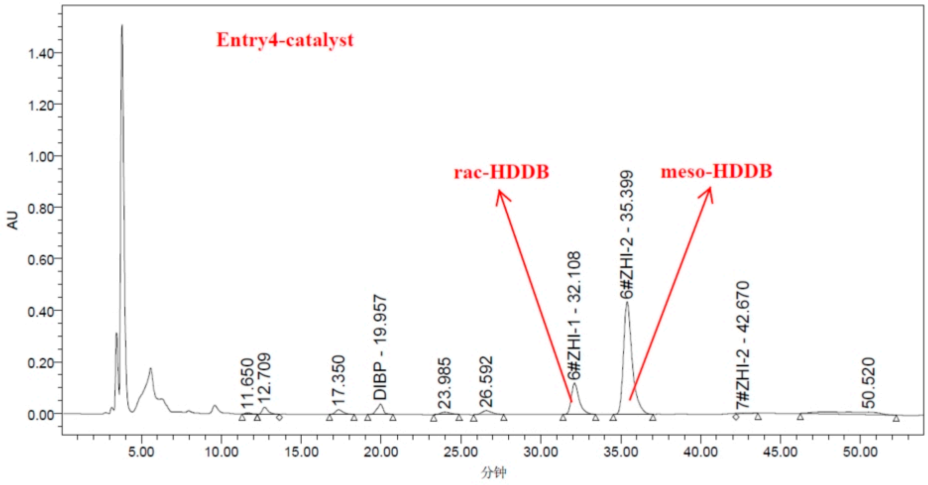

色谱峰结果

	名字	保留时间	面积	峰高	含量	单位
1		11.650	99186	4411		
2		12.709	686875	26882		
3		17.350	563190	17394		
4	DIBP	19.957	1061193	39987		
5	DNBP	21.136				
6		23.985	364404	8995		
7		26.592	642183	14581		
8	6#ZHI-1	32.108	4075720	120156		
9	6#ZHI-2	35.399	16464472	435538		
10	7#ZHI-1	39.500				
11	7#ZHI-2	42.670	151733	2708		
12	7#ZHI-3	45.500				

Result

	Name	RT	Area	Peak height	Content	Unit
1						
2						
3						
4	DIBP					
5	DNBP					
6						
7						
8	6#ZHI-1					
9	6#ZHI-2					
10	7#ZHI-1					
11	7#ZHI-2					
12	7#ZHI-3					

Figure S10. LC of Entry4 catalyst.

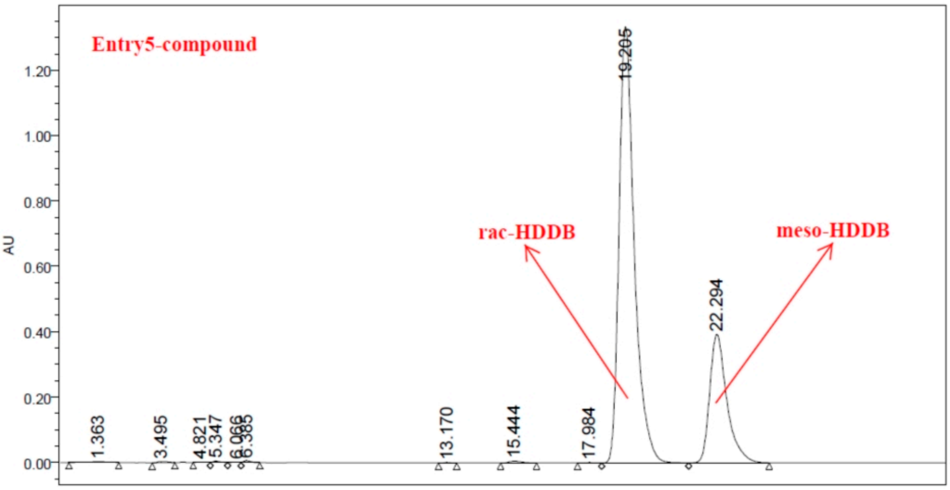

色谱峰结果

	名字	保留时间	面积	峰高	%面积	含量	单位
1		1.363	118787	1820	0.18		
2		3.495	75467	3602	0.12		
3		4.821	29879	1466	0.05		
4		5.347	67392	4437	0.10		
5		6.066	30400	1657	0.05		
6		6.385	99949	6316	0.16		
7		13.170	19277	994	0.03		
8		15.444	162700	5610	0.25		
9		17.984	30725	1137	0.05		
10		19.205	47714980	1334514	74.29		
11		22.294	15879347	393428	24.72		

Result

	Name	RT	Area	Peak height	%Area	Content	Unit
1							
2							
3							
4							
5							
6							
7							
8							
9							
10							
11							

Figure S11. LC of Entry5 compound.

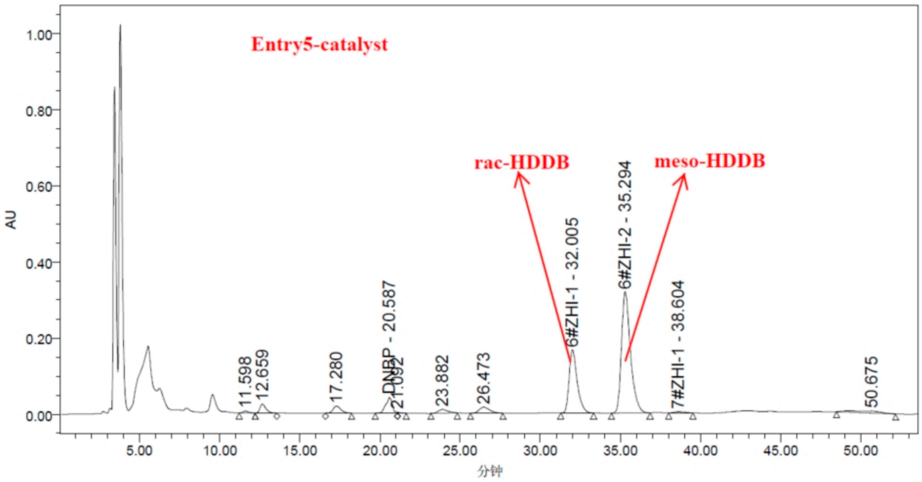

色谱峰结果

	名字	保留时间	面积	峰高	含量	单位
1		11.598	111248	4809		
2		12.659	605376	23523		
3		17.280	640418	18667		
4	DIBP	19.292				
5	DNBP	20.587	1167862	41222		
6		21.092	30652	2640		
7		23.882	396495	9281		
8		26.473	748904	16003		
9	6#ZHI-1	32.005	5775371	165484		
10	6#ZHI-2	35.294	12375498	318259		
11	7#ZHI-1	38.604	145871	3197		
12	7#ZHI-2	43.900				

Result

	Name	RT	Area	Peak height	Content	Unit
1						
2						
3						
4	DIBP					
5	DNBP					
6						
7						
8						
9	6#ZHI-1					
10	6#ZHI-2					
11	7#ZHI-1					
12	7#ZHI-2					

Figure S12. LC of Entry5 catalyst.

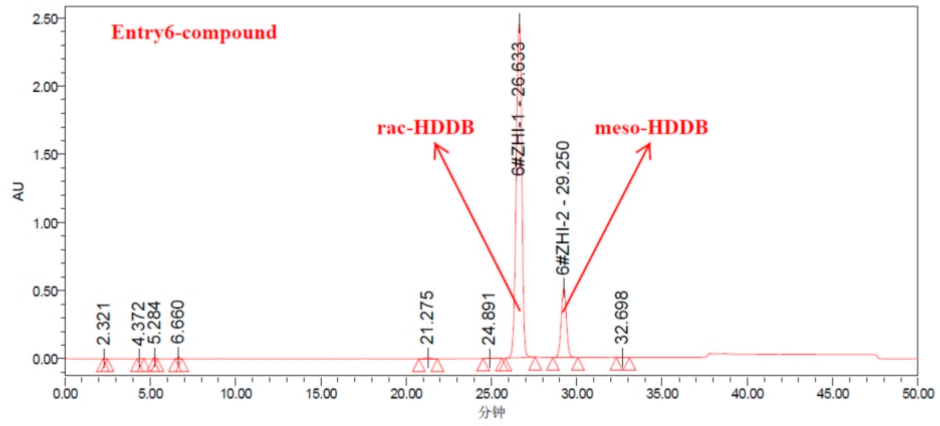

	名称	保留时间 (分钟)	面积 (微伏*秒)	高度 (微伏)	含量	单位
1		2.321	12045	2448		
2		4.372	12423	1064		
3		5.284	47024	8315		
4		6.660	116876	15478		
5	DIBP	16.200				
6	DNBP	17.800				
7		21.275	203820	7777		
8		24.891	40501	1428		
9	6#ZHI-1	26.633	55805052	2453090		
10	6#ZHI-2	29.250	10836862	512153		
11		32.698	35378	1550		
12	7#ZHI-1	35.720				
13	7#ZHI-2	38.800				

峰结果

Result

	Name	RT (min)	Area (µV.s)	Peak height (µV)	Content	Unit
1						
2						
3						
4						
5	DIBP					
6	DNBP					
7						
8						
9	6#ZHI-1					
10	6#ZHI-2					
11						
12	7#ZHI-1					
13	7#ZHI-2					

Figure S13. LC of Entry6 compound.

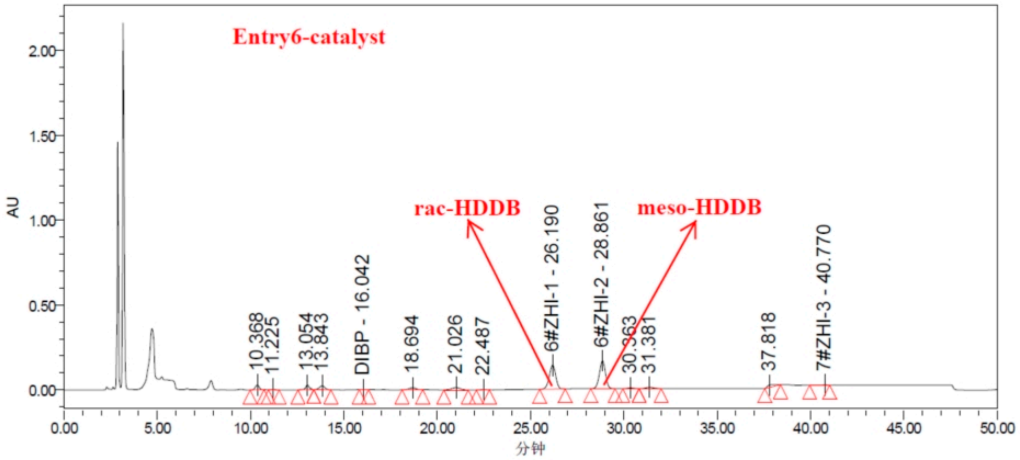

峰结果

	名称	保留时间 (分钟)	面积 (微伏*秒)	% 面积	高度 (微伏)	含量	单位
1		10.368	408691	4.10	28944		
2		11.225	41679	0.42	2951		
3		13.054	405526	4.07	27429		
4		13.843	462320	4.64	22528		
5	DIBP	16.042	15420	0.15	1008		
6	DNBP	17.200					
7		18.694	328370	3.29	12571		
8		21.026	423603	4.25	13719		
9		22.487	32668	0.33	1445		
10	6#ZHI-1	26.190	3387360	33.97	141049		
11	6#ZHI-2	28.861	3747396	37.58	164259		
12		30.363	89014	0.89	3951		
13		31.381	225983	2.27	8148		

Result

	Name	RT (min)	Area (µV.s)	%Area	Peak height (µV)	Content	Unit
1							
2							
3							
4							
5	DIBP						
6	DNBP						
7							
8							
9							
10	6#ZHI-1						
11	6#ZHI-2						
12							
13							

Figure S14. LC of Entry6 catalyst.

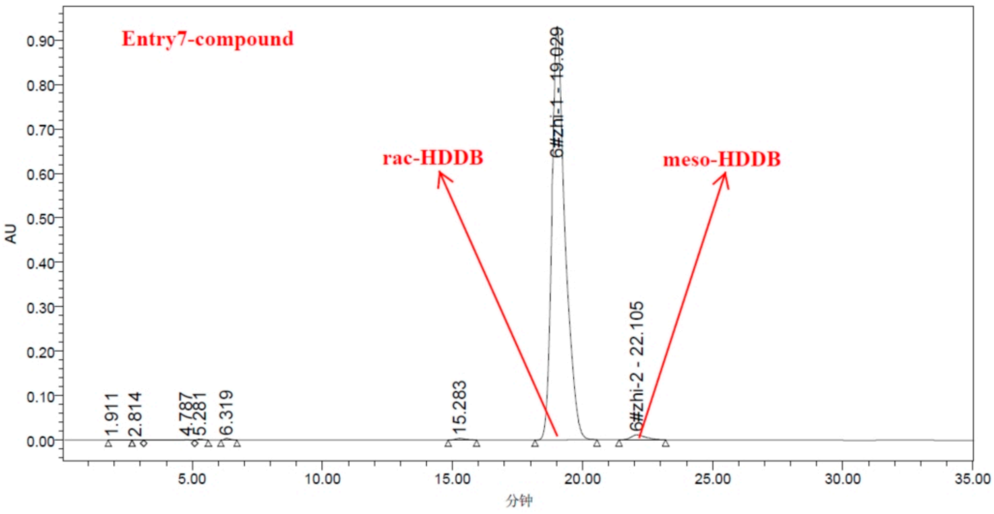

色谱峰结果

	名字	保留时间	面积	峰高	含量	单位
1		1.911	7001	378		
2		2.814	5113	487		
3		4.787	19046	542		
4		5.281	32415	2449		
5		6.319	60288	4179		
6		15.283	107716	3877		
7	6#zhi-1	19.029	33166305	930789		
8	6#zhi-2	22.105	460214	11510		

Result

	Name	RT	Area	Peak height	Content	Unit
1						
2						
3						
4						
5						
6						
7	6#zhi-1					
8	6#zhi-2					

Figure S15. LC of Entry7 compound.

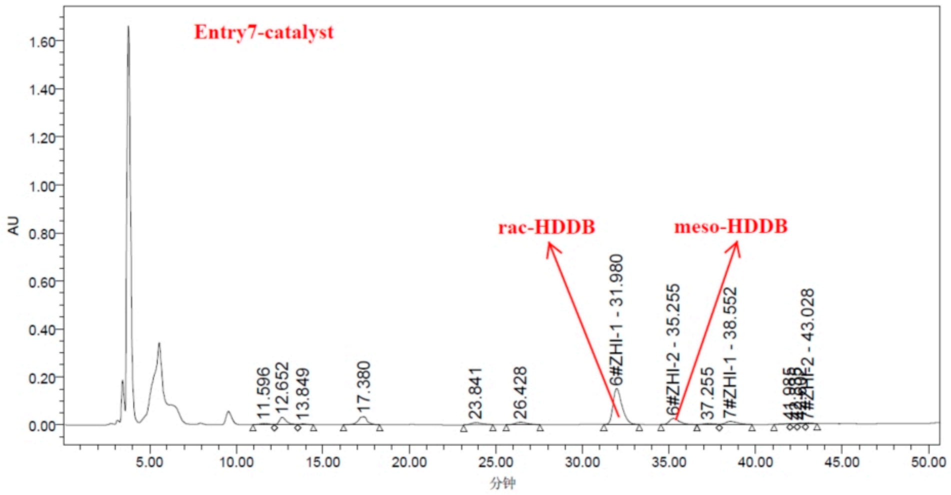

色谱峰结果

	名字	保留时间	面积	峰高	含量	单位
1		11.596	202727	5998		
2		12.652	893052	30915		
3		13.849	95084	3428		
4		17.380	1288266	33280		
5	DIBP	19.292				
6	DNBP	21.136				
7		23.841	388722	8851		
8		26.428	518022	10714		
9	6#ZHI-1	31.980	5391151	149902		
10	6#ZHI-2	35.255	1017127	25573		
11		37.255	189681	4760		
12	7#ZHI-1	38.552	639655	12904		

Result

	Name	RT	Area	Peak height	Content	Unit
1						
2						
3						
4						
5	DIBP					
6	DNBP					
7						
8						
9	6#ZHI-1					
10	6#ZHI-2					
11						
12	7#ZHI-1					

Figure S16. LC of Entry7 catalyst.

4. GPC of PP powder

MW Averages

Mp: 326471	Mn: 69509	Mv: 390403	Mw: 482946
Mz: 2002921	Mz+1: 5654590	PD: 6.9480	

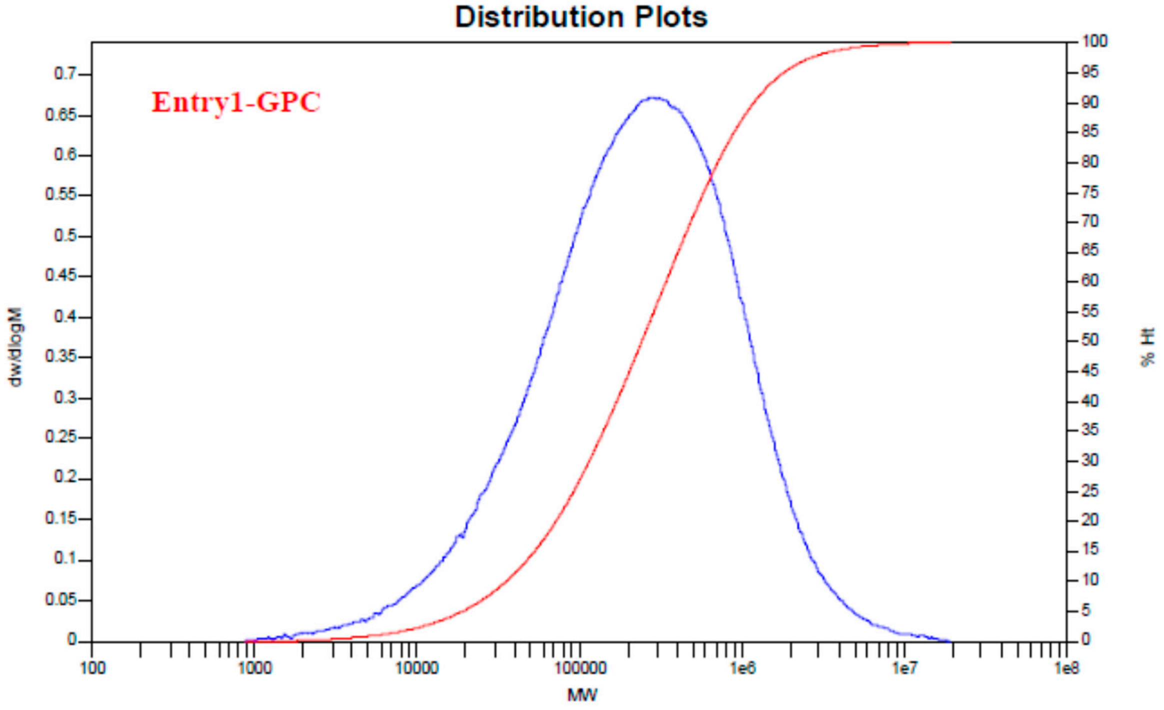

Figure S17. GPC of Entry 1 PP powder.

MW Averages

Mp: 295926	Mn: 62469	Mv: 357473	Mw: 436287
Mz: 1602093	Mz+1: 4323374	PD: 6.9841	

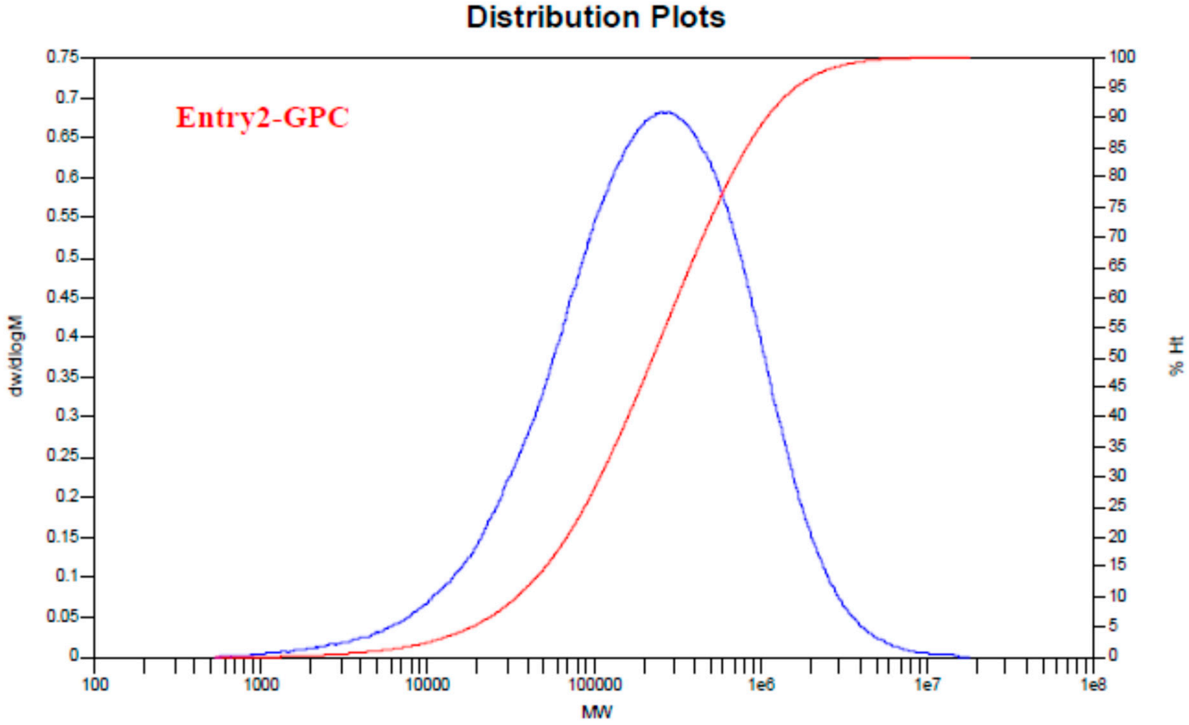

Figure S18. GPC of Entry 2 PP powder.

MW Averages

Mp: 330950	Mn: 57078	Mv: 343368	Mw: 424112
Mz: 1766472	Mz+1: 5306399	PD: 7.4304	

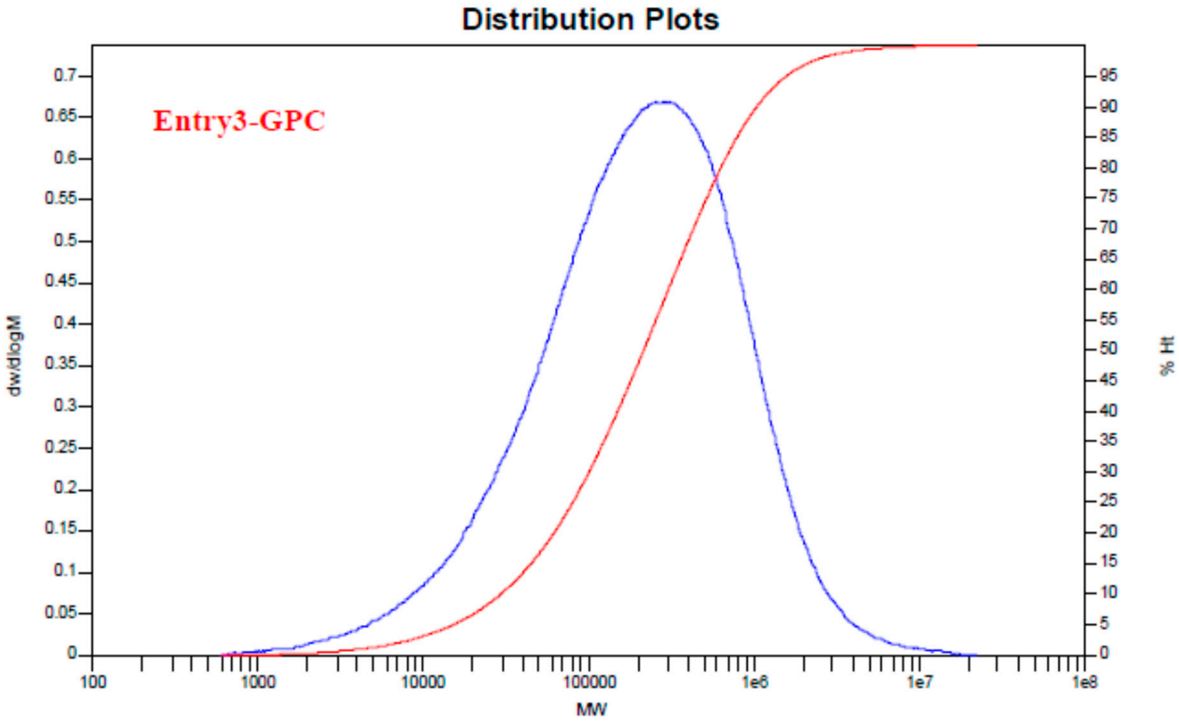

Figure S19. GPC of Entry 3 PP powder.

MW Averages

Mp: 307725	Mn: 54724	Mv: 333416	Mw: 414143
Mz: 1834867	Mz+1: 5876838	PD: 7.5678	

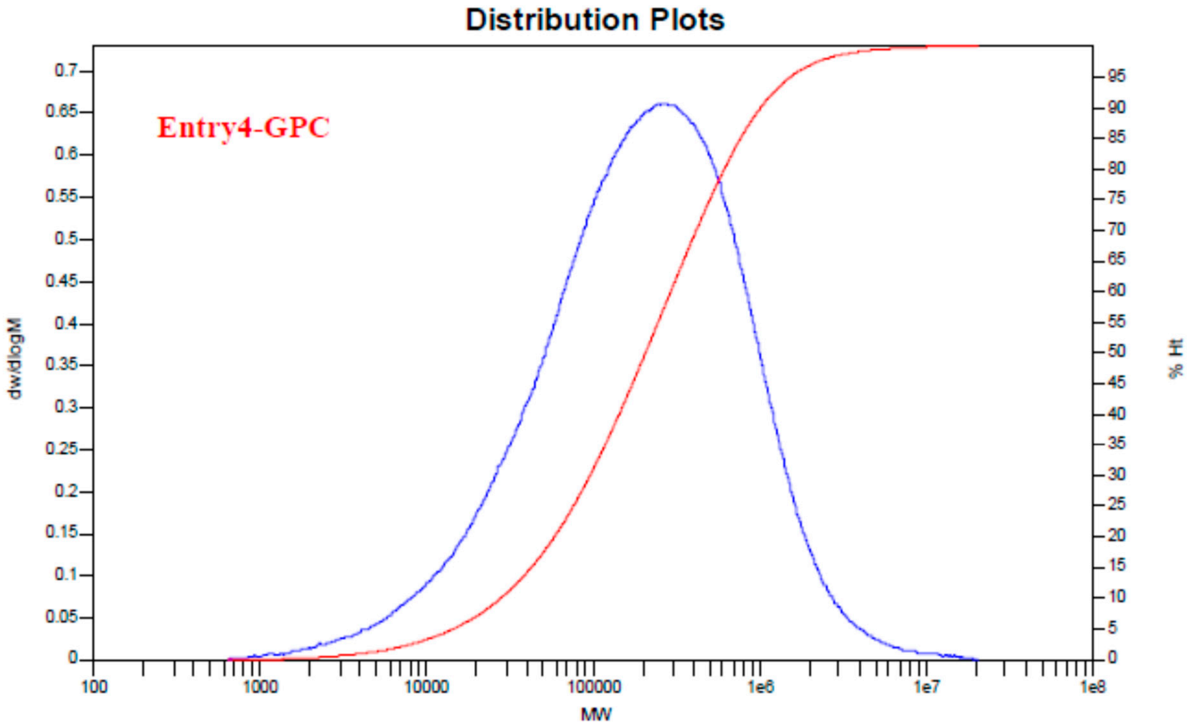

Figure S20. GPC of Entry 4 PP powder.

MW Averages

Mp: 292306	Mn: 53043	Mv: 329381	Mw: 406631
Mz: 1649441	Mz+1: 4744414	PD: 7.6661	

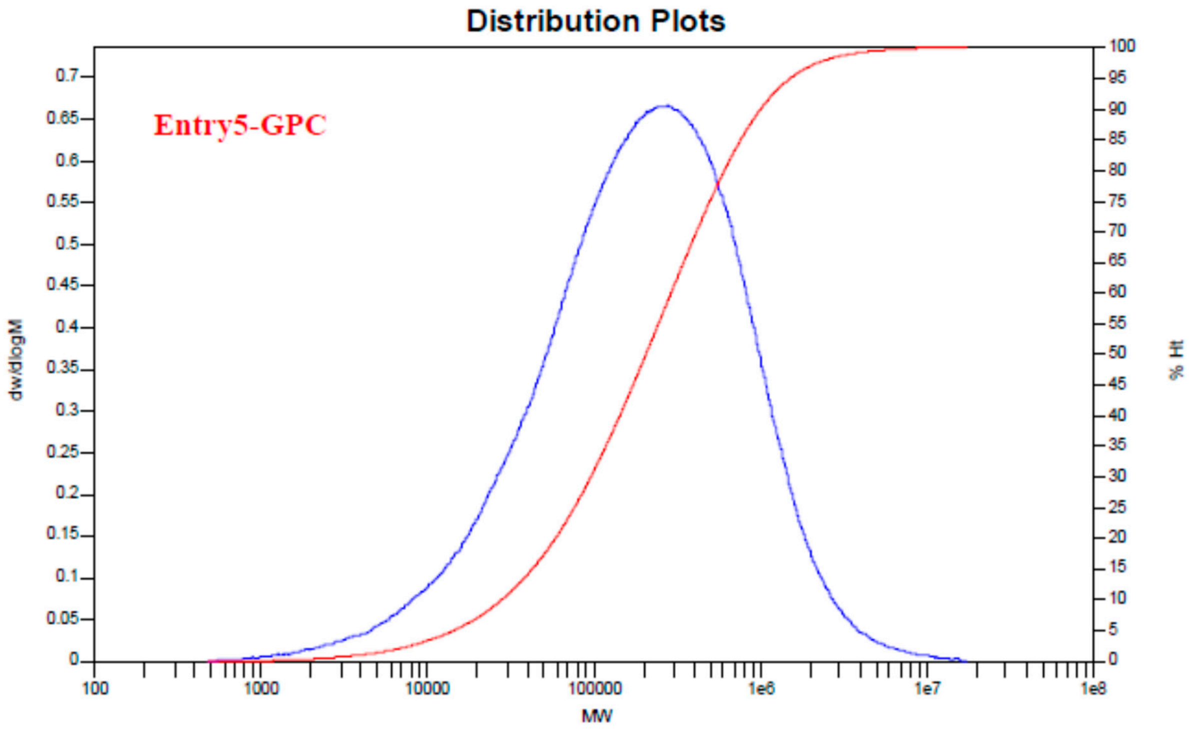

Figure S21. GPC of Entry 5 PP powder.

MW Averages

Mp: 276438	Mn: 48547	Mv: 307821	Mw: 382967
Mz: 1747106	Mz+1: 5928087	PD: 7.8886	

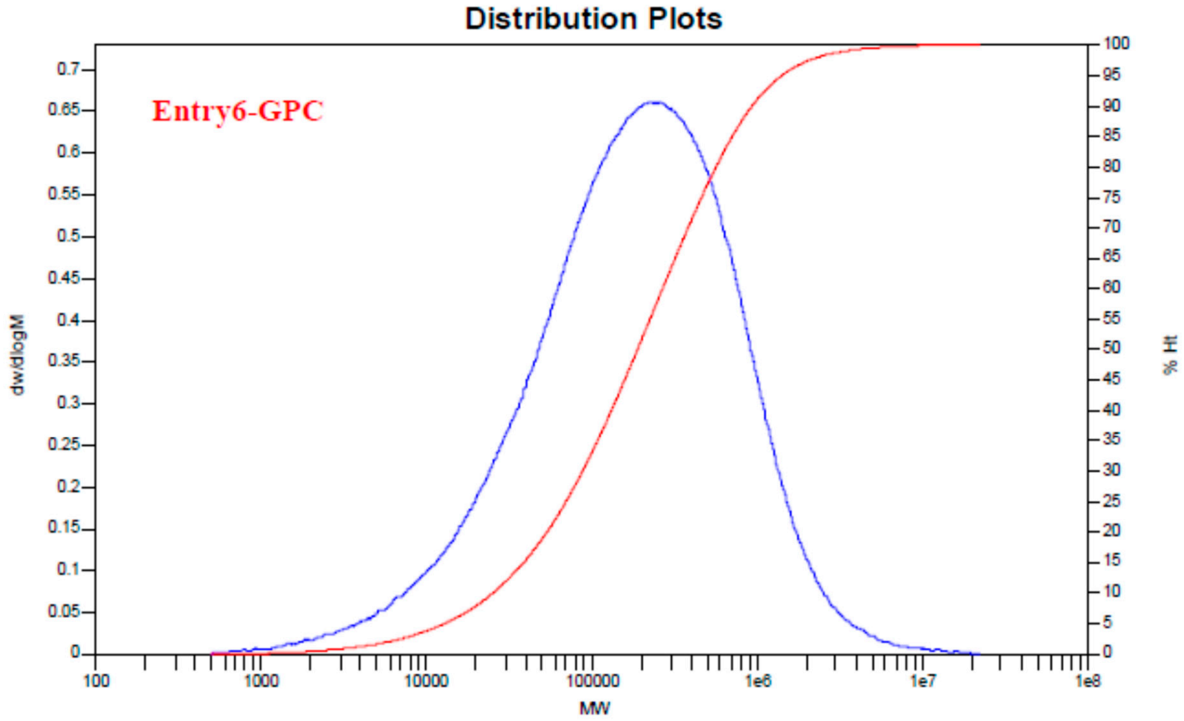

Figure S22. GPC of Entry 6 PP powder.

MW Averages

Mp: 256912	Mn: 47726	Mv: 302828	Mw: 380291
Mz: 1853427	Mz+1: 5924381	PD: 7.9682	

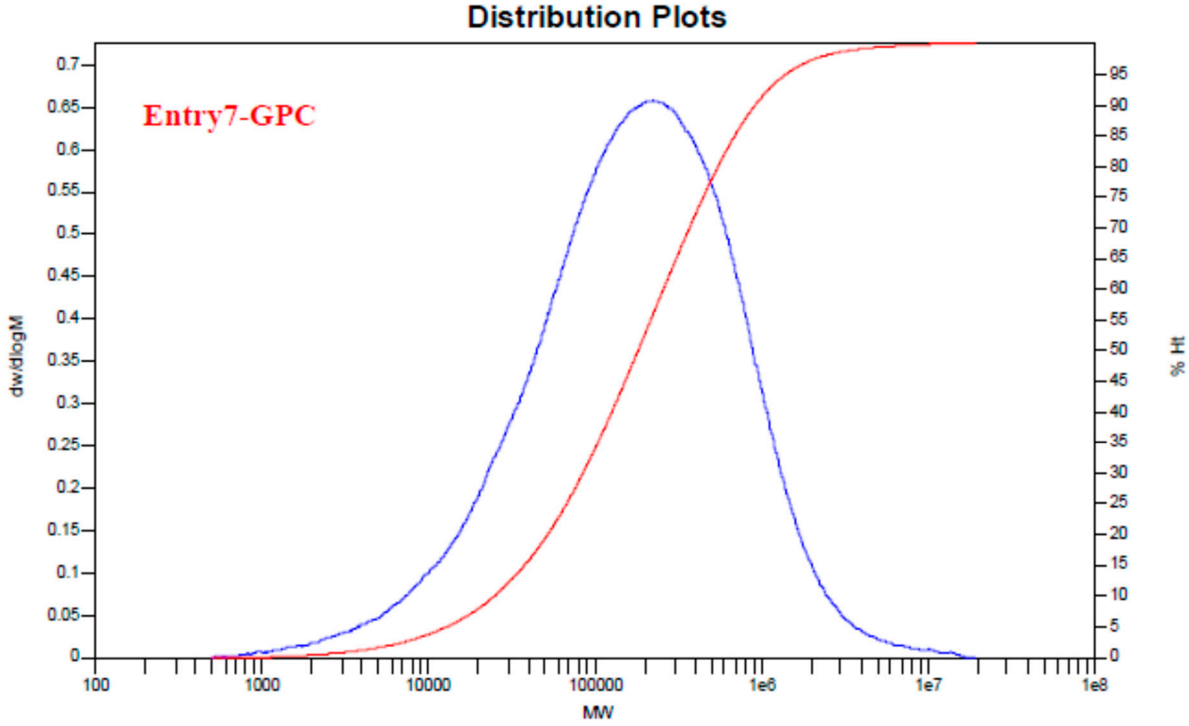

Figure S23. GPC of Entry 7 PP powder.

5. Cartestian Coordinates(Å) for the optimized and TS structures

$MgCl_2$-9

0 1

Mg	-1	12.59614600	0.74534500	-0.09606400
Mg	-1	9.44710600	-1.07279200	-0.07149000
Mg	-1	6.29807200	0.74537100	-0.04817400
Mg	-1	3.14903200	-1.07276600	-0.02359700
Mg	-1	-0.00000200	0.74539700	-0.00027900
Mg	-1	-3.14904200	-1.07274000	0.02429000
Mg	-1	-6.29807700	0.74542300	0.04761400
Mg	-1	-9.44711700	-1.07271400	0.07218600
Mg	-1	-12.59615100	0.74544900	0.09550400
Cl	0	14.42403200	1.87748900	0.29469200
Cl	0	11.45914100	-1.09234100	1.21483800
Cl	0	8.32861900	1.00518200	1.03188200
Cl	0	5.04220500	-1.08735500	1.32704300
Cl	0	1.92688800	0.90973900	1.24221600
Cl	0	-1.22854900	-1.23732800	1.28117200
Cl	0	-4.40348400	0.82184500	1.36588500
Cl	0	-7.47249800	-1.37559600	1.20182600
Cl	0	-10.84202100	0.38084200	1.63146700
Cl	0	10.84196600	0.37992200	-1.63166900
Cl	0	7.47256600	-1.37641800	-1.20106200
Cl	0	4.40353300	0.82099200	-1.36647200
Cl	0	1.22863100	-1.23834600	-1.28045700
Cl	0	-1.92682400	0.90900000	-1.24291200
Cl	0	-5.04216400	-1.08826800	-1.32635900
Cl	0	-8.32861900	1.00476700	-1.03245600
Cl	0	-11.45912900	-1.09313400	-1.21410600
Cl	0	-14.42426900	1.87715000	-0.29552100

RS-PDDB

0 1

C	3.42980900	-2.40034200	-0.77109100
C	2.75509000	-1.20197200	-0.13352600
H	2.97047200	-3.32355600	-0.41578500
H	3.34292500	-2.35356700	-1.86057300
H	4.49067300	-2.42203400	-0.50718100
C	3.33576500	0.11522000	-0.61721100
H	2.81596300	-1.27299800	0.95624000
C	2.94506800	1.35737100	0.16975800
H	4.42683500	0.02917100	-0.56498300
H	3.07812300	0.25914900	-1.67315100
H	3.05780000	1.16661400	1.24041600
C	3.74350200	2.57437200	-0.24547200

H	3.61728300	2.77354000	-1.31349200
H	3.41273100	3.45388400	0.31062400
H	4.80670500	2.41849800	-0.04575800
O	1.35703000	-1.19767000	-0.48344800
O	1.56229900	1.68256600	-0.06992900
C	0.55313100	-1.98203500	0.25294900
O	0.94627800	-2.71425000	1.12524600
C	0.66587700	1.30360500	0.85163800
O	0.94738000	0.71570400	1.86671100
C	-0.87225500	-1.82879300	-0.13051500
C	-1.82757900	-2.46256200	0.66110700
C	-1.26906100	-1.06550800	-1.22762400
C	-3.17474200	-2.32719100	0.36228600
H	-1.48861800	-3.04883700	1.50758600
C	-2.61686200	-0.93720900	-1.52713100
H	-0.51790100	-0.56883100	-1.82853300
C	-3.56929100	-1.56469900	-0.73204600
H	-3.91904100	-2.81577600	0.98193900
H	-2.92618400	-0.33392100	-2.37318000
H	-4.62369400	-1.45619800	-0.96455200
C	-0.71705200	1.68433500	0.47304000
C	-1.75604300	1.24021800	1.28861000
C	-0.99560200	2.43778100	-0.66589200
C	-3.06840400	1.54138900	0.96261400
H	-1.51036600	0.64753800	2.16186600
C	-2.31086800	2.74407900	-0.98484500
H	-0.17905500	2.77354800	-1.29313700
C	-3.34664400	2.29484900	-0.17315200
H	-3.87700800	1.18203000	1.58930900
H	-2.52974000	3.33328600	-1.86904600
H	-4.37483100	2.53020500	-0.42821300

SS-PDDB

0 1

C	-3.69992100	1.51198300	-1.71570000
C	-2.98305700	0.84844800	-0.55891100
H	-3.36828900	1.08913800	-2.66801500
H	-4.77960000	1.36509500	-1.62834200
C	-3.18965800	-0.65638800	-0.54168800
H	-3.29114800	1.30017700	0.38593700
C	-2.63917100	-1.41589500	0.66174400
H	-4.26889300	-0.84653100	-0.56990700
H	-2.77009300	-1.08944200	-1.45389600
O	-1.57562300	1.11678700	-0.72823000
O	-1.20193200	-1.31324700	0.74111000
C	-0.91565000	1.69011700	0.28525800

O	-1.42712800	2.03229600	1.32426200
C	-0.48759000	-2.03354900	-0.13169700
O	-0.97782900	-2.74733400	-0.97301100
C	0.52756300	1.85595400	-0.01615100
C	1.31475000	2.51829500	0.92369700
C	1.10730600	1.34271000	-1.17558900
C	2.67554500	2.66794800	0.70545100
H	0.83743300	2.90188800	1.81824100
C	2.47043900	1.48514400	-1.38566100
H	0.48796900	0.82177700	-1.89470900
C	3.25400600	2.14821600	-0.44787500
H	3.28792100	3.18666100	1.43530500
H	2.92493800	1.06889600	-2.27767600
H	4.32075300	2.25647700	-0.61462400
C	0.97233900	-1.84388800	0.05247500
C	1.82461700	-2.42007900	-0.88723900
C	1.49788900	-1.10748000	1.11356200
C	3.19625800	-2.25316400	-0.77311400
H	1.38786600	-2.99127400	-1.69857500
C	2.87034600	-0.94606000	1.22645800
H	0.82662400	-0.65711800	1.83398200
C	3.71912800	-1.51571800	0.28393000
H	3.85950200	-2.69862900	-1.50683500
H	3.27810800	-0.36365800	2.04486000
H	4.79239200	-1.38245700	0.37268200
H	-2.87790500	-2.47346200	0.51361400
C	-3.15461100	-0.93235500	1.99912800
H	-4.24761700	-0.95553700	2.01193300
H	-2.81830700	0.08746100	2.19922100
H	-2.78703600	-1.57803200	2.79933300
H	-3.49771300	2.58512800	-1.72404700

$TiCl_4$-$MgCl_2$-2*RS*-PDDB

0 1

Mg	-1	-12.55579300	-2.74411100	1.01023400
Mg	-1	-9.41682300	-0.92587700	0.75809100
Mg	-1	-6.27785800	-2.74394300	0.50469900
Mg	-1	-3.13888800	-0.92570900	0.25256300
Mg	-1	0.00007800	-2.74377500	-0.00082900
Mg	-1	3.13904800	-0.92554200	-0.25296000
Mg	-1	6.27801500	-2.74360800	-0.50633900
Mg	-1	9.41698500	-0.92537400	-0.75847000
Mg	-1	12.55595200	-2.74344000	-1.01184800
Cl	0	-14.35551500	-3.99341900	1.16536800
Cl	0	-11.25794300	-1.33756100	2.44760000
Cl	0	-8.27557500	-3.10638100	1.65652500

Cl	0	-4.92304500	-1.27277200	1.94045400
Cl	0	-1.84031200	-2.99691900	1.30672100
Cl	0	1.16378300	-0.89703400	1.27241500
Cl	0	4.46065600	-2.79977700	1.00966200
Cl	0	7.48969900	-0.87380800	0.66253900
Cl	0	11.12942400	-1.99701200	0.76061600
Cl	0	-11.12960900	-1.99620700	-0.76177900
Cl	0	-7.48950800	-0.87353600	-0.66287600
Cl	0	-4.46033100	-2.79900400	-1.01121200
Cl	0	-1.16361800	-0.89637200	-1.27302400
Cl	0	1.84054700	-2.99620700	-1.30836300
Cl	0	4.92318500	-1.27159900	-1.94105600
Cl	0	8.27582300	-3.10493200	-1.65848000
Cl	0	11.25851200	-1.33568900	-2.44816500
Cl	0	14.35553500	-3.99308100	-1.16622700
C	0	3.74813400	3.42547300	-1.16639900
C	0	4.93218400	2.48679900	-1.19466500
H	0	2.96458800	3.04345600	-0.51479100
H	0	4.05382800	4.41288600	-0.80997700
H	0	3.32891900	3.52708700	-2.17020200
C	0	6.05524000	3.02430500	-2.06137600
H	0	4.64254700	1.49378400	-1.54402900
C	0	7.36236500	2.25405900	-2.02879800
H	0	5.67942600	3.01894400	-3.08891100
H	0	6.26378100	4.06945400	-1.80825000
H	0	7.18525000	1.19549200	-1.81747100
C	0	8.17823700	2.42874000	-3.29109600
H	0	8.33474600	3.49011800	-3.50258100
H	0	9.14700600	1.93449300	-3.21179100
H	0	7.63938600	1.98371600	-4.13090200
O	0	5.44760800	2.33583800	0.15546700
O	0	8.10000600	2.80274800	-0.89011000
C	0	4.93613000	1.40835800	0.93623700
O	0	4.03444800	0.65762200	0.57070900
C	0	9.07137700	2.10471600	-0.36893200
O	0	9.45089600	1.03896600	-0.85642600
C	0	5.53195700	1.35234500	2.27344000
C	0	5.10276300	0.36020700	3.15510300
C	0	6.55216700	2.23071100	2.64102800
C	0	5.70285400	0.24390200	4.39779900
H	0	4.32316500	-0.32684600	2.85017600
C	0	7.15141000	2.10641000	3.88239900
H	0	6.87593800	2.99339600	1.94583300
C	0	6.72848800	1.11133300	4.75876600
H	0	5.38015100	-0.53354600	5.08054800
H	0	7.95634000	2.77752600	4.16031800

H	0	7.20466200	1.00824200	5.72812000
C	0	9.66587500	2.68841600	0.84071800
C	0	10.53790900	1.91020300	1.60296400
C	0	9.33965200	3.98471000	1.24692300
C	0	11.07087300	2.42417100	2.77459800
H	0	10.78675800	0.90311200	1.28891600
C	0	9.88517900	4.49628200	2.41281900
H	0	8.66327300	4.57866300	0.64428700
C	0	10.74718900	3.71487300	3.17796700
H	0	11.73867700	1.81496300	3.37259400
H	0	9.64014200	5.50438900	2.72770600
H	0	11.16998700	4.11647600	4.09280800
C	0	-3.74837700	3.42423300	1.16820300
C	0	-4.93226500	2.48533900	1.19605200
H	0	-2.96486900	3.04277500	0.51621600
H	0	-4.05430000	4.41182600	0.81247500
H	0	-3.32901100	3.52526100	2.17200300
C	0	-6.05530200	3.02210400	2.06326000
H	0	-4.64242400	1.49215600	1.54474400
C	0	-7.36248800	2.25199100	2.02990200
H	0	-5.67951600	3.01579600	3.09079800
H	0	-6.26379000	4.06749000	1.81107000
H	0	-7.18544200	1.19361700	1.81756500
C	0	-8.17839300	2.42554200	3.29233900
H	0	-8.33475700	3.48673300	3.50486700
H	0	-9.14724000	1.93152800	3.21251700
H	0	-7.63964600	1.97960900	4.13172800
O	0	-5.44788200	2.33515700	-0.15407700
O	0	-8.09999500	2.80184400	0.89168000
C	0	-4.93635500	1.40835200	-0.93560700
O	0	-4.03442500	0.65752900	-0.57087200
C	0	-9.07155300	2.10458100	0.36982300
O	0	-9.45139100	1.03842900	0.85617900
C	0	-5.53257900	1.35312400	-2.27267900
C	0	-5.10347500	0.36165700	-3.15513700
C	0	-6.55308200	2.23153400	-2.63935000
C	0	-5.70392800	0.24609000	-4.39772900
H	0	-4.32365600	-0.32545400	-2.85090400
C	0	-7.15268700	2.10796700	-3.88062400
H	0	-6.87678800	2.99368000	-1.94353400
C	0	-6.72983700	1.11357100	-4.75779800
H	0	-5.38129200	-0.53083400	-5.08110800
H	0	-7.95783400	2.77912100	-4.15783300
H	0	-7.20628600	1.01104400	-5.72707700
C	0	-9.66578500	2.68948000	-0.83937100
C	0	-10.53819000	1.91218300	-1.60213200

C	0	-9.33892800	3.98588500	-1.24470600
C	0	-11.07092200	2.42719300	-2.77341200
H	0	-10.78745100	0.90499500	-1.28871800
C	0	-9.88420700	4.49848700	-2.41026700
H	0	-8.66224500	4.57910200	-0.64168600
C	0	-10.74660900	3.71800600	-3.17592300
H	0	-11.73902500	1.81871400	-3.37181800
H	0	-9.63867200	5.50667800	-2.72449700
H	0	-11.16921500	4.12042600	-4.09049300
Ti	0	0.00001700	1.02356300	0.00006400
Cl	0	1.68359300	0.66238400	-1.56453600
Cl	0	1.08015700	2.43312300	1.26418400
Cl	0	-1.08016000	2.43384100	-1.26318300
Cl	0	-1.68343500	0.66155000	1.56468300

$TiCl_4$-$MgCl_2$-2*SS*-PDDB

0 1

Mg	-1	-12.55762400	-2.70311700	0.98866900
Mg	-1	-9.41824200	-0.88489900	0.74160600
Mg	-1	-6.27881000	-2.70298100	0.49416700
Mg	-1	-3.13937000	-0.88486000	0.24713000
Mg	-1	0.00002900	-2.70299500	-0.00032500
Mg	-1	3.13944900	-0.88484300	-0.24739600
Mg	-1	6.27882300	-2.70302400	-0.49483200
Mg	-1	9.41816100	-0.88472200	-0.74184100
Mg	-1	12.55765700	-2.70268500	-0.98933300
Cl	0	-14.34973700	-3.95484200	1.19605300
Cl	0	-11.31704500	-1.18046000	2.37199200
Cl	0	-8.25484300	-2.96166700	1.74905400
Cl	0	-4.89584000	-1.28870200	1.94209200
Cl	0	-1.83667400	-2.94557200	1.30766800
Cl	0	1.14857600	-0.82799700	1.26878000
Cl	0	4.45632400	-2.79203000	1.01417600
Cl	0	7.53035800	-0.95194300	0.76472000
Cl	0	11.07656100	-2.06568500	0.77014300
Cl	0	-11.07659300	-2.06560000	-0.77067000
Cl	0	-7.53049900	-0.95175500	-0.76501800
Cl	0	-4.45634600	-2.79169500	-1.01492700
Cl	0	-1.14852200	-0.82772800	-1.26913200
Cl	0	1.83662900	-2.94569500	-1.30857300
Cl	0	4.89585000	-1.28839700	-1.94245000
Cl	0	8.25487500	-2.96138300	-1.74975400
Cl	0	11.31692700	-1.17985300	-2.37232300
Cl	0	14.35018600	-3.95385000	-1.19668100
O	0	9.22652100	1.08285400	-0.90670900
C	0	8.99703500	2.18168400	-0.40029100

O	0	8.26495200	3.09143100	-0.98539800
C	0	9.52463200	2.59848100	0.90909500
C	0	7.59768200	2.84632300	-2.26513900
C	0	10.13510200	1.65382800	1.73406900
C	0	9.40229500	3.92381800	1.33670400
C	0	8.58066800	2.51464700	-3.37043900
C	0	6.47362400	1.83547200	-2.12505200
H	0	7.17654000	3.83426000	-2.46074300
C	0	10.61419100	2.02849600	2.97890300
H	0	10.22772800	0.62113700	1.42251000
C	0	9.89267900	4.29554400	2.57772700
H	0	8.92752500	4.65091700	0.68972000
H	0	8.95150000	1.49250800	-3.30817300
H	0	8.07612400	2.64221500	-4.33147400
H	0	9.43081500	3.20026200	-3.34555600
C	0	5.30095700	2.19438500	-1.22505800
H	0	6.05981800	1.68802400	-3.12863900
H	0	6.85753300	0.85977000	-1.82266100
C	0	10.49729700	3.34810100	3.39937400
H	0	11.07722300	1.28655500	3.61882400
H	0	9.80643100	5.32536400	2.90575800
O	0	5.72274700	2.08049400	0.16298300
H	0	4.52904900	1.44702800	-1.39655000
C	0	4.71797400	3.57791800	-1.40249800
H	0	10.87938400	3.64123000	4.37154500
C	0	5.06230400	1.29683500	0.98644000
H	0	4.44077200	3.73525700	-2.44822000
H	0	5.42747900	4.35296100	-1.10253200
H	0	3.81540500	3.68295800	-0.79646700
O	0	4.10715600	0.60759400	0.63674800
C	0	5.54863200	1.32692800	2.36896500
C	0	5.06165500	0.37336700	3.26352700
C	0	6.49425800	2.26604900	2.78182200
C	0	5.53128700	0.35510700	4.56620300
H	0	4.34171800	-0.36039400	2.92241700
C	0	6.95296400	2.24728600	4.08823000
H	0	6.86376200	3.00039100	2.07833500
C	0	6.47523400	1.29001900	4.97793800
H	0	5.16626500	-0.39380500	5.25954600
H	0	7.68868100	2.97527500	4.40964000
H	0	6.84235700	1.27129100	5.99871900
O	0	-9.22656200	1.08267200	0.90687900
C	0	-8.99701500	2.18162100	0.40073500
O	0	-8.26495800	3.09118400	0.98615600
C	0	-9.52464200	2.59886200	-0.90849500
C	0	-7.59774000	2.84569100	2.26586100

C	0	-10.13531400	1.65455100	-1.73371400
C	0	-9.40215100	3.92431200	-1.33571200
C	0	-8.58076800	2.51391600	3.37109700
C	0	-6.47381600	1.83472700	2.12554200
H	0	-7.17646400	3.83352600	2.46169300
C	0	-10.61445200	2.02967000	-2.97839300
H	0	-10.22805600	0.62177400	-1.42248000
C	0	-9.89259100	4.29649200	-2.57657600
H	0	-8.92719500	4.65113900	-0.68855500
H	0	-8.95174900	1.49184700	3.30862100
H	0	-8.07620400	2.64120400	4.33216100
H	0	-9.43081300	3.19966000	3.34636500
C	0	-5.30102400	2.19376800	1.22575400
H	0	-6.06010300	1.68693000	3.12911700
H	0	-6.85782900	0.85916000	1.82283600
C	0	-10.49742400	3.34939200	-3.39846000
H	0	-11.07763600	1.28799200	-3.61850600
H	0	-9.80622000	5.32640100	-2.90429900
O	0	-5.72274200	2.08049900	-0.16235700
H	0	-4.52924500	1.44621100	1.39698900
C	0	-4.71782600	3.57713500	1.40375500
H	0	-10.87957600	3.64287900	-4.37049500
C	0	-5.06228500	1.29713500	-0.98607600
H	0	-4.44068400	3.73403300	2.44955800
H	0	-5.42718100	4.35241000	1.10402600
H	0	-3.81519300	3.68225600	0.79783300
O	0	-4.10713200	0.60780800	-0.63657000
C	0	-5.54871200	1.32759700	-2.36856500
C	0	-5.06181000	0.37426600	-3.26341800
C	0	-6.49439000	2.26681300	-2.78108600
C	0	-5.53153000	0.35636200	-4.56606500
H	0	-4.34188700	-0.35961800	-2.92254400
C	0	-6.95318000	2.24841100	-4.08747300
H	0	-6.86388600	3.00091300	-2.07734100
C	0	-6.47549300	1.29140100	-4.97748100
H	0	-5.16657700	-0.39237500	-5.25963100
H	0	-7.68892600	2.97647200	-4.40865100
H	0	-6.84267300	1.27297200	-5.99824600
Ti	0	0.00007500	1.11523200	0.00003000
Cl	0	-1.71577200	0.76060300	1.54290000
Cl	0	-1.10375000	2.51595900	-1.24796200
Cl	0	1.10394600	2.51561500	1.24841500
Cl	0	1.71596500	0.76095000	-1.54283200

$TiCl_2Et$-$MgCl_2$-2*RS*-PDDB

0 2

Mg	-1	12.56939700	-2.56746700	-1.20636900
Mg	-1	9.42589500	-0.77084000	-0.86990000
Mg	-1	6.29595700	-2.60841200	-0.64908500
Mg	-1	3.15234900	-0.81159100	-0.31271900
Mg	-1	0.02260800	-2.64997900	-0.09178000
Mg	-1	-3.12089100	-0.85259900	0.24457700
Mg	-1	-6.25091700	-2.69044800	0.46545500
Mg	-1	-9.39432300	-0.89391800	0.80180200
Mg	-1	-12.52432200	-2.73158000	1.02271900
Cl	0	14.37335000	-3.79592500	-1.45842900
Cl	0	11.28364600	-1.05708500	-2.55379600
Cl	0	8.27171100	-2.86487600	-1.89321900
Cl	0	4.97664700	-1.03235100	-1.97063000
Cl	0	1.86292000	-2.86661100	-1.39661200
Cl	0	-1.21609500	-0.85298100	-1.41551000
Cl	0	-4.45969400	-2.71845700	-1.06821300
Cl	0	-7.49148600	-0.79192800	-0.64551300
Cl	0	-11.12084900	-1.92904700	-0.73883700
Cl	0	11.14325400	-1.94592800	0.59841900
Cl	0	7.56690300	-0.86842600	0.64328500
Cl	0	4.49564200	-2.80760300	0.85177300
Cl	0	1.20842500	-0.81565800	1.27285000
Cl	0	-1.80810700	-2.96386200	1.20611300
Cl	0	-4.92079300	-1.25148300	1.91862600
Cl	0	-8.24760600	-3.08946800	1.61712800
Cl	0	-11.22411100	-1.35661000	2.49019500
Cl	0	-14.31979100	-3.98957700	1.16390700
C	0	-3.76754300	3.43168100	1.14728100
C	0	-4.94607000	2.48725900	1.19370300
H	0	-2.97450000	3.01872300	0.52769200
H	0	-4.07438300	4.40522700	0.75472300
H	0	-3.36070900	3.56609000	2.15214900
C	0	-6.04875500	3.01425100	2.09224700
H	0	-4.63912000	1.49443600	1.52767400
C	0	-7.36005600	2.25188300	2.06909100
H	0	-5.65316100	2.98456100	3.11184000
H	0	-6.25690300	4.06558000	1.86511200
H	0	-7.19046000	1.19709300	1.83495700
C	0	-8.15204100	2.40694300	3.34898000
H	0	-8.29825800	3.46489800	3.58381500
H	0	-9.12520600	1.92020600	3.27755900
H	0	-7.60093400	1.94222800	4.16996400
O	0	-5.49108000	2.34787100	-0.14586600
O	0	-8.11390900	2.82502800	0.95361000
C	0	-4.97674600	1.44802900	-0.95811900
O	0	-4.04985500	0.70967500	-0.63391000

C	0	-9.09738600	2.13996400	0.43743500
O	0	-9.47538500	1.06903600	0.91406200
C	0	-5.59680700	1.41594100	-2.28606900
C	0	-5.18600900	0.43458700	-3.18860000
C	0	-6.61860200	2.30429400	-2.62418500
C	0	-5.80479500	0.34097400	-4.42420200
H	0	-4.41012000	-0.26472500	-2.90156600
C	0	-7.23600500	2.20274000	-3.85882400
H	0	-6.93024000	3.05598100	-1.91153600
C	0	-6.83037800	1.21980800	-4.75692000
H	0	-5.49835900	-0.42918500	-5.12257600
H	0	-8.04259300	2.88097500	-4.11381300
H	0	-7.32152100	1.13390100	-5.72046700
C	0	-9.70790700	2.74413200	-0.75403000
C	0	-10.59576400	1.97995700	-1.51219900
C	0	-9.38158500	4.04406400	-1.14815800
C	0	-11.14455500	2.51203500	-2.66842200
H	0	-10.84341800	0.96994000	-1.20653600
C	0	-9.94314600	4.57368800	-2.29829600
H	0	-8.69312600	4.62672300	-0.54813200
C	0	-10.82103800	3.80643600	-3.05984200
H	0	-11.82467500	1.91395300	-3.26377300
H	0	-9.69882900	5.58496500	-2.60354600
H	0	-11.25658000	4.22221300	-3.96230300
C	0	3.70814600	3.51254700	-0.56081200
C	0	4.90210700	2.61793000	-0.79946200
H	0	2.98693600	3.01149100	0.08443100
H	0	4.02031500	4.45047200	-0.09335000
H	0	3.21147000	3.73266600	-1.50858100
C	0	5.95885900	3.28222000	-1.65971900
H	0	4.59700500	1.67630300	-1.25913000
C	0	7.27180100	2.53029200	-1.78407500
H	0	5.52490600	3.38802200	-2.65843700
H	0	6.17145300	4.29366000	-1.29691400
H	0	7.11155400	1.45315500	-1.68059700
C	0	8.02081700	2.84849400	-3.05910300
H	0	8.16584600	3.92752300	-3.16123400
H	0	8.99269500	2.35433600	-3.08038800
H	0	7.44143700	2.49297900	-3.91446400
O	0	5.50689400	2.30315100	0.48118900
O	0	8.06467100	2.96086000	-0.63235300
C	0	5.00850200	1.30524300	1.18253700
O	0	4.07724300	0.61338800	0.77794600
C	0	9.04815600	2.20770100	-0.22265500
O	0	9.40147900	1.19858100	-0.83471600
C	0	5.65317600	1.10690500	2.48298700

C	0	5.28039800	0.00377200	3.25109900
C	0	6.66502200	1.96344000	2.91983400
C	0	5.92934400	-0.24441900	4.44924800
H	0	4.51277600	-0.66729400	2.88562300
C	0	7.31154900	1.70794200	4.11654200
H	0	6.94684300	2.81273200	2.31185800
C	0	6.94563400	0.60242400	4.87883700
H	0	5.65315700	-1.10924200	5.04136000
H	0	8.10920200	2.36353000	4.44754000
H	0	7.45994200	0.39636200	5.81161200
C	0	9.68706000	2.66132700	1.02014300
C	0	10.58053400	1.80882600	1.66944800
C	0	9.38197500	3.91026600	1.56735600
C	0	11.15624600	2.20058100	2.86775000
H	0	10.81473400	0.83751700	1.24995600
C	0	9.97090400	4.30090500	2.75854000
H	0	8.68797000	4.56322400	1.05230100
C	0	10.85485400	3.44475600	3.41028600
H	0	11.84009000	1.53170900	3.37731800
H	0	9.74241000	5.27265100	3.18163100
H	0	11.31182500	3.75104200	4.34534700
Ti	0	-0.01078300	0.88603200	-0.06341900
Cl	0	-1.77662800	0.68771700	1.58021300
Cl	0	1.82810400	0.80173200	-1.62006800
C	0	-0.05862400	2.93695200	-0.04547100
C	0	-0.88784500	2.80359600	-1.29915400
H	0	0.89358400	3.44026500	-0.19559100
H	0	-0.59600000	3.34249000	0.81184500
H	0	-1.82661500	3.36057600	-1.27657700
H	0	-0.33626500	3.04956100	-2.20678500
H	0	-1.24955900	1.75014600	-1.47961500

$TiCl_2Et$-$MgCl_2$-2*SS*-PDDB
0 2

Mg	-1	-12.56977000	-2.60995700	1.03381600
Mg	-1	-9.42789000	-0.80149700	0.74922500
Mg	-1	-6.29063000	-2.62847400	0.54437400
Mg	-1	-3.14860800	-0.82005300	0.25983800
Mg	-1	-0.01142600	-2.64725300	0.05495100
Mg	-1	3.13049100	-0.83855900	-0.22960300
Mg	-1	6.26781100	-2.66592600	-0.43447800
Mg	-1	9.40963500	-0.85721200	-0.71902700
Mg	-1	12.54697200	-2.68411500	-0.92390100
Cl	0	-14.36111400	-3.85773100	1.27573700
Cl	0	-11.33333400	-1.05421600	2.38412300
Cl	0	-8.27804900	-2.85037300	1.80246100

Cl	0	-4.92431800	-1.21013800	1.96102900
Cl	0	-1.85589000	-2.86663600	1.34089300
Cl	0	1.18782600	-0.79091200	1.37552900
Cl	0	4.45751600	-2.72581500	1.08726200
Cl	0	7.52970400	-0.88461800	0.78969500
Cl	0	11.08104500	-1.98164500	0.82326200
Cl	0	-11.09781600	-2.00426900	-0.74099400
Cl	0	-7.55674400	-0.92194500	-0.76855000
Cl	0	-4.47893700	-2.78306000	-0.96495600
Cl	0	-1.20271400	-0.81031800	-1.33804500
Cl	0	1.82743400	-2.93583300	-1.22507200
Cl	0	4.90323500	-1.32015600	-1.91704200
Cl	0	8.26005200	-2.96165000	-1.66354000
Cl	0	11.30844900	-1.19865400	-2.34806700
Cl	0	14.33466400	-3.94750400	-1.10215500
O	0	9.23400400	1.10301200	-0.93739200
C	0	8.99861400	2.22303200	-0.48443600
O	0	8.26380900	3.09990700	-1.11475100
C	0	9.52289300	2.70272400	0.80433600
C	0	7.59584800	2.78035400	-2.37810400
C	0	10.13275300	1.79874500	1.67424100
C	0	9.39946600	4.04703000	1.16678100
C	0	8.57891900	2.40397700	-3.46881900
C	0	6.48373800	1.76623200	-2.18038600
H	0	7.16268500	3.75190800	-2.62340000
C	0	10.61094100	2.23400800	2.89968200
H	0	10.22420300	0.75226000	1.41158700
C	0	9.88943100	4.47894800	2.38828300
H	0	8.92545200	4.74145400	0.48430600
H	0	8.96307600	1.39115200	-3.35637200
H	0	8.06905600	2.47524700	-4.43289800
H	0	9.42048100	3.10043600	-3.48337100
C	0	5.32140200	2.15471900	-1.28002800
H	0	6.05668000	1.57284300	-3.17044700
H	0	6.87833500	0.80654500	-1.84333300
C	0	10.49406200	3.57261400	3.25512400
H	0	11.07380500	1.52447700	3.57548100
H	0	9.80379200	5.52365400	2.66559900
O	0	5.75889500	2.09720200	0.10762400
H	0	4.54564100	1.40422300	-1.41398500
C	0	4.73932200	3.53116100	-1.50814600
H	0	10.87636500	3.91286400	4.21176200
C	0	5.11125300	1.34466600	0.97085900
H	0	4.45734300	3.64718000	-2.55787800
H	0	5.44959000	4.31723800	-1.24028700
H	0	3.83718000	3.65957200	-0.90633000

O	0	4.15330800	0.63840900	0.66938100
C	0	5.61383200	1.44069200	2.34646600
C	0	5.17795500	0.49566700	3.27601400
C	0	6.52138700	2.43278900	2.71804000
C	0	5.65964700	0.54116900	4.57375900
H	0	4.49332400	-0.28348500	2.96281300
C	0	6.99063000	2.47838400	4.02045100
H	0	6.85414400	3.15704100	1.98611400
C	0	6.56324200	1.53128000	4.94571300
H	0	5.33733300	-0.20194600	5.29405300
H	0	7.69626200	3.24803000	4.31067300
H	0	6.93965600	1.56255600	5.96281600
O	0	-9.24338400	1.16673000	0.86090700
C	0	-9.00944600	2.26012000	0.34589800
O	0	-8.27944500	3.17301400	0.92878700
C	0	-9.52961900	2.66334600	-0.97014800
C	0	-7.60892500	2.92265600	2.20626800
C	0	-10.13913400	1.70936800	-1.78511900
C	0	-9.40207500	3.98277900	-1.41358900
C	0	-8.58852500	2.60104300	3.31747400
C	0	-6.49346700	1.90332100	2.05947400
H	0	-7.17873300	3.90717300	2.39941600
C	0	-10.61297400	2.06969200	-3.03613100
H	0	-10.23438600	0.68122100	-1.45943700
C	0	-9.88772900	4.34014200	-2.66069200
H	0	-8.92777000	4.71644900	-0.77364300
H	0	-8.96892800	1.58225600	3.25969500
H	0	-8.07714200	2.72545900	4.27534100
H	0	-9.43271500	3.29412500	3.29646000
C	0	-5.33903600	2.24564200	1.13010500
H	0	-6.06015700	1.76742800	3.05638200
H	0	-6.88626800	0.92501500	1.77857400
C	0	-10.49188500	3.38377900	-3.47231200
H	0	-11.07527400	1.32097400	-3.66863000
H	0	-9.79848900	5.36563400	-3.00134400
O	0	-5.78318600	2.09949800	-0.24817900
H	0	-4.55596600	1.51103800	1.30537100
C	0	-4.76821200	3.63869800	1.26797700
H	0	-10.87030300	3.66568500	-4.44924200
C	0	-5.14370700	1.28591000	-1.06171600
H	0	-4.47508600	3.82031600	2.30532100
H	0	-5.49036900	4.40006300	0.96307000
H	0	-3.87682200	3.73994500	0.64524200
O	0	-4.19329600	0.59144900	-0.71458300
C	0	-5.64798400	1.29694700	-2.43985600
C	0	-5.23100000	0.28293200	-3.30281900

C	0	-6.53717100	2.27827800	-2.87798300
C	0	-5.71290900	0.24920900	-4.60072100
H	0	-4.56141600	-0.48619100	-2.93657200
C	0	-7.00624600	2.24438400	-4.18089500
H	0	-6.85543500	3.05670900	-2.19690300
C	0	-6.59749700	1.22892000	-5.03971400
H	0	-5.40497600	-0.54696000	-5.26872000
H	0	-7.69676600	3.00624500	-4.52344800
H	0	-6.97349300	1.19874100	-6.05701100
Ti	0	-0.01406200	0.91923800	-0.03141200
Cl	0	-1.86884200	0.86387300	1.51658900
Cl	0	1.85786900	0.76921400	-1.57227100
C	0	-0.04318100	2.96110100	-0.17053800
C	0	0.90578100	2.92999000	0.99988000
H	0	-1.01376300	3.40514500	0.04573200
H	0	0.38707500	3.34109200	-1.09610600
H	0	0.49085100	3.36147100	1.91146200
H	0	1.18192500	1.88075200	1.32246200
H	0	1.88171200	3.36836900	0.78584000

$TiCl_2Et$-$MgCl_2$-2*RS*-PDDB-propene
0 2

Mg	-1	12.58589200	-2.71905700	-1.02289500
Mg	-1	9.44114000	-0.91654200	-0.73163600
Mg	-1	6.30756000	-2.74949400	-0.52312800
Mg	-1	3.16291700	-0.94705800	-0.23186000
Mg	-1	0.02540800	-2.77331300	-0.02294300
Mg	-1	-3.11548800	-0.96427400	0.26823300
Mg	-1	-6.25295200	-2.79060500	0.47713000
Mg	-1	-9.39381400	-0.98152500	0.76824300
Mg	-1	-12.53128200	-2.80785600	0.97720700
Cl	0	14.39760800	-3.94728100	-1.21496000
Cl	0	11.27693400	-1.29817000	-2.43870000
Cl	0	8.33195900	-3.10516300	-1.63381800
Cl	0	4.97258400	-1.30607600	-1.94238900
Cl	0	1.89585700	-3.05883600	-1.27164300
Cl	0	-1.21341800	-1.00063900	-1.36703800
Cl	0	-4.46619100	-2.80687500	-1.06062500
Cl	0	-7.46505000	-0.85665900	-0.62272300
Cl	0	-11.11265400	-2.01684700	-0.77756600
Cl	0	11.16840400	-2.00829000	0.76622600
Cl	0	7.50999100	-0.85707600	0.65985900
Cl	0	4.51926900	-2.81071100	1.02552300
Cl	0	1.20466900	-0.91534000	1.30200600
Cl	0	-1.81610400	-3.07854300	1.26850000
Cl	0	-4.91600400	-1.40718000	1.96271200

Cl	0	-8.27457000	-3.19088600	1.58108900
Cl	0	-11.23205700	-1.44348400	2.45554900
Cl	0	-14.33783600	-4.05162600	1.10950100
C	0	-3.90899600	3.27609800	1.42675300
C	0	-5.08057300	2.32303300	1.39540100
H	0	-3.08981100	2.89561100	0.82361800
H	0	-4.21148000	4.26236000	1.06369900
H	0	-3.54020800	3.37248200	2.45070000
C	0	-6.20560400	2.81419800	2.28790100
H	0	-4.78166000	1.31532700	1.69072800
C	0	-7.52720600	2.08231400	2.17192600
H	0	-5.84584400	2.71419300	3.31622900
H	0	-6.38734600	3.88131400	2.11942100
H	0	-7.36093300	1.03821400	1.89292000
C	0	-8.37568400	2.18481800	3.42022800
H	0	-8.51858300	3.23143600	3.70259900
H	0	-9.35127200	1.71812000	3.27825900
H	0	-7.87108300	1.66964500	4.24100100
O	0	-5.58985400	2.24228400	0.03483100
O	0	-8.21972700	2.72472600	1.05505200
C	0	-5.06175200	1.37802500	-0.80558000
O	0	-4.15404000	0.61010700	-0.49730900
C	0	-9.17021500	2.07018100	0.44546500
O	0	-9.57022700	0.97370100	0.83548300
C	0	-5.62873100	1.43462100	-2.15786200
C	0	-5.23339800	0.46906400	-3.08468400
C	0	-6.57746800	2.39806100	-2.50133700
C	0	-5.79366000	0.46870500	-4.35143100
H	0	-4.52329200	-0.29512700	-2.79106000
C	0	-7.13612600	2.39010600	-3.76844800
H	0	-6.87792200	3.13641500	-1.76989900
C	0	-6.74508200	1.42517100	-4.69165200
H	0	-5.50222100	-0.28997500	-5.06863400
H	0	-7.88539400	3.12862500	-4.03030400
H	0	-7.19029600	1.41340500	-5.68093700
C	0	-9.71708800	2.73746800	-0.74252300
C	0	-10.53865500	2.00265900	-1.59822900
C	0	-9.39632300	4.06464200	-1.03647600
C	0	-11.02867700	2.59390900	-2.75206700
H	0	-10.77670700	0.97102500	-1.36532700
C	0	-9.90091400	4.65294700	-2.18464600
H	0	-8.75854200	4.62211200	-0.36110000
C	0	-10.71350200	3.91667500	-3.04312200
H	0	-11.65745800	2.02100500	-3.42374100
H	0	-9.66348400	5.68602900	-2.41266200
H	0	-11.10452500	4.37906800	-3.94331600

C	0	3.87795500	3.34854000	-1.14038800
C	0	5.06325100	2.41212200	-1.15597600
H	0	3.08318400	2.94874500	-0.51529300
H	0	4.17467400	4.33136300	-0.76340000
H	0	3.48401100	3.46135600	-2.15346600
C	0	6.18918600	2.96049700	-2.01234700
H	0	4.77739700	1.41906500	-1.50863100
C	0	7.50952700	2.21792600	-1.94901700
H	0	5.82957600	2.93334700	-3.04534400
H	0	6.37405200	4.01282200	-1.77022200
H	0	7.34281900	1.15873900	-1.73313800
C	0	8.34771500	2.39768100	-3.19585900
H	0	8.48791700	3.46012500	-3.41340800
H	0	9.32451100	1.92374700	-3.09292100
H	0	7.83522600	1.93487900	-4.04255400
O	0	5.56379000	2.26350700	0.19924300
O	0	8.21200200	2.78821300	-0.79953800
C	0	5.02161700	1.35702800	0.98630600
O	0	4.10562500	0.62306300	0.62448400
C	0	9.18188600	2.10697100	-0.25272400
O	0	9.58873800	1.04477000	-0.72364700
C	0	5.59834000	1.31366600	2.33364600
C	0	5.16056700	0.32476800	3.21475600
C	0	6.60513400	2.20239300	2.71285600
C	0	5.73816600	0.22321000	4.46959500
H	0	4.39539000	-0.37348700	2.89829400
C	0	7.18079700	2.09372900	3.96698400
H	0	6.93746000	2.96092800	2.01703800
C	0	6.74882100	1.10267200	4.84354600
H	0	5.41009000	-0.55269900	5.15159700
H	0	7.97475200	2.77376000	4.25470900
H	0	7.20688600	1.01150200	5.82279500
C	0	9.74240300	2.70118700	0.96734100
C	0	10.59661500	1.92620300	1.75281900
C	0	9.40197500	3.99748200	1.36070600
C	0	11.09957300	2.44540400	2.93537600
H	0	10.85051400	0.91845000	1.44451400
C	0	9.91787600	4.51388300	2.53801700
H	0	8.73871500	4.58737400	0.73961700
C	0	10.76321800	3.73686400	3.32604400
H	0	11.75373000	1.84013400	3.55219500
H	0	9.66306100	5.52229700	2.84411400
H	0	11.16271900	4.14262700	4.24951500
Ti	0	0.08865300	0.89032400	-0.02911400
Cl	0	-1.75599300	0.61386600	1.54375500
Cl	0	1.86901200	0.59271100	-1.63247400

C	0	0.89896600	2.53026500	0.94347600
C	0	0.23016000	3.18676900	2.14065900
H	0	1.12114800	3.26661900	0.15763400
H	0	1.86651100	2.08507100	1.23112300
C	0	-0.60420600	2.41634500	-2.22695600
H	0	0.84530100	3.99358100	2.55645000
H	0	-0.73913200	3.62145500	1.87495000
H	0	0.04464200	2.46423600	2.93850200
C	0	-1.45464000	2.49210700	-1.18748300
H	0	0.30243200	3.01947200	-2.20355600
C	0	-0.84607900	1.64189500	-3.47331900
H	0	-1.28234500	3.19311500	-0.37693300
H	0	-2.39501300	1.95220400	-1.19712100
H	0	-0.01958400	0.95736300	-3.68118500
H	0	-1.77572800	1.07385800	-3.42572600
H	0	-0.90420300	2.34231000	-4.31424100

$TiCl_2Et$-$MgCl_2$-2*SS*-PDDB-propene

0 2

Mg	-1	12.57278600	-2.68110700	-1.04566300
Mg	-1	9.43287600	-0.87040800	-0.75083900
Mg	-1	6.29531200	-2.69521400	-0.53420000
Mg	-1	3.15542300	-0.88462100	-0.23949500
Mg	-1	0.01773000	-2.70941100	-0.02289000
Mg	-1	-3.12170000	-0.89882100	0.27199600
Mg	-1	-6.25967600	-2.72354200	0.48859000
Mg	-1	-9.39899600	-0.91299700	0.78329100
Mg	-1	-12.53720100	-2.73787700	1.00000600
Cl	0	14.36390400	-3.92830700	-1.29397800
Cl	0	11.32982500	-1.12997200	-2.39507500
Cl	0	8.28223800	-2.92451700	-1.79243800
Cl	0	4.93419900	-1.27284500	-1.94751900
Cl	0	1.87184300	-2.96140700	-1.30070600
Cl	0	-1.20234700	-0.90560700	-1.33884000
Cl	0	-4.46283100	-2.81464900	-1.02910400
Cl	0	-7.54176500	-0.96943500	-0.75241900
Cl	0	-11.07709400	-2.04819600	-0.75328400
Cl	0	11.11319800	-2.07001200	0.73567700
Cl	0	7.56999500	-0.98303800	0.77212100
Cl	0	4.49740300	-2.81938100	0.99375200
Cl	0	1.18385700	-0.84812100	1.31123300
Cl	0	-1.81054800	-2.99589100	1.29055600
Cl	0	-4.89678800	-1.34603500	1.95430400
Cl	0	-8.24166900	-2.99166000	1.74931400
Cl	0	-11.29891200	-1.24138000	2.41343700
Cl	0	-14.32521900	-3.99953600	1.18882400

O	0	-9.23732500	1.05163000	0.96598000
C	0	-9.01259500	2.16605400	0.49450800
O	0	-8.28434400	3.05879800	1.11028400
C	0	-9.54172600	2.61981900	-0.80134700
C	0	-7.60840400	2.75996500	2.37452800
C	0	-10.18001200	1.70396000	-1.63794900
C	0	-9.39196300	3.94965900	-1.20519500
C	0	-6.49354400	1.74765700	2.18360100
H	0	-7.17733200	3.73642200	2.60343800
C	0	-10.65874700	2.11268700	-2.87231900
H	0	-10.29729600	0.66917800	-1.34109000
C	0	-9.88087700	4.35493900	-2.43623900
H	0	-8.89653900	4.65320300	-0.54777200
C	0	-5.35231000	2.11985100	1.25048500
H	0	-6.04627100	1.58420800	3.17013500
H	0	-6.88751100	0.77674600	1.88023600
C	0	-10.51271400	3.43640600	-3.27033700
H	0	-11.14481100	1.39400000	-3.52165000
H	0	-9.77314900	5.38822900	-2.74660200
O	0	-5.81003400	2.00078200	-0.12866500
H	0	-4.56195400	1.38727700	1.39900800
H	0	-10.89449200	3.75581300	-4.23435200
C	0	-5.15127800	1.23421300	-0.96939000
O	0	-4.17727100	0.55786700	-0.64640700
C	0	-5.64046000	1.28391000	-2.35160100
C	0	-5.16376300	0.33109700	-3.25347000
C	0	-6.56505800	2.24502600	-2.75958800
C	0	-5.62103300	0.33963600	-4.56089800
H	0	-4.46922300	-0.42698200	-2.91065500
C	0	-7.01115900	2.25241700	-4.07095600
H	0	-6.92989300	2.97485900	-2.04897600
C	0	-6.54210000	1.29914700	-4.96925000
H	0	-5.26761600	-0.40994700	-5.25961500
H	0	-7.73161900	2.99711100	-4.38891300
H	0	-6.90028700	1.30051600	-5.99336600
O	0	9.26588200	1.10283500	-0.85470700
C	0	9.03911500	2.18841400	-0.32015800
O	0	8.30036200	3.11011600	-0.87834000
C	0	9.57853200	2.57534200	0.99326800
C	0	7.62387200	2.88756000	-2.15732500
C	0	10.22479200	1.61946000	1.77712400
C	0	9.43226000	3.88310200	1.46505100
C	0	6.51451300	1.85887400	-2.03274100
H	0	7.18780500	3.87434400	-2.32450600
C	0	10.71455800	1.96579300	3.02614300
H	0	10.34013800	0.60078800	1.42858900

C	0	9.93256100	4.22645900	2.71017900
H	0	8.93004900	4.61836600	0.84881400
C	0	5.35404900	2.17302700	-1.10212000
H	0	6.08489800	1.73915900	-3.03337000
H	0	6.91366100	0.87823400	-1.76966500
C	0	10.57208400	3.26771600	3.49145000
H	0	11.20608300	1.21540200	3.63418100
H	0	9.82686900	5.24267400	3.07315600
O	0	5.79861200	2.01747400	0.27475100
H	0	4.58551000	1.42589300	-1.28701600
H	0	10.96193700	3.53840000	4.46705600
C	0	5.13557700	1.22096800	1.08569300
O	0	4.16787800	0.55305800	0.73142900
C	0	5.63002600	1.21661700	2.46661600
C	0	5.14547400	0.24104800	3.33854800
C	0	6.57600100	2.14585600	2.89966700
C	0	5.61795000	0.19210700	4.63963000
H	0	4.42753000	-0.48601800	2.97874700
C	0	7.03783100	2.09600900	4.20427800
H	0	6.94473500	2.89595400	2.21272200
C	0	6.56213000	1.11767400	5.07183100
H	0	5.25587900	-0.57430600	5.31521300
H	0	7.77515600	2.81553800	4.54101400
H	0	6.93191600	1.07418000	6.09091600
Cl	0	-1.82031500	0.75650600	1.55047000
Cl	0	1.89347800	0.75895600	-1.56342800
Ti	0	0.08254200	1.00680100	0.03300200
C	0	1.03740100	2.57637100	0.99398800
C	0	0.42534600	3.17761700	2.25107300
H	0	2.00708400	2.10376000	1.21561300
H	0	1.24258400	3.34081100	0.23001000
H	0	0.26697300	2.41692400	3.01942600
H	0	1.06871700	3.95386900	2.68246600
H	0	-0.54974400	3.63567100	2.05455300
C	0	8.60195100	2.59807800	-3.27867600
H	0	8.98977400	1.58101200	-3.24386100
H	0	8.08729000	2.73986800	-4.23233300
H	0	9.44116600	3.29664700	-3.24416600
C	0	4.75557300	3.55608700	-1.22519100
H	0	4.45803900	3.74203700	-2.26068700
H	0	3.86315900	3.63136200	-0.59979300
H	0	5.46250500	4.32918000	-0.91386300
C	0	-4.79182200	3.51426600	1.41436900
H	0	-4.48364900	3.67108800	2.45139200
H	0	-3.91054100	3.64089600	0.78224800
H	0	-5.52542600	4.27650900	1.14063400

C	0	-8.58359200	2.39612800	3.47625300
H	0	-9.42628900	3.09124000	3.48595300
H	0	-8.96645500	1.38122200	3.37990600
H	0	-8.06817200	2.48166400	4.43618100
C	0	-1.49324000	2.66668900	-0.97561700
C	0	-0.66560200	2.58629200	-2.03478000
H	0	-1.28951800	3.33855000	-0.14714900
H	0	-2.44600800	2.14829400	-0.97286100
H	0	0.25624600	3.16594500	-2.02291600
C	0	-0.95725600	1.84252700	-3.29069700
H	0	-1.89714800	1.29252900	-3.22936100
H	0	-1.02376400	2.56153100	-4.11516200
H	0	-0.15140300	1.14581000	-3.53590700

$TiCl_2Et$-$MgCl_2$-2*RS*-PDDB-TS-*si*

0 2

Mg	-1	12.54492600	-2.78937600	-1.07127000
Mg	-1	9.40912600	-0.96711900	-0.80894500
Mg	-1	6.27051500	-2.78109000	-0.52380200
Mg	-1	3.13471400	-0.95883500	-0.26147800
Mg	-1	-0.00389700	-2.77280500	0.02366400
Mg	-1	-3.13969700	-0.95054900	0.28598900
Mg	-1	-6.27830900	-2.76451900	0.57113100
Mg	-1	-9.41410900	-0.94226300	0.83345600
Mg	-1	-12.55272000	-2.75623500	1.11859800
Cl	0	14.36183200	-4.00892800	-1.27232700
Cl	0	11.24064800	-1.36919900	-2.50261000
Cl	0	8.27895700	-3.14987700	-1.66963600
Cl	0	4.93092700	-1.35232800	-1.96467700
Cl	0	1.85551900	-3.07912400	-1.25028900
Cl	0	-1.21981900	-1.03459100	-1.31454200
Cl	0	-4.49242100	-2.86973600	-0.95234200
Cl	0	-7.52339400	-0.92086500	-0.62312200
Cl	0	-11.13621500	-2.05299400	-0.66820900
Cl	0	11.12230500	-2.08458200	0.70590100
Cl	0	7.49256100	-0.87519400	0.60064200
Cl	0	4.49828300	-2.81840400	1.03211500
Cl	0	1.18573800	-0.95586200	1.31676400
Cl	0	-1.83949300	-3.03077200	1.35283900
Cl	0	-4.95586900	-1.26133400	1.96822400
Cl	0	-8.27557200	-3.09227000	1.75395200
Cl	0	-11.25625600	-1.31782700	2.53003100
Cl	0	-14.36018400	-3.99005400	1.31622700
C	0	-3.87008400	3.37388700	0.98256800
C	0	-5.03996700	2.42449800	1.08908600
H	0	-3.08873700	2.93686000	0.36508300

H	0	-4.19025300	4.32560600	0.54926700
H	0	-3.44764300	3.55686900	1.97312000
C	0	-6.13992600	2.99330300	1.96510800
H	0	-4.72425100	1.45317000	1.47442900
C	0	-7.45513100	2.23876600	1.95931200
H	0	-5.74863000	2.99677100	2.98676400
H	0	-6.34088200	4.03688000	1.69934200
H	0	-7.28670100	1.17649200	1.76045100
C	0	-8.25741800	2.43702200	3.22652900
H	0	-8.40363400	3.50222000	3.42578200
H	0	-9.23106000	1.95003300	3.16205600
H	0	-7.71476800	1.99772900	4.06695000
O	0	-5.58730200	2.20961200	-0.23849900
O	0	-8.19535400	2.77996700	0.81947400
C	0	-5.05184100	1.28173800	-1.00599900
O	0	-4.11492400	0.57592200	-0.63904800
C	0	-9.16856300	2.07521400	0.30989200
O	0	-9.54959800	1.01889500	0.81427700
C	0	-5.65422100	1.18477100	-2.33795500
C	0	-5.22267800	0.16997300	-3.19251000
C	0	-6.67826500	2.04959400	-2.72657700
C	0	-5.82533000	0.01862600	-4.43047800
H	0	-4.44478000	-0.50980100	-2.86643600
C	0	-7.27855700	1.89088600	-3.96345100
H	0	-7.00580300	2.82765500	-2.05028300
C	0	-6.85351500	0.87378000	-4.81332700
H	0	-5.50360000	-0.77789200	-5.09139300
H	0	-8.08613500	2.55176400	-4.25803000
H	0	-7.33119500	0.74267700	-5.77856000
C	0	-9.76359000	2.63581400	-0.90974800
C	0	-10.63900200	1.84071800	-1.65055600
C	0	-9.43414200	3.92087900	-1.34743100
C	0	-11.17384000	2.32816300	-2.83265700
H	0	-10.88538300	0.84193800	-1.30896200
C	0	-9.98159200	4.40560700	-2.52386700
H	0	-8.75405600	4.52695300	-0.76113600
C	0	-10.84812800	3.60822200	-3.26737900
H	0	-11.84458800	1.70674700	-3.41458900
H	0	-9.73493400	5.40508400	-2.86401800
H	0	-11.27259400	3.98907400	-4.19030400
C	0	3.97238400	3.29529300	-1.20187100
C	0	5.15683600	2.35987100	-1.19454500
H	0	3.14721800	2.85436100	-0.64916700
H	0	4.24525900	4.25919200	-0.76281300
H	0	3.63293300	3.45591600	-2.22771800
C	0	6.29696000	2.91728600	-2.02534400

H	0	4.87725900	1.36836700	-1.55825300
C	0	7.61444800	2.17299400	-1.94654700
H	0	5.95505400	2.90010900	-3.06445800
H	0	6.47785500	3.96704700	-1.76945900
H	0	7.43793100	1.11410800	-1.73668000
C	0	8.47077400	2.35401400	-3.18105200
H	0	8.62025400	3.41678000	-3.39052900
H	0	9.44345200	1.87383900	-3.06823400
H	0	7.96684300	1.89955000	-4.03739100
O	0	5.62743500	2.20692800	0.16966500
O	0	8.30145700	2.73395500	-0.78456100
C	0	5.05221500	1.31500600	0.95277600
O	0	4.12450800	0.60229100	0.58274800
C	0	9.26328600	2.04424800	-0.23265800
O	0	9.66785100	0.98200700	-0.70450500
C	0	5.61320700	1.26658200	2.30758200
C	0	5.14503600	0.29297800	3.18991000
C	0	6.63745100	2.13344600	2.69094000
C	0	5.70872000	0.18607000	4.45094400
H	0	4.36858300	-0.39112700	2.86972600
C	0	7.19896200	2.01959600	3.95098800
H	0	6.99592300	2.87749800	1.99243200
C	0	6.73564700	1.04453200	4.82951300
H	0	5.35722600	-0.57868600	5.13391800
H	0	8.00687800	2.68197700	4.24139800
H	0	7.18260300	0.94838000	5.81340700
C	0	9.81797800	2.62816300	0.99441000
C	0	10.66368700	1.84242700	1.77856500
C	0	9.48182100	3.92339600	1.39479800
C	0	11.16276300	2.35088400	2.96746600
H	0	10.91194600	0.83587700	1.46153900
C	0	9.99418400	4.42875800	2.57840100
H	0	8.82512800	4.52129100	0.77432200
C	0	10.83123600	3.64151100	3.36528200
H	0	11.81044500	1.73819600	3.58379600
H	0	9.74323600	5.43634100	2.89036600
H	0	11.22812000	4.03868000	4.29360100
Cl	0	1.81650600	0.64554600	-1.58865700
Ti	0	-0.04930000	0.89079400	-0.03718700
Cl	0	-1.82006900	0.68962300	1.58202800
C	0	-1.15622600	2.07676000	-1.52744600
C	0	0.68467000	2.68569000	0.87253500
C	0	-0.61069400	3.18424400	-0.88660600
H	0	-2.16936100	1.76390800	-1.29481700
H	0	-0.76093400	1.76286900	-2.48891800
C	0	0.10549300	3.62874400	1.89958300

H	0	1.60073600	3.07993200	0.43493300
H	0	0.99395500	1.74429000	1.40623800
H	0	-1.22968200	3.68641500	-0.15051900
C	0	0.40141400	4.04754900	-1.57601000
H	0	0.79948200	3.77202600	2.73507300
H	0	-0.07635900	4.61807800	1.46762000
H	0	-0.83324800	3.25002600	2.31011700
H	0	0.94283400	4.70188700	-0.89063100
H	0	1.11403600	3.44292100	-2.13915600
H	0	-0.13702900	4.68264300	-2.28799300

$TiCl_2Et$-$MgCl_2$-2*RS*-PDDB-TS-*re*

0 2

Mg	-1	12.57298800	-2.78042500	-1.04849400
Mg	-1	9.43316200	-0.96301000	-0.80115200
Mg	-1	6.29732300	-2.78170200	-0.51544700
Mg	-1	3.15750100	-0.96429700	-0.26812600
Mg	-1	0.02166100	-2.78296800	0.01758900
Mg	-1	-3.11814500	-0.96554900	0.26494100
Mg	-1	-6.25401800	-2.78424100	0.55063700
Mg	-1	-9.39377900	-0.96683700	0.79796600
Mg	-1	-12.52966300	-2.78554400	1.08368200
Cl	0	14.38421200	-4.01276100	-1.21730300
Cl	0	11.26523700	-1.38914600	-2.49656800
Cl	0	8.31049500	-3.16008600	-1.64189900
Cl	0	4.96480400	-1.34612300	-1.95832700
Cl	0	1.87990700	-3.07599300	-1.26392100
Cl	0	-1.19058700	-0.99920200	-1.30941300
Cl	0	-4.48069500	-2.84532200	-1.00050200
Cl	0	-7.46684400	-0.87576000	-0.59271400
Cl	0	-11.10532500	-2.10195400	-0.70233700
Cl	0	11.15842400	-2.02991000	0.72406000
Cl	0	7.51530900	-0.86108500	0.61088400
Cl	0	4.52104300	-2.81322500	1.03385600
Cl	0	1.23058500	-0.98299000	1.32788500
Cl	0	-1.82270900	-3.07497500	1.31320600
Cl	0	-4.90819900	-1.35483400	1.98109700
Cl	0	-8.26891600	-3.14172600	1.68401000
Cl	0	-11.22544900	-1.35049200	2.49852800
Cl	0	-14.34783000	-4.00109200	1.29680200
C	0	-3.96884900	3.23636500	1.23637200
C	0	-5.17703900	2.33200400	1.21297600
H	0	-3.15322700	2.78378900	0.67860100
H	0	-4.21781700	4.21287300	0.81077000
H	0	-3.62300900	3.37105700	2.26411100
C	0	-6.31575500	2.91656300	2.02634000

H	0	-4.92936700	1.33249600	1.57830500
C	0	-7.64208800	2.18836400	1.94227800
H	0	-5.98419700	2.90613300	3.06894100
H	0	-6.47796100	3.96589900	1.75660800
H	0	-7.47447400	1.12521100	1.74750500
C	0	-8.51149500	2.39287700	3.16354900
H	0	-8.65265300	3.45952800	3.35839700
H	0	-9.48756800	1.92123600	3.04285800
H	0	-8.02414500	1.94335900	4.03197100
O	0	-5.63492600	2.19176900	-0.15796400
O	0	-8.31206800	2.74089700	0.76676600
C	0	-5.05115600	1.30531100	-0.93958700
O	0	-4.12350400	0.59509000	-0.56211000
C	0	-9.26096700	2.04212700	0.20308200
O	0	-9.66640100	0.97976800	0.67366900
C	0	-5.59251500	1.26327400	-2.30199700
C	0	-5.10800900	0.29655000	-3.18329800
C	0	-6.61090800	2.13202000	-2.69640900
C	0	-5.65053600	0.19761100	-4.45414600
H	0	-4.33493600	-0.38802200	-2.85592700
C	0	-7.15055900	2.02650800	-3.96671900
H	0	-6.98163100	2.87134100	-1.99922900
C	0	-6.67178200	1.05778100	-4.84398200
H	0	-5.28644300	-0.56181300	-5.13647300
H	0	-7.95355400	2.69061000	-4.26641700
H	0	-7.10180200	0.96837500	-5.83603100
C	0	-9.79868500	2.61458000	-1.03666200
C	0	-10.61326300	1.81214900	-1.83667300
C	0	-9.47536400	3.91409100	-1.43337600
C	0	-11.09501700	2.30890500	-3.03758400
H	0	-10.84975000	0.80192100	-1.52225500
C	0	-9.97029500	4.40739700	-2.62946200
H	0	-8.84265300	4.52449900	-0.80026100
C	0	-10.77680600	3.60397800	-3.43175000
H	0	-11.71850000	1.68365200	-3.66614400
H	0	-9.72990200	5.41828900	-2.93904400
H	0	-11.16013800	3.99202300	-4.36959900
C	0	3.88688800	3.31199900	-1.23960900
C	0	5.07496500	2.37867800	-1.23650700
H	0	3.07884400	2.89155900	-0.64631500
H	0	4.17076500	4.29002500	-0.84049700
H	0	3.51770500	3.43712600	-2.26035400
C	0	6.20262000	2.92476100	-2.09168800
H	0	4.79334900	1.38155600	-1.58140800
C	0	7.52129900	2.17935500	-2.03070800
H	0	5.84237900	2.89854100	-3.12447000

H	0	6.38942400	3.97675300	-1.84936900
H	0	7.35201200	1.12023000	-1.81693000
C	0	8.35748800	2.35969200	-3.27890000
H	0	8.50012300	3.42226400	-3.49425500
H	0	9.33311200	1.88287500	-3.17876400
H	0	7.84219100	1.90032200	-4.12574900
O	0	5.56710200	2.24576600	0.12335500
O	0	8.22691200	2.74521600	-0.88150100
C	0	5.01790200	1.35316300	0.92198900
O	0	4.09511500	0.62266400	0.57029300
C	0	9.19822700	2.06065400	-0.34054200
O	0	9.59995400	0.99835500	-0.81563100
C	0	5.59471000	1.32206600	2.26953200
C	0	5.14093500	0.35461900	3.16650700
C	0	6.61715900	2.19926800	2.63392300
C	0	5.71794400	0.26326000	4.42248800
H	0	4.36390000	-0.33594800	2.86211900
C	0	7.19205200	2.10058300	3.88913500
H	0	6.96347800	2.93944400	1.92525000
C	0	6.74388300	1.13109400	4.78164900
H	0	5.37779900	-0.49656600	5.11657300
H	0	7.99888600	2.77035400	4.16486200
H	0	7.20182300	1.04708300	5.76159000
C	0	9.76569700	2.65245200	0.87745400
C	0	10.61646900	1.87291500	1.66213900
C	0	9.43559100	3.95196500	1.26922100
C	0	11.12575600	2.39078300	2.84264600
H	0	10.86294500	0.86282700	1.35543500
C	0	9.95820500	4.46702300	2.44413500
H	0	8.77559200	4.54564700	0.64825100
C	0	10.79975300	3.68534700	3.23168600
H	0	11.77703900	1.78187900	3.45892800
H	0	9.71202200	5.47810300	2.74855300
H	0	11.20466700	4.09001900	4.15328000
Cl	0	1.84001200	0.60601000	-1.62716800
Cl	0	-1.77604500	0.65484500	1.56320600
Ti	0	0.04664100	0.89119900	-0.01943600
C	0	-1.04645400	2.20140500	-1.37752600
C	0	0.90774100	2.59531800	0.99556600
C	0	-0.24161500	3.21280100	-0.84919000
H	0	-2.10786000	2.16696500	-1.15312900
H	0	-0.73726500	1.72532000	-2.30411400
C	0	0.40640100	2.97673800	2.37208900
H	0	1.57567000	3.36461800	0.60900000
H	0	1.57611400	1.69672800	1.06537500
H	0	0.71030600	3.36081100	-1.35299700

C	0	-0.79896300	4.43816600	-0.19635900
H	0	1.23257800	3.35548900	2.98569100
H	0	-0.33863800	3.77505900	2.31725200
H	0	-0.05066500	2.13991200	2.89845500
H	0	-0.04002300	5.01554900	0.33371600
H	0	-1.20889300	5.07212600	-0.99044200
H	0	-1.61122500	4.20679300	0.49125300

$TiCl_2Et$-$MgCl_2$-2*SS*-PDDB-TS-*si*

0 2

Mg	-1	12.55747900	-2.73103200	-1.05739900
Mg	-1	9.41983100	-0.91415900	-0.78024800
Mg	-1	6.28096500	-2.73344900	-0.53453000
Mg	-1	3.14331600	-0.91657500	-0.25738000
Mg	-1	0.00445000	-2.73586500	-0.01166200
Mg	-1	-3.13319800	-0.91899100	0.26548900
Mg	-1	-6.27206500	-2.73828100	0.51120600
Mg	-1	-9.40971300	-0.92140800	0.78835700
Mg	-1	-12.54857900	-2.74069800	1.03407500
Cl	0	14.34822200	-3.98020700	-1.29903500
Cl	0	11.31810800	-1.18547300	-2.41841400
Cl	0	8.26184900	-2.96530700	-1.80628300
Cl	0	4.92172700	-1.30912400	-1.95323800
Cl	0	1.85549400	-2.98576800	-1.30779000
Cl	0	-1.19007800	-0.95460900	-1.32028300
Cl	0	-4.47500900	-2.85544500	-1.00224100
Cl	0	-7.55776400	-1.00579400	-0.75372300
Cl	0	-11.08561200	-2.09541300	-0.72941800
Cl	0	11.09821300	-2.10752100	0.71771900
Cl	0	7.56567300	-1.01886400	0.75704000
Cl	0	4.48815400	-2.86558200	0.98957800
Cl	0	1.16809900	-0.92412900	1.30960100
Cl	0	-1.82688800	-2.99610400	1.31684700
Cl	0	-4.91415700	-1.32482500	1.95031500
Cl	0	-8.25011300	-2.98337000	1.78576900
Cl	0	-11.31028600	-1.21527300	2.41868300
Cl	0	-14.34174700	-3.98998800	1.25519600
O	0	-9.25527700	1.05123500	0.91552300
C	0	-9.03473500	2.14513600	0.39589000
O	0	-8.29706700	3.06136400	0.96485900
C	0	-9.58001000	2.54855900	-0.91002800
C	0	-7.61624700	2.82124300	2.23833000
C	0	-10.22940200	1.60208100	-1.70269900
C	0	-9.43555000	3.86198100	-1.36649100
C	0	-8.59050900	2.51678800	3.35896200
C	0	-6.50765800	1.79411700	2.09621500

H	0	-7.17937000	3.80557800	2.41765000
C	0	-10.72398600	1.96348500	-2.94556500
H	0	-10.34377400	0.57945200	-1.36563300
C	0	-9.94021800	4.22014500	-2.60567900
H	0	-8.93140000	4.58992000	-0.74317200
H	0	-8.97999400	1.50091100	3.31056400
H	0	-8.07218300	2.64364000	4.31271900
H	0	-9.42871600	3.21707000	3.33774000
C	0	-5.35402100	2.11643800	1.16041500
H	0	-6.07040900	1.66548300	3.09242500
H	0	-6.90758700	0.81555300	1.82707000
C	0	-10.58286000	3.27081200	-3.39592600
H	0	-11.21823100	1.22049100	-3.56045000
H	0	-9.83601600	5.24064600	-2.95686700
O	0	-5.80567800	1.96994800	-0.21575800
H	0	-4.58365500	1.36917300	1.33574100
C	0	-4.75721000	3.49956200	1.29056400
H	0	-10.97651500	3.55324800	-4.36666900
C	0	-5.13859400	1.18855700	-1.03796600
H	0	-4.44968500	3.67703400	2.32447300
H	0	-5.46711100	4.27445700	0.99091000
H	0	-3.86963500	3.58142400	0.65896200
O	0	-4.16075600	0.52821100	-0.69453700
C	0	-5.63416000	1.19501800	-2.41802900
C	0	-5.13687800	0.23615500	-3.30157400
C	0	-6.59205400	2.11732900	-2.83968500
C	0	-5.60877800	0.19715500	-4.60320600
H	0	-4.41007400	-0.48647900	-2.95034700
C	0	-7.05311700	2.07741200	-4.14486100
H	0	-6.97138500	2.85359200	-2.14359000
C	0	-6.56483800	1.11576400	-5.02412300
H	0	-5.23752000	-0.55676600	-5.28779400
H	0	-7.80014800	2.79101500	-4.47272200
H	0	-6.93457500	1.07949200	-6.04350600
O	0	9.25708300	1.05867000	-0.88183100
C	0	9.02978400	2.14671300	-0.35290600
O	0	8.29094700	3.06516700	-0.91653800
C	0	9.56847300	2.54000400	0.95881100
C	0	7.60847600	2.83026200	-2.19026800
C	0	10.21137900	1.58675500	1.74861500
C	0	9.42494700	3.85073000	1.42299600
C	0	8.57854700	2.52428500	-3.31417500
C	0	6.49585800	1.80773500	-2.04617600
H	0	7.17560000	3.81675400	-2.36715400
C	0	10.70090500	1.93900200	2.99609000
H	0	10.32360500	0.56581900	1.40574100

C	0	9.92544800	4.19998700	2.66639000
H	0	8.92551300	4.58376300	0.80184600
H	0	8.96009400	1.50517400	-3.27311700
H	0	8.05918700	2.66114400	-4.26601600
H	0	9.42247700	3.21761700	-3.29071800
C	0	5.34512800	2.13800900	-1.10865100
H	0	6.05729500	1.67879200	-3.04173600
H	0	6.89241400	0.82812400	-1.77570500
C	0	10.56180500	3.24400700	3.45364200
H	0	11.18974600	1.19078100	3.60894300
H	0	9.82276400	5.21868000	3.02329400
O	0	5.79372800	1.98342700	0.26665600
H	0	4.56411500	1.40220900	-1.28622500
C	0	4.76671400	3.52936800	-1.23518300
H	0	10.95190800	3.51933000	4.42785900
C	0	5.14248500	1.17683900	1.07939600
H	0	4.46502000	3.71529600	-2.26942400
H	0	5.48622200	4.29345200	-0.93059800
H	0	3.87869800	3.62118400	-0.60592200
O	0	4.18115600	0.49862400	0.73169100
C	0	5.64747500	1.17715700	2.45750200
C	0	5.19350000	0.18448700	3.32658000
C	0	6.57508200	2.12536900	2.88904100
C	0	5.67668000	0.13909200	4.62393700
H	0	4.49317800	-0.55941800	2.96595400
C	0	7.04715300	2.07908100	4.19031500
H	0	6.92274800	2.88673100	2.20333400
C	0	6.60111600	1.08478900	5.05539800
H	0	5.33930800	-0.64091800	5.29678000
H	0	7.77024500	2.81341700	4.52596800
H	0	6.97954800	1.04389600	6.07142100
Cl	0	1.90262300	0.81145000	-1.50748300
Ti	0	-0.02605000	0.95749100	-0.00875300
Cl	0	-1.87396100	0.80534000	1.53973800
C	0	-1.14576100	2.16221000	-1.48687300
C	0	0.74901500	2.72665200	0.90110500
C	0	-0.62224300	3.25735500	-0.81108900
H	0	-2.15384100	1.82799400	-1.26343100
H	0	-0.73757600	1.87848600	-2.45269800
C	0	0.22066800	3.69631900	1.92944500
H	0	1.67119900	3.07041600	0.42867300
H	0	1.02590300	1.77840200	1.44212900
H	0	-1.24843400	3.70751900	-0.04600000
C	0	0.37820400	4.16315700	-1.46368500
H	0	0.93372800	3.82617700	2.75106200
H	0	0.05849500	4.68697300	1.49291600

H	0	-0.72195600	3.34879100	2.36018200
H	0	0.89081000	4.81348900	-0.75285600
H	0	1.11803800	3.58978000	-2.02629900
H	0	-0.16124200	4.80225700	-2.17121600

$TiCl_2Et$-$MgCl_2$-2*SS*-PDDB-TS-*re*

0 2

Mg	-1	-12.55615200	-2.83764800	0.80949500
Mg	-1	-9.42186600	-1.00221600	0.63595600
Mg	-1	-6.27491400	-2.80126900	0.34824100
Mg	-1	-3.14057000	-0.96593500	0.17472400
Mg	-1	0.00635100	-2.76504100	-0.11300900
Mg	-1	3.14067500	-0.92967400	-0.28655900
Mg	-1	6.28756900	-2.72882700	-0.57427400
Mg	-1	9.42181100	-0.89331300	-0.74775700
Mg	-1	12.56882700	-2.69224500	-1.03552100
Cl	0	-14.33753000	-4.11615800	0.94191900
Cl	0	-11.31565000	-1.40247500	2.27690900
Cl	0	-8.28302200	-3.12918800	1.53954600
Cl	0	-4.90771500	-1.51165900	1.87396400
Cl	0	-1.84685900	-3.06666800	1.16398900
Cl	0	1.17359800	-0.98297900	1.24166100
Cl	0	4.47778200	-2.87811200	0.93229400
Cl	0	7.55766400	-1.03512500	0.76478100
Cl	0	11.10523000	-2.07915300	0.74257600
Cl	0	-11.10411500	-2.06472600	-0.92452700
Cl	0	-7.52720700	-0.97688000	-0.84080500
Cl	0	-4.47173200	-2.81084600	-1.18247100
Cl	0	-1.18219700	-0.96162500	-1.41624600
Cl	0	1.85878600	-2.99260000	-1.41187000
Cl	0	4.93822900	-1.28025000	-1.97588700
Cl	0	8.28333600	-2.93564200	-1.82284900
Cl	0	11.32830800	-1.14433700	-2.38929500
Cl	0	14.36285000	-3.93738500	-1.27323200
O	0	9.28206900	1.07279900	-0.83524700
C	0	9.05169500	2.17525800	-0.34074100
O	0	8.33888600	3.08382200	-0.95151100
C	0	9.55426700	2.59113300	0.97735200
C	0	7.67120000	2.79958600	-2.22394200
C	0	10.20742900	1.65759000	1.78268600
C	0	9.35887500	3.89694900	1.43593400
C	0	8.64314100	2.42329800	-3.32430100
C	0	6.53456000	1.81074000	-2.03823700
H	0	7.26009200	3.78423500	-2.45376200
C	0	10.65615400	2.02550300	3.04089100
H	0	10.35988200	0.64154700	1.44000300

C	0	9.81565200	4.26086500	2.69201200
H	0	8.85165100	4.61374300	0.80228600
H	0	9.00057400	1.39827900	-3.24171500
H	0	8.13415200	2.53499800	-4.28507000
H	0	9.50323400	3.09682700	-3.32180700
C	0	5.42120500	2.19906200	-1.07558100
H	0	6.06962200	1.68065200	-3.02147200
H	0	6.90486400	0.82275600	-1.76150200
C	0	10.46292400	3.32520200	3.49466000
H	0	11.15469300	1.29388800	3.66588800
H	0	9.67015800	5.27491300	3.04704700
O	0	5.85623300	1.95151700	0.29189500
H	0	4.57314200	1.54539800	-1.27186800
C	0	4.98652600	3.64613300	-1.13348300
H	0	10.81961100	3.61246800	4.47821200
C	0	5.15155300	1.15068000	1.06500800
H	0	4.65919000	3.90087700	-2.14513700
H	0	5.79803700	4.31687100	-0.84160600
H	0	4.14973600	3.81236500	-0.45351100
O	0	4.16707800	0.53026900	0.67074900
C	0	5.61281400	1.09212800	2.45640100
C	0	5.06513900	0.11842600	3.29270300
C	0	6.58232900	1.97275100	2.93710200
C	0	5.49754900	0.02205500	4.60495200
H	0	4.32902000	-0.57156100	2.89850200
C	0	7.00336700	1.87611100	4.25288900
H	0	7.00227400	2.72176100	2.27894900
C	0	6.46462000	0.89928700	5.08455200
H	0	5.08612500	-0.74404900	5.25201000
H	0	7.75847700	2.55818100	4.62630800
H	0	6.80308300	0.81874000	6.11221600
O	0	-9.29931800	0.94535100	0.90399800
C	0	-9.04774000	2.10082200	0.56635600
O	0	-8.34163100	2.91211000	1.30728900
C	0	-9.51968800	2.69066400	-0.69528300
C	0	-7.70725600	2.45032900	2.54513200
C	0	-10.10754400	1.86358700	-1.65290900
C	0	-9.36686600	4.05666100	-0.94679400
C	0	-8.71045600	1.94724000	3.56405500
C	0	-6.57971000	1.47645000	2.25264500
H	0	-7.28928400	3.38876100	2.91324800
C	0	-10.53578700	2.39908000	-2.85688500
H	0	-10.21955400	0.80201100	-1.46922300
C	0	-9.80690600	4.58809100	-2.14800900
H	0	-8.90962800	4.68864000	-0.19553800
H	0	-9.07687300	0.94618400	3.34285600

H	0	-8.22440000	1.92747100	4.54282100
H	0	-9.56358400	2.62655100	3.62874400
C	0	-5.41904900	1.98752100	1.41061800
H	0	-6.15823500	1.18222300	3.21980100
H	0	-6.95466000	0.55336000	1.80900700
C	0	-10.38993300	3.75940000	-3.10288900
H	0	-10.98245800	1.75157600	-3.60227300
H	0	-9.69909900	5.64957200	-2.34117300
O	0	-5.81709500	2.01511900	0.00933000
H	0	-4.60194100	1.27508000	1.50550400
C	0	-4.94042400	3.37778700	1.76413300
H	0	-10.73307000	4.17768600	-4.04332100
C	0	-5.17456500	1.28804100	-0.88229200
H	0	-4.70890800	3.42911300	2.83146500
H	0	-5.69706200	4.12995700	1.52793400
H	0	-4.03119000	3.62125600	1.21405000
O	0	-4.25001600	0.53160200	-0.60850100
C	0	-5.64169300	1.48587200	-2.26109200
C	0	-5.22535600	0.57580900	-3.23359600
C	0	-6.48863300	2.54200000	-2.59676900
C	0	-5.66402300	0.72215100	-4.53921500
H	0	-4.58979600	-0.25446300	-2.94845300
C	0	-6.91442700	2.68833800	-3.90713000
H	0	-6.80839300	3.23763700	-1.83190300
C	0	-6.50549300	1.77764100	-4.87602300
H	0	-5.35659500	0.00707600	-5.29363100
H	0	-7.57249100	3.50848700	-4.16977200
H	0	-6.84776700	1.88848600	-5.89961400
Cl	0	-1.89643900	0.73859300	1.43648200
Ti	0	0.01408400	0.91189800	-0.09833700
Cl	0	1.89653300	0.78832600	-1.59353000
C	0	1.04578100	2.17160500	1.39670400
C	0	-0.73870000	2.63552500	-1.10177700
C	0	0.41788900	3.22946200	0.75110100
H	0	2.10773600	1.99868700	1.24611700
H	0	0.60317500	1.75749400	2.29679300
C	0	-1.98300700	3.46454400	-0.91082300
H	0	-1.05388600	1.66312900	-1.57216800
H	0	-0.03712100	3.07815700	-1.81107700
C	0	1.20698300	4.26147700	0.00393200
H	0	-0.53584300	3.55380500	1.15833300
H	0	-2.53042400	3.58179200	-1.85302600
H	0	-2.65197100	2.98961800	-0.19135500
H	0	-1.74428400	4.47118600	-0.55297800
H	0	0.58618400	4.89760500	-0.62870200
H	0	1.70518700	4.90358000	0.73857500

H	0	1.97451700	3.79091100	-0.61338900

$TiCl_2Et$-$MgCl_2$-2*RS*-PDDB-H_2-TS

0 2

Mg	-1	-12.51307500	-2.71914700	1.09963300
Mg	-1	-9.38089300	-0.88107400	0.86891300
Mg	-1	-6.24215900	-2.67972500	0.50030700
Mg	-1	-3.11152500	-0.84434300	0.26797500
Mg	-1	0.02951400	-2.63901200	-0.10228100
Mg	-1	3.15780800	-0.79439600	-0.33253300
Mg	-1	6.30049100	-2.58631000	-0.70115600
Mg	-1	9.42711800	-0.74444400	-0.93282900
Mg	-1	12.56826400	-2.53905700	-1.30320100
Cl	0	-14.31606800	-3.96273200	1.27063500
Cl	0	-11.21268400	-1.32046600	2.55115200
Cl	0	-8.21771600	-3.05056800	1.70423000
Cl	0	-4.91597200	-1.19295100	1.92612800
Cl	0	-1.79994500	-2.93927000	1.23301800
Cl	0	1.19740700	-0.90227900	1.25999300
Cl	0	4.50701500	-2.83809400	0.78608600
Cl	0	7.59362500	-0.89071800	0.61868800
Cl	0	11.14319500	-1.96833900	0.51210700
Cl	0	-11.10260100	-1.95820400	-0.66658600
Cl	0	-7.50519300	-0.80227000	-0.61800200
Cl	0	-4.46301700	-2.73780100	-1.03160100
Cl	0	-1.18522400	-0.84938100	-1.35181800
Cl	0	1.85994600	-2.82296100	-1.45297300
Cl	0	4.98672800	-0.95235600	-1.97484600
Cl	0	8.25740700	-2.80083700	-1.99234500
Cl	0	11.28773700	-0.98620900	-2.61184900
Cl	0	14.37262300	-3.75801600	-1.59673600
C	0	3.67917100	3.56521700	-0.37259000
C	0	4.86365000	2.66989400	-0.65677700
H	0	2.94647900	3.03842300	0.23863300
H	0	4.00279900	4.47081600	0.14800300
H	0	3.19447700	3.84534300	-1.31071100
C	0	5.91764000	3.35927600	-1.50069700
H	0	4.54505100	1.75128700	-1.15166500
C	0	7.21994600	2.59897400	-1.67972600
H	0	5.47103000	3.51568100	-2.48718200
H	0	6.14802200	4.35069800	-1.09580700
H	0	7.05129000	1.51979200	-1.61919600
C	0	7.94736900	2.96495100	-2.95468400
H	0	8.10020200	4.04615900	-3.01381500
H	0	8.91392700	2.46410100	-3.01663700
H	0	7.34780300	2.65101700	-3.81240900

O	0	5.47674600	2.29956200	0.60345300
O	0	8.03737800	2.97243400	-0.52542300
C	0	4.99509200	1.26175800	1.25872500
O	0	4.06912500	0.57980300	0.82809700
C	0	9.02989600	2.20176900	-0.17385600
O	0	9.37261500	1.22567600	-0.84361900
C	0	5.65628100	1.00726400	2.54092400
C	0	5.28987400	-0.12459600	3.26928400
C	0	6.67845400	1.84125800	2.99711500
C	0	5.95610300	-0.42382800	4.44625900
H	0	4.51219700	-0.77591800	2.88981400
C	0	7.34270100	1.53433300	4.17158500
H	0	6.95512600	2.71353800	2.42023500
C	0	6.98329300	0.40014200	4.89383200
H	0	5.68446000	-1.31062200	5.00714800
H	0	8.14914300	2.17204100	4.51604400
H	0	7.51123400	0.15364100	5.80902400
C	0	9.69155500	2.59473400	1.07788100
C	0	10.60657800	1.71802000	1.66127100
C	0	9.38713700	3.80898800	1.69904800
C	0	11.20394200	2.04902500	2.86709400
H	0	10.84183000	0.77320600	1.18602900
C	0	9.99742800	4.13966100	2.89755700
H	0	8.67665800	4.48214500	1.23497400
C	0	10.90250300	3.25824000	3.48336300
H	0	11.90399300	1.35978900	3.32486700
H	0	9.76891400	5.08438600	3.37800400
H	0	11.37565500	3.51750000	4.42457700
C	0	-3.80941100	3.51380500	0.97406000
C	0	-4.96792600	2.54962000	1.08305200
H	0	-3.02446200	3.08927300	0.35203200
H	0	-4.14386000	4.46412400	0.54843600
H	0	-3.38302200	3.69609200	1.96307900
C	0	-6.06979600	3.10218400	1.96707400
H	0	-4.63759500	1.58162800	1.46425200
C	0	-7.37284600	2.32605800	1.98536300
H	0	-5.66677700	3.12041700	2.98401500
H	0	-6.29220700	4.14032300	1.69694200
H	0	-7.19346400	1.26388000	1.79487200
C	0	-8.15796900	2.52889900	3.26286700
H	0	-8.31232300	3.59472100	3.45266300
H	0	-9.12714800	2.03130200	3.21906800
H	0	-7.59731900	2.10523100	4.09946800
O	0	-5.51999600	2.33232200	-0.24184200
O	0	-8.13722100	2.84443900	0.85095600
C	0	-4.98513900	1.40830700	-1.01455300

O	0	-4.04082100	0.70749000	-0.65762300
C	0	-9.12108500	2.13222600	0.37326000
O	0	-9.49213800	1.08378200	0.90247200
C	0	-5.60317300	1.30647600	-2.33948500
C	0	-5.16323200	0.30387700	-3.20413500
C	0	-6.65134100	2.15026300	-2.71062100
C	0	-5.77951300	0.14381700	-4.43427100
H	0	-4.36678800	-0.36065200	-2.89208000
C	0	-7.26552900	1.98246500	-3.93934400
H	0	-6.98792100	2.91768100	-2.02664100
C	0	-6.83109500	0.97782400	-4.79929400
H	0	-5.45015500	-0.64348200	-5.10243800
H	0	-8.09235800	2.62572800	-4.21907000
H	0	-7.32008000	0.83965200	-5.75784200
C	0	-9.73921000	2.67832700	-0.84187400
C	0	-10.62153900	1.87448400	-1.56464500
C	0	-9.42322600	3.96220000	-1.29330100
C	0	-11.17530000	2.35124400	-2.74253600
H	0	-10.86018800	0.87654800	-1.21538900
C	0	-9.98985800	4.43649900	-2.46482000
H	0	-8.73841400	4.57596500	-0.72075200
C	0	-10.86242000	3.62969200	-3.19083900
H	0	-11.85086400	1.72201400	-3.31034600
H	0	-9.75358600	5.43518000	-2.81457100
H	0	-11.30198400	4.00189900	-4.11021800
Cl	0	1.85437800	0.91601500	-1.56491500
Cl	0	-1.79786800	0.83138300	1.55617100
Ti	0	0.04547100	0.97064500	0.03907900
C	0	-0.16567000	3.17816500	-0.19845700
H	0	1.09734500	1.74767800	1.25054100
H	0	0.66023300	2.46744100	0.73984700
C	0	-0.95780600	2.69281100	-1.39319800
H	0	-0.79087700	3.68042100	0.54082400
H	0	0.65015500	3.83123600	-0.50675200
H	0	-1.37515700	1.65653000	-1.31618700
H	0	-1.87541700	3.26732000	-1.53956400
H	0	-0.37574100	2.69764000	-2.31412200

$TiCl_2Et$-$MgCl_2$-2*SS*-PDDB-H_2-TS

0 2

Mg	-1	-12.53508600	-2.68847400	0.98462000
Mg	-1	-9.40061000	-0.85801000	0.76704800
Mg	-1	-6.25906100	-2.66262800	0.45682800
Mg	-1	-3.12449600	-0.83222700	0.23945800
Mg	-1	0.01696800	-2.63688200	-0.07117200
Mg	-1	3.15162700	-0.80651700	-0.28839300

Mg	-1	6.29307100	-2.61098200	-0.59877700
Mg	-1	9.42751200	-0.78053400	-0.81630200
Mg	-1	12.56911500	-2.58519400	-1.12658300
Cl	0	-14.32580100	-3.94454000	1.18395800
Cl	0	-11.29505600	-1.18892500	2.39637000
Cl	0	-8.23628500	-2.94483100	1.71818900
Cl	0	-4.90025600	-1.27703700	1.92015700
Cl	0	-1.82293000	-2.92006100	1.23936600
Cl	0	1.18518900	-0.88649900	1.30370500
Cl	0	4.48878500	-2.81969700	0.89713000
Cl	0	7.57859700	-0.94139100	0.73249200
Cl	0	11.09570400	-2.02778200	0.65510900
Cl	0	-11.07218300	-2.00724800	-0.76777800
Cl	0	-7.54095900	-0.89728300	-0.76799300
Cl	0	-4.46195200	-2.74094600	-1.06670700
Cl	0	-1.15567500	-0.80712000	-1.32585600
Cl	0	1.85176300	-2.81894700	-1.40732000
Cl	0	4.92806700	-1.14086200	-1.98154600
Cl	0	8.26250900	-2.79495800	-1.90001500
Cl	0	11.33308900	-0.99920900	-2.44677500
Cl	0	14.36603600	-3.81775800	-1.40468900
O	0	9.25890100	1.19332500	-0.84154900
C	0	9.02751500	2.26050800	-0.27266800
O	0	8.29151400	3.19894700	-0.80572100
C	0	9.55728300	2.60233600	1.05663800
C	0	7.60980300	3.00744000	-2.08707700
C	0	10.17712900	1.61279700	1.81960200
C	0	9.42835300	3.89840100	1.56429000
C	0	8.57885100	2.73757100	-3.22112800
C	0	6.49526400	1.98255600	-1.97579800
H	0	7.17838700	3.99975400	-2.23140300
C	0	10.65953700	1.91419800	3.08281200
H	0	10.27516900	0.60241800	1.44341800
C	0	9.92295000	4.19719100	2.82316500
H	0	8.94571100	4.66001400	0.96449100
H	0	8.95647200	1.71620100	-3.21659500
H	0	8.05982800	2.91028700	-4.16732300
H	0	9.42550800	3.42629000	-3.17331900
C	0	5.34558900	2.28683800	-1.02771600
H	0	6.05666800	1.88594200	-2.97500100
H	0	6.88963500	0.99411600	-1.73543900
C	0	10.53701200	3.20513000	3.58296100
H	0	11.12973800	1.13757200	3.67453100
H	0	9.83265400	5.20465800	3.21370500
O	0	5.79323400	2.08412400	0.34122900
H	0	4.56169600	1.56060600	-1.22994300

C	0	4.77327900	3.68381500	-1.10691900
H	0	10.92197600	3.44110300	4.56946900
C	0	5.15594600	1.23478600	1.12063000
H	0	4.47975800	3.90825600	-2.13578100
H	0	5.49453700	4.43266800	-0.77037600
H	0	3.88165400	3.75582500	-0.48042600
O	0	4.20665000	0.55446000	0.74562600
C	0	5.66421900	1.18694600	2.49648800
C	0	5.23986800	0.14387900	3.32019200
C	0	6.56645200	2.13993200	2.96928200
C	0	5.72749600	0.05278900	4.61330800
H	0	4.55984700	-0.60238300	2.92683500
C	0	7.04204200	2.04801700	4.26693900
H	0	6.89078400	2.94146400	2.31855400
C	0	6.62584100	1.00353200	5.08656600
H	0	5.41315300	-0.76589900	5.25041300
H	0	7.74387900	2.78707800	4.63566600
H	0	7.00681900	0.92787700	6.09964200
O	0	-9.25156500	1.11001100	0.93156600
C	0	-9.02398600	2.21908300	0.44883100
O	0	-8.29238900	3.11594500	1.05486900
C	0	-9.55294300	2.66134300	-0.85110200
C	0	-7.61586800	2.82790900	2.32093900
C	0	-10.16452100	1.73123700	-1.69176400
C	0	-9.43054000	3.99419600	-1.25423400
C	0	-8.58951900	2.47048500	3.42643600
C	0	-6.49879400	1.81643100	2.13799000
H	0	-7.18679900	3.80692600	2.54261400
C	0	-10.64509200	2.12895600	-2.92894700
H	0	-10.25597800	0.69357000	-1.39656500
C	0	-9.92250700	4.38848700	-2.48764200
H	0	-8.95543800	4.70920200	-0.59408100
H	0	-8.96880600	1.45338000	3.33940600
H	0	-8.07370000	2.56632100	4.38521100
H	0	-9.43489600	3.16243200	3.43093100
C	0	-5.34712300	2.19045200	1.21783400
H	0	-6.06184500	1.65010600	3.12867800
H	0	-6.88853700	0.84602900	1.82706900
C	0	-10.52855400	3.45602000	-3.32537500
H	0	-11.10930200	1.39906400	-3.58175300
H	0	-9.83741600	5.42419900	-2.79700200
O	0	-5.78931700	2.08180200	-0.16492800
H	0	-4.56134800	1.45474200	1.37232200
C	0	-4.78348200	3.58185400	1.39738600
H	0	-10.91244700	3.76695900	-4.29131400
C	0	-5.13135900	1.31251400	-1.00643200

H	0	-4.49347200	3.73500600	2.44012500
H	0	-5.50810800	4.34824500	1.11165400
H	0	-3.88941400	3.70629600	0.78245600
O	0	-4.16232400	0.63058900	-0.68435900
C	0	-5.63262200	1.36372300	-2.38476600
C	0	-5.17458200	0.40484500	-3.28923000
C	0	-6.55884400	2.32765200	-2.78365100
C	0	-5.65253700	0.40837100	-4.58916800
H	0	-4.47616300	-0.35293900	-2.95475100
C	0	-7.02453800	2.33109600	-4.08809700
H	0	-6.90963300	3.06248900	-2.07093900
C	0	-6.57482100	1.37018300	-4.98834400
H	0	-5.31289800	-0.34601100	-5.28951800
H	0	-7.74524400	3.07837500	-4.39911100
H	0	-6.94868800	1.36822000	-6.00685900
Cl	0	-1.89505200	0.91110800	1.51579500
Cl	0	1.90302800	1.02413100	-1.43518600
Ti	0	0.04545300	1.01557100	0.10369700
H	0	1.01995200	1.76474500	1.39862100
C	0	-1.08374800	2.75924300	-1.19015700
H	0	0.60132600	2.49208600	0.89726000
C	0	-0.15968000	3.22542400	-0.08830400
H	0	-0.99612500	1.68370000	-1.50285400
H	0	-2.13685300	2.86141200	-0.93041900
H	0	-0.87593400	3.25652300	-2.13906200
H	0	-0.68960300	3.81137300	0.66265100
H	0	0.69228100	3.78350000	-0.47731700

RS-HDDB

0 1

C	-0.04273400	-1.39851100	0.65509600
C	1.30449000	-1.59134500	0.06361900
C	1.48578800	-2.00887400	-1.25405000
C	2.40787600	-1.30673000	0.86486600
C	2.76899700	-2.13485800	-1.76618000
H	0.61868900	-2.22258800	-1.86665000
C	3.68794300	-1.43573600	0.35039300
H	2.23499900	-0.96853400	1.87965800
C	3.86955300	-1.84866800	-0.96528800
H	2.91280900	-2.45744300	-2.79189900
H	4.54627700	-1.20536600	0.97192600
H	4.87213000	-1.94660900	-1.36892600
O	-0.22973100	-1.00431900	1.77929700
C	-2.38506200	-1.52122300	0.21622800
H	-2.36109900	-1.18117500	1.25565400
O	-1.01990900	-1.70209000	-0.21048100

C	-3.07273600	-2.87329700	0.12694100
H	-4.11291900	-2.73632200	0.44367000
H	-3.10158800	-3.18743400	-0.92360400
C	-2.40377000	-3.94054400	0.97872300
H	-2.95210800	-4.88433500	0.92722000
H	-1.38257400	-4.12852800	0.63843200
H	-2.35477000	-3.63392100	2.02772800
C	-3.03103200	-0.47254800	-0.67612500
H	-4.11786600	-0.60064700	-0.62312900
H	-2.73713500	-0.66286700	-1.71468000
C	-2.73050500	0.97050500	-0.31112500
H	-2.99576400	1.16219400	0.73313400
C	-3.45296300	1.95388900	-1.21843300
H	-4.52803800	1.76286900	-1.11942000
H	-3.18852300	1.72687700	-2.25845900
C	-3.15369800	3.41254200	-0.90893900
H	-3.74725100	4.07314300	-1.54598100
H	-3.37358300	3.65076300	0.13466500
H	-2.10000000	3.64360500	-1.08468500
O	-1.31146700	1.17129000	-0.43529100
C	-0.67831700	1.81818700	0.56131100
C	0.78065400	1.91008100	0.30618900
C	1.34519300	1.53897200	-0.91343500
C	1.59251900	2.37397100	1.33876200
C	2.71642500	1.63597800	-1.09734100
H	0.70480900	1.16794600	-1.70345900
C	2.96372400	2.46266500	1.15399000
H	1.12529800	2.65064000	2.27675900
C	3.52544300	2.09557800	-0.06480700
H	3.15659000	1.33711400	-2.04202700
H	3.59692700	2.81780900	1.95994800
H	4.59891800	2.16297100	-0.20860600
O	-1.22651300	2.26257300	1.53652000

SS-HDDB

0 1

C	-0.66857394	1.60163503	0.58379286
C	0.76234906	1.89944401	0.32852685
C	1.35633007	1.67651395	-0.91292514
C	1.52560806	2.37737507	1.39195683
C	2.71007507	1.92337293	-1.08360214
H	0.75509807	1.29408391	-1.72802312
C	2.87685006	2.63172305	1.21531682
H	1.03806406	2.53552912	2.34721982
C	3.46985007	2.40105798	-0.02170717

H	3.17655307	1.73113688	-2.04325713
H	3.47079306	3.00629109	2.04217280
H	4.52963707	2.59016797	-0.15781817
O	-1.18435994	1.67484309	1.67324585
C	-2.69502594	0.84956099	-0.42555611
H	-3.01504994	1.04515004	0.60070488
O	-1.31048694	1.23910398	-0.53303213
C	-2.79738594	-0.62755103	-0.76224503
H	-3.85815295	-0.88869803	-0.84016202
C	-2.18350395	-1.60583598	0.23317502
H	-2.35660796	-2.61660800	-0.15233993
C	-2.71489096	-1.48547690	1.64905001
H	-2.41972096	-0.51263988	2.05450596
H	-3.80994496	-1.49503190	1.59742301
H	-2.35583794	-0.80325008	-1.74734402
C	-2.22236397	-2.60190986	2.55705607
H	-2.61508797	-2.48718480	3.57029006
H	-1.13142897	-2.59842386	2.61725208
H	-2.53444597	-3.58309587	2.18478112
O	-0.75414295	-1.43428298	0.33504202
C	-0.00575995	-1.90917904	-0.66721395
C	1.44369005	-1.69823303	-0.42870096
C	1.93037705	-1.18132997	0.77150402
C	2.32501605	-2.02223909	-1.45800194
C	3.29357605	-0.98866097	0.93575401
H	1.23598604	-0.92010792	1.56007200
C	3.68718405	-1.82363808	-1.29172394
H	1.91803205	-2.42570013	-2.37826792
C	4.17133205	-1.30727802	-0.09444297
H	3.67092204	-0.57682592	1.86487199
H	4.37332305	-2.07261413	-2.09415693
H	5.23710705	-1.14983202	0.03552403
O	-0.46065195	-2.44210108	-1.65029393
C	-3.47420893	1.73920894	-1.38159116
H	-3.15179593	1.52716989	-2.40753915
H	-3.18601993	2.77396595	-1.17043921
C	-4.98356693	1.59226696	-1.25352416
H	-5.49688193	2.31275992	-1.89470720
H	-5.31149594	1.77203401	-0.22471817
H	-5.32765294	0.59625794	-1.54512611

$TiCl_4$-$MgCl_2$-2*RS*-HDDB

0 1

Mg	-1	-12.57677600	-2.87323600	0.70443200
Mg	-1	-9.43257700	-1.05508500	0.52830300
Mg	-1	-6.28837600	-2.87323400	0.35222100

Mg	-1	-3.14417700	-1.05508200	0.17609900
Mg	-1	0.00002600	-2.87323200	0.00001800
Mg	-1	3.14422500	-1.05508100	-0.17609900
Mg	-1	6.28842800	-2.87323000	-0.35216700
Mg	-1	9.43262700	-1.05507900	-0.52828400
Mg	-1	12.57683000	-2.87322800	-0.70435100
Cl	0	-14.36579500	-4.15382600	0.81487400
Cl	0	-11.34502500	-1.43942000	2.17787500
Cl	0	-8.30613600	-3.21179500	1.51886000
Cl	0	-4.97933500	-1.41294400	1.82566800
Cl	0	-1.86684800	-3.15434500	1.26933500
Cl	0	1.14084400	-1.03092400	1.30835500
Cl	0	4.41838700	-2.93765000	1.12285100
Cl	0	7.53281300	-1.14145800	0.94701700
Cl	0	11.13839900	-2.08981100	1.04089200
Cl	0	-11.13833300	-2.08994300	-1.04084200
Cl	0	-7.53276700	-1.14149700	-0.94700500
Cl	0	-4.41834700	-2.93770400	-1.12279500
Cl	0	-1.14081600	-1.03099300	-1.30839700
Cl	0	1.86690800	-3.15437200	-1.26927300
Cl	0	4.97940900	-1.41296300	-1.82564100
Cl	0	8.30619500	-3.21185300	-1.51876600
Cl	0	11.34507100	-1.43949600	-2.17786600
Cl	0	14.36580000	-4.15389700	-0.81465900
C	0	3.73740900	3.28775000	-1.05914000
C	0	4.96316500	2.39450200	-1.18855400
H	0	3.02006200	2.78255800	-0.40935300
H	0	4.04127000	4.20843700	-0.54527200
C	0	6.03907100	3.03011200	-2.05024100
H	0	4.69333300	1.41065500	-1.58571900
C	0	7.38429300	2.32577900	-2.13793700
H	0	5.62622600	3.08411800	-3.06207600
H	0	6.21444700	4.06432700	-1.72919100
H	0	7.28148900	1.24020800	-2.04591100
C	0	8.12038300	2.70222500	-3.41711100
H	0	8.13055400	3.79730000	-3.49370700
H	0	7.49704000	2.33971200	-4.24327000
O	0	5.52003300	2.19390200	0.13811300
O	0	8.14319800	2.80103300	-0.98403800
C	0	4.97801600	1.29480800	0.93834300
O	0	4.05266700	0.55876600	0.58564900
C	0	8.98140400	2.00185900	-0.37591500
O	0	9.24042100	0.87263200	-0.80372000
C	0	5.55879500	1.26482700	2.28421800
C	0	5.15229700	0.26038000	3.16733000
C	0	6.53044000	2.19429500	2.66763900

C	0	5.72692400	0.18358000	4.42738200
H	0	4.41722200	-0.46965000	2.84778400
C	0	7.10338800	2.10938600	3.92751400
H	0	6.83511100	2.96793700	1.97369300
C	0	6.70327200	1.10302800	4.80523400
H	0	5.42393900	-0.60319500	5.11020800
H	0	7.86840800	2.82162300	4.21921100
H	0	7.15805100	1.03240900	5.78893000
C	0	9.58204000	2.56663900	0.84252800
C	0	10.38234500	1.75322000	1.64991900
C	0	9.33282500	3.89459100	1.20978300
C	0	10.91977000	2.26233100	2.82419400
H	0	10.58275900	0.72375100	1.37276800
C	0	9.87924900	4.39962600	2.38090200
H	0	8.71284400	4.51651600	0.57384200
C	0	10.66998500	3.58297600	3.18929800
H	0	11.53313700	1.62541800	3.45276100
H	0	9.69125500	5.43012900	2.66486300
H	0	11.09526600	3.97991400	4.10631300
C	0	-3.73742900	3.28747700	1.05922500
C	0	-4.96327400	2.39434900	1.18857700
H	0	-3.02009100	2.78218700	0.40950700
H	0	-4.04116500	4.20817600	0.54530300
C	0	-6.03914700	3.03006800	2.05022000
H	0	-4.69354100	1.41048700	1.58577100
C	0	-7.38440900	2.32581700	2.13793300
H	0	-5.62631500	3.08409600	3.06205900
H	0	-6.21445800	4.06427800	1.72911800
H	0	-7.28166400	1.24023500	2.04594300
C	0	-8.12048200	2.70235700	3.41708800
H	0	-8.13061400	3.79743700	3.49362000
H	0	-7.49715200	2.33987000	4.24326800
O	0	-5.52009500	2.19379000	-0.13811900
O	0	-8.14327200	2.80106500	0.98400800
C	0	-4.97804200	1.29472600	-0.93836300
O	0	-4.05266600	0.55872200	-0.58566300
C	0	-8.98142200	2.00186000	0.37585700
O	0	-9.24040800	0.87263700	0.80368600
C	0	-5.55884300	1.26469300	-2.28422700
C	0	-5.15230900	0.26024900	-3.16732500
C	0	-6.53053700	2.19410800	-2.66765200
C	0	-5.72694600	0.18339700	-4.42736900
H	0	-4.41719400	-0.46974100	-2.84777700
C	0	-7.10349600	2.10914600	-3.92751800
H	0	-6.83523400	2.96775000	-1.97371800
C	0	-6.70334300	1.10279100	-4.80522500

H	0	-5.42393200	-0.60337700	-5.11018400
H	0	-7.86855300	2.82134000	-4.21922000
H	0	-7.15813400	1.03213100	-5.78891200
C	0	-9.58209000	2.56662800	-0.84258100
C	0	-10.38237100	1.75318500	-1.64997100
C	0	-9.33294900	3.89459800	-1.20981800
C	0	-10.91983800	2.26228700	-2.82423100
H	0	-10.58273700	0.72370300	-1.37283500
C	0	-9.87941800	4.39962600	-2.38091900
H	0	-8.71299400	4.51654600	-0.57387400
C	0	-10.67012600	3.58295100	-3.18931800
H	0	-11.53318500	1.62535400	-3.45279800
H	0	-9.69148000	5.43014400	-2.66486500
H	0	-11.09544200	3.97988400	-4.10631800
Ti	0	0.00002800	0.88675100	-0.00008800
Cl	0	1.69876500	0.50296300	-1.54112200
Cl	0	1.04829800	2.30182400	1.29458300
Cl	0	-1.04820400	2.30173600	-1.29488700
Cl	0	-1.69872700	0.50309100	1.54098100
C	0	9.53182200	2.15431200	-3.56798100
H	0	10.20638200	2.54543100	-2.80117900
H	0	9.55592000	1.06324200	-3.50859400
H	0	9.94162700	2.44672000	-4.53827800
C	0	3.07251400	3.61649700	-2.39004800
H	0	2.10892100	4.10333000	-2.21763000
H	0	3.67787500	4.29283900	-3.00105300
H	0	2.87835500	2.71035300	-2.97164300
C	0	-3.07260300	3.61620500	2.39017200
H	0	-2.10895800	4.10295500	2.21780900
H	0	-3.67795300	4.29261500	3.00111300
H	0	-2.87856100	2.71006200	2.97180600
C	0	-9.53194000	2.15450400	3.56799700
H	0	-10.20649700	2.54561200	2.80118700
H	0	-9.55607900	1.06343300	3.50865700
H	0	-9.94172000	2.44696600	4.53828800

$TiCl_4$-$MgCl_2$-2*SS*-HDDB

0 1

Mg	-1	12.57301000	-2.82054400	-0.76645600
Mg	-1	9.42982200	-1.00210200	-0.57588300
Mg	-1	6.28650800	-2.81995800	-0.38182600
Mg	-1	3.14326300	-1.00161300	-0.19128000
Mg	-1	-0.00001800	-2.81952300	0.00279300
Mg	-1	-3.14324300	-1.00114600	0.19337300
Mg	-1	-6.28649900	-2.81910200	0.38742800
Mg	-1	-9.42964100	-1.00057600	0.57794700

Mg	-1	-12.57302000	-2.81831300	0.77205500
Cl	0	14.37375800	-4.08020400	-0.91283200
Cl	0	11.35735700	-1.33030800	-2.20214500
Cl	0	8.29662600	-3.11413200	-1.58491400
Cl	0	4.92464000	-1.42860100	-1.86779000
Cl	0	1.86227200	-3.09416800	-1.26904400
Cl	0	-1.12235100	-0.95022300	-1.29385500
Cl	0	-4.43948400	-2.92224600	-1.10016700
Cl	0	-7.50929900	-1.06143600	-0.88679900
Cl	0	-11.08623700	-2.14313900	-0.97466200
Cl	0	11.08724100	-2.13940600	0.97915000
Cl	0	7.50902500	-1.05952500	0.88861200
Cl	0	4.43954500	-2.91947700	1.10625900
Cl	0	1.12232400	-0.94765000	1.29584300
Cl	0	-1.86237500	-3.09139500	1.27538000
Cl	0	-4.92503200	-1.42429900	1.87019200
Cl	0	-8.29658700	-3.11026500	1.59167000
Cl	0	-11.35803300	-1.32360300	2.20382800
Cl	0	-14.37411900	-4.07723700	0.92060600
O	0	-9.27107500	0.96400500	0.77074600
C	0	-9.01056500	2.05299100	0.24458900
O	0	-8.26941200	2.96090100	0.82718900
C	0	-9.51334800	2.46134700	-1.07797400
C	0	-7.62406700	2.72223000	2.11732500
C	0	-10.14509500	1.52009300	-1.89531600
C	0	-9.34656900	3.77806600	-1.52585700
C	0	-8.63405600	2.42021600	3.21957000
C	0	-6.50990100	1.69699200	1.99341900
H	0	-7.19581600	3.71021600	2.31307500
C	0	-10.59822300	1.88945900	-3.15417400
H	0	-10.27828700	0.49650500	-1.56440600
C	0	-9.81206900	4.14442000	-2.78059500
H	0	-8.85657400	4.50186800	-0.88452200
H	0	-9.52521900	3.03149300	3.03726000
H	0	-8.95055900	1.37541900	3.17187100
C	0	-5.31246500	2.05213000	1.12379100
H	0	-6.12278900	1.51833100	3.00175700
H	0	-6.90167500	0.73334200	1.66048400
C	0	-10.43579900	3.19993100	-3.59567500
H	0	-11.07809800	1.15098800	-3.78754100
H	0	-9.69124300	5.16685100	-3.12419800
O	0	-5.70977000	1.95836600	-0.27111900
H	0	-4.55198700	1.29230200	1.30381700
C	0	-4.69975200	3.42777900	1.32346700
H	0	-10.79798700	3.48842900	-4.57801200
C	0	-5.03255400	1.17860600	-1.08833100

H	0	-5.42585100	4.20090800	1.04446500
H	0	-3.86454900	3.51760000	0.61949300
O	0	-4.07670600	0.48786700	-0.72423000
C	0	-5.48935100	1.21710200	-2.48122300
C	0	-4.98015700	0.26974800	-3.37431700
C	0	-6.43030300	2.15935500	-2.90628200
C	0	-5.42059600	0.26412400	-4.68984900
H	0	-4.26787200	-0.46787300	-3.02168000
C	0	-6.86145300	2.15067200	-4.22448700
H	0	-6.81872300	2.88557200	-2.20263100
C	0	-6.35923800	1.20241800	-5.11410100
H	0	-5.03779200	-0.47763700	-5.38306000
H	0	-7.59539500	2.87844800	-4.55420900
H	0	-6.70357600	1.19275600	-6.14410100
O	0	9.27237300	0.96221700	-0.77209100
C	0	9.01125800	2.05228500	-0.24845000
O	0	8.26960800	2.95845700	-0.83312200
C	0	9.51384900	2.46402300	1.07313700
C	0	7.62462900	2.71668600	-2.12284000
C	0	10.14516500	1.52478800	1.89312500
C	0	9.34724500	3.78197700	1.51745600
C	0	8.63496200	2.41263900	-3.22419700
C	0	6.51085000	1.69126600	-1.99682000
H	0	7.19597000	3.70405200	-2.32090900
C	0	10.59800300	1.89740500	3.15114100
H	0	10.27817500	0.50031100	1.56491300
C	0	9.81249200	4.15155700	2.77133700
H	0	8.85763900	4.50420800	0.87405600
H	0	8.95245400	1.36827100	-3.17370500
H	0	9.52559100	3.02518300	-3.04344800
C	0	5.31296000	2.04790900	-1.12847500
H	0	6.12410300	1.51019600	-3.00485500
H	0	6.90287300	0.72850800	-1.66157600
C	0	10.43575200	3.20907900	3.58911100
H	0	11.07752700	1.16051400	3.78661100
H	0	9.69184800	5.17493500	3.11217500
O	0	5.70991000	1.95828400	0.26678800
H	0	4.55320000	1.28693600	-1.30662000
C	0	4.69926300	3.42255200	-1.33205600
H	0	10.79772000	3.50010600	4.57078400
C	0	5.03256600	1.18088200	1.08612000
H	0	5.42464800	4.19695700	-1.05473400
H	0	3.86367600	3.51355600	-0.62869300
O	0	4.07727200	0.48843600	0.72386900
C	0	5.48863400	1.22396800	2.47913500
C	0	4.97818900	0.28017600	3.37528600

C	0	6.43008000	2.16698100	2.90141200
C	0	5.41786500	0.27885500	4.69108700
H	0	4.26534100	-0.45796900	3.02489800
C	0	6.86055500	2.16254700	4.21986000
H	0	6.81940900	2.89046500	2.19545900
C	0	6.35708900	1.21783400	5.11252900
H	0	5.03400900	-0.46008200	5.38673100
H	0	7.59493900	2.89086400	4.54739200
H	0	6.70086500	1.21150400	6.14274300
Ti	0	0.00014300	0.99282800	-0.00071700
Cl	0	1.73466000	0.63717100	-1.53059100
Cl	0	1.09163800	2.39673400	1.26370800
Cl	0	-1.09113900	2.39430800	-1.26800500
Cl	0	-1.73450500	0.64036300	1.52960100
C	0	8.07325900	2.72785700	-4.60560100
H	0	7.76814300	3.77702300	-4.68541300
H	0	8.82878500	2.54192300	-5.37275300
H	0	7.20687600	2.10515800	-4.84918300
C	0	4.19158300	3.62818200	-2.75339100
H	0	3.67672800	4.58810000	-2.84185700
H	0	5.00305700	3.62101700	-3.48848400
H	0	3.48098000	2.84308700	-3.03100100
C	0	-4.19163400	3.63743300	2.74404900
H	0	-3.67787700	4.59817000	2.82999100
H	0	-5.00275600	3.63102900	3.47954400
H	0	-3.47999000	2.85382300	3.02319400
C	0	-8.07243400	2.73975500	4.60004900
H	0	-7.76870300	3.78953800	4.67695000
H	0	-8.82750900	2.55487500	5.36789300
H	0	-7.20515000	2.11887000	4.84507600

$TiCl_2Et$-$MgCl_2$-2*RS*-HDDB

0 2

Mg	-1	-12.59631300	-2.69503700	0.92795500
Mg	-1	-9.44387800	-0.89900100	0.68336400
Mg	-1	-6.31105500	-2.73819300	0.52690700
Mg	-1	-3.15869500	-0.94214300	0.28231400
Mg	-1	-0.02557100	-2.78132600	0.12597100
Mg	-1	3.12663100	-0.98529300	-0.11872800
Mg	-1	6.25979300	-2.82452100	-0.27513100
Mg	-1	9.41194300	-1.02846500	-0.51970400
Mg	-1	12.54502000	-2.86766900	-0.67613400
Cl	0	-14.40886800	-3.91947000	1.13489900
Cl	0	-11.33407400	-1.20483800	2.32874100
Cl	0	-8.31068500	-2.98962900	1.73447500
Cl	0	-5.01612200	-1.18746900	1.90777200

Cl	0	-1.89317000	-2.99985500	1.39068000
Cl	0	1.18320200	-0.95845700	1.46704200
Cl	0	4.43319000	-2.82564300	1.22593500
Cl	0	7.47479600	-0.96721300	0.88851500
Cl	0	11.07520000	-2.12386000	1.04438600
Cl	0	-11.12529300	-2.05690100	-0.83230800
Cl	0	-7.53353800	-0.97722800	-0.76593600
Cl	0	-4.47366400	-2.90797400	-0.93543300
Cl	0	-1.19844400	-0.96867600	-1.28215200
Cl	0	1.83936100	-3.11861800	-1.10876500
Cl	0	4.95288000	-1.41538400	-1.77419800
Cl	0	8.26368200	-3.17412800	-1.44438900
Cl	0	11.29011500	-1.45272700	-2.16106800
Cl	0	14.35018000	-4.11210300	-0.81907800
C	0	3.85125600	3.28775900	-1.32890500
C	0	4.94945400	2.24026800	-1.28703100
H	0	3.05942700	2.96361400	-0.64945800
H	0	4.26061100	4.22639800	-0.93661900
C	0	6.10113800	2.54545200	-2.22818400
H	0	4.54986000	1.25096300	-1.51206700
C	0	7.38658000	1.77864200	-1.97466200
H	0	5.75429800	2.31211900	-3.23781500
H	0	6.33979400	3.61476800	-2.21326400
H	0	7.17578100	0.82596500	-1.47785000
C	0	8.20682300	1.55819200	-3.23672900
H	0	8.21144700	2.48398000	-3.82360400
H	0	9.24420700	1.34891500	-2.96537100
O	0	5.48307600	2.20326800	0.06385400
O	0	8.12450200	2.60275800	-1.01422500
C	0	4.97622400	1.37700300	0.95357700
O	0	4.05512900	0.60216300	0.70810700
C	0	9.10212400	2.06598400	-0.33756900
O	0	9.52104300	0.93023000	-0.55436200
C	0	5.60863400	1.47098400	2.27385300
C	0	5.25064200	0.54385300	3.25279600
C	0	6.59312400	2.42624600	2.53053100
C	0	5.88628200	0.57128100	4.48319800
H	0	4.50441800	-0.20879800	3.02738400
C	0	7.22677400	2.44676300	3.76130900
H	0	6.86237600	3.13649000	1.76031300
C	0	6.87461200	1.51749000	4.73556400
H	0	5.62308400	-0.15719700	5.24137000
H	0	8.00305900	3.17899600	3.95441500
H	0	7.37818000	1.52620700	5.69647800
C	0	9.66178200	2.91800300	0.72081000
C	0	10.56882500	2.35183000	1.61737800

C	0	9.26369300	4.24880900	0.86408900
C	0	11.06875400	3.11512500	2.66058600
H	0	10.86205600	1.31516700	1.49867700
C	0	9.77345000	5.00816900	1.90468100
H	0	8.55846800	4.67401300	0.16017100
C	0	10.67282500	4.44073900	2.80362300
H	0	11.76689600	2.67497700	3.36328800
H	0	9.47165700	6.04335200	2.01758800
H	0	11.06823700	5.03705200	3.61909700
C	0	-3.80308200	3.46513000	0.72508300
C	0	-4.94233900	2.47681400	0.89887400
H	0	-3.04610900	2.98297200	0.10044000
H	0	-4.18488800	4.32766700	0.16637000
C	0	-6.06736600	3.00200600	1.76982300
H	0	-4.57690000	1.53290600	1.30789300
C	0	-7.35938400	2.20579100	1.72567800
H	0	-5.69785900	3.00320300	2.79824700
H	0	-6.30016200	4.04192000	1.51502100
H	0	-7.16535900	1.16622600	1.44213700
C	0	-8.13890100	2.26161200	3.03014400
H	0	-8.13848900	3.29253700	3.40262500
H	0	-9.17959000	1.98912100	2.84138200
O	0	-5.49736400	2.20404100	-0.41456500
O	0	-8.12831000	2.80518500	0.63390300
C	0	-4.97790600	1.24263600	-1.14862800
O	0	-4.05173100	0.53493100	-0.76060700
C	0	-9.10680200	2.12248800	0.10612000
O	0	-9.50210200	1.05811600	0.58164600
C	0	-5.59869400	1.10018600	-2.46875600
C	0	-5.20984200	0.03143300	-3.27626100
C	0	-6.60671700	1.97105700	-2.88537800
C	0	-5.83796000	-0.16761000	-4.49454500
H	0	-4.44650300	-0.65274900	-2.92605900
C	0	-7.23286800	1.76471800	-4.10238700
H	0	-6.90145600	2.79285300	-2.24653200
C	0	-6.85023000	0.69368000	-4.90471800
H	0	-5.54940700	-1.00602600	-5.11792200
H	0	-8.02783500	2.43139100	-4.41775900
H	0	-7.34862300	0.52552700	-5.85359300
C	0	-9.69027100	2.71269600	-1.10562800
C	0	-10.58977600	1.94941000	-1.85110900
C	0	-9.31872700	3.98799100	-1.53745400
C	0	-11.10868400	2.45940000	-3.03079500
H	0	-10.86473700	0.95637100	-1.51466800
C	0	-9.84821200	4.49472200	-2.71295400
H	0	-8.61853400	4.56824200	-0.94878600

C	0	-10.74007300	3.72965400	-3.46025200
H	0	-11.79908500	1.86306200	-3.61603700
H	0	-9.56721400	5.48621000	-3.04977800
H	0	-11.15021500	4.12782300	-4.38234300
Cl	0	-1.83337400	0.65387700	1.60650600
Ti	0	-0.00292800	0.74656100	0.04742900
Cl	0	1.80179700	0.50272200	-1.53928400
C	0	0.02119900	2.79373700	-0.05405200
C	0	0.86125200	2.72383600	1.19679200
H	0	-0.94132200	3.28315400	0.08019900
H	0	0.54571900	3.16909500	-0.93254200
H	0	1.80917100	3.26090600	1.12862600
H	0	0.32464500	3.03427800	2.09376900
H	0	1.20602500	1.67641500	1.44016900
C	0	3.26863300	3.49935600	-2.71911300
H	0	2.90817200	2.55802100	-3.14096600
H	0	2.42135800	4.18791100	-2.67377500
H	0	4.00022900	3.93001000	-3.40762900
C	0	-3.17952800	3.91576100	2.03833600
H	0	-2.85395000	3.05970500	2.63430700
H	0	-2.30296900	4.54037000	1.84739500
H	0	-3.87512900	4.50946100	2.63718900
C	0	7.66158000	0.39615900	-4.05718300
H	0	6.61608400	0.54313900	-4.34216900
H	0	8.24306100	0.26995000	-4.97229500
H	0	7.71159400	-0.54387700	-3.49953300
C	0	-7.55097400	1.30866100	4.06311700
H	0	-6.50354500	1.53217200	4.28502500
H	0	-8.10839900	1.36769200	4.99982300
H	0	-7.59151600	0.27250500	3.71403300

$TiCl_2Et$-$MgCl_2$-2*SS*-HDDB

0 2

Mg	-1	-12.58265000	-2.74204100	0.81994100
Mg	-1	-9.43787800	-0.93107700	0.58863800
Mg	-1	-6.29595600	-2.75550200	0.43872700
Mg	-1	-3.15112700	-0.94463500	0.20745200
Mg	-1	-0.00923600	-2.76911400	0.05752600
Mg	-1	3.13557300	-0.95821600	-0.17378500
Mg	-1	6.27743900	-2.78274000	-0.32369100
Mg	-1	9.42216600	-0.97169200	-0.55494200
Mg	-1	12.56415500	-2.79599800	-0.70490800
Cl	0	-14.37777000	-3.99081000	1.02807000
Cl	0	-11.36982800	-1.19056100	2.19436600
Cl	0	-8.30512600	-2.97983700	1.65989700
Cl	0	-4.94992500	-1.34317500	1.88212100

Cl	0	-1.87440400	-2.98963600	1.31217800
Cl	0	1.16445500	-0.90761300	1.39442700
Cl	0	4.44056400	-2.84849300	1.16474400
Cl	0	7.51485100	-1.00121500	0.92186100
Cl	0	11.06569200	-2.09829400	1.01576100
Cl	0	-11.08027400	-2.13425600	-0.92919300
Cl	0	-7.53401800	-1.04037900	-0.88922500
Cl	0	-4.45970300	-2.91036900	-1.03977800
Cl	0	-1.17889800	-0.93408000	-1.35712200
Cl	0	1.85044600	-3.05725900	-1.19155000
Cl	0	4.93503200	-1.43885400	-1.83015600
Cl	0	8.28926100	-3.07369600	-1.52210100
Cl	0	11.35027300	-1.31244200	-2.15100400
Cl	0	14.35620200	-4.05765000	-0.85149800
O	0	9.25771400	0.98947900	-0.76464100
C	0	9.00297200	2.10489900	-0.31026500
O	0	8.27420000	2.97910400	-0.95002100
C	0	9.49682200	2.58131100	0.99178400
C	0	7.62691500	2.65818000	-2.22314900
C	0	10.09714000	1.67763700	1.86853900
C	0	9.35271500	3.92202000	1.35983300
C	0	8.63576500	2.29840600	-3.30612800
C	0	6.52054500	1.63666200	-2.03754400
H	0	7.19285600	3.62873000	-2.47603000
C	0	10.54457600	2.10953900	3.10679700
H	0	10.20487600	0.63399900	1.60063100
C	0	9.81276600	4.35081300	2.59400400
H	0	8.88569800	4.61583900	0.67192700
H	0	8.97069700	1.26610400	-3.18934200
H	0	9.51584800	2.93598500	-3.17141000
C	0	5.33601700	2.02486200	-1.16790500
H	0	6.11849700	1.41401600	-3.03009400
H	0	6.91783500	0.68855100	-1.67232200
C	0	10.40725800	3.44463300	3.46797100
H	0	10.99962800	1.40014600	3.78803400
H	0	9.71117500	5.39286500	2.87593100
O	0	5.74502000	1.97924800	0.22811000
H	0	4.56905300	1.26672200	-1.31308800
C	0	4.73009500	3.39395800	-1.41348200
H	0	10.76566500	3.78226400	4.43474500
C	0	5.08322900	1.22443600	1.07767500
H	0	5.46318000	4.17305700	-1.17626700
H	0	3.90636700	3.51659000	-0.70232800
O	0	4.13305300	0.51522700	0.75707600
C	0	5.55894200	1.31835900	2.46289800
C	0	5.10965400	0.36841200	3.38094700

C	0	6.45538500	2.31278000	2.85462300
C	0	5.56611700	0.41183800	4.68786800
H	0	4.43480700	-0.41282300	3.05208400
C	0	6.89896800	2.35644400	4.16607300
H	0	6.79927200	3.04024400	2.13103300
C	0	6.45777400	1.40484200	5.08022100
H	0	5.23340800	-0.33505600	5.39948900
H	0	7.59565500	3.12807600	4.47218600
H	0	6.81428700	1.43477000	6.10451500
O	0	-9.27694500	1.03833900	0.69046700
C	0	-9.01921800	2.12814400	0.17887000
O	0	-8.29238900	3.03387800	0.77531500
C	0	-9.50703100	2.53434400	-1.14861200
C	0	-7.64694900	2.77542900	2.06339200
C	0	-10.10807900	1.58512400	-1.97531300
C	0	-9.35677800	3.85185100	-1.59041600
C	0	-8.65545100	2.46614200	3.16234200
C	0	-6.53941200	1.74688200	1.92792500
H	0	-7.21460300	3.75767600	2.26956800
C	0	-10.55015500	1.94856000	-3.23714000
H	0	-10.22122900	0.55888000	-1.64901700
C	0	-9.81194400	4.21257800	-2.84799100
H	0	-8.88859900	4.58113300	-0.94099800
H	0	-8.97575100	1.42418000	3.10572400
H	0	-9.54410900	3.08188200	2.98829100
C	0	-5.35438300	2.09514000	1.04153900
H	0	-6.13980000	1.56966400	2.93068500
H	0	-6.93648400	0.78328800	1.60537600
C	0	-10.40699600	3.26093000	-3.67177100
H	0	-11.00553800	1.20374200	-3.87920900
H	0	-9.70543000	5.23677900	-3.18761200
O	0	-5.76211500	1.97946400	-0.35014400
H	0	-4.58559900	1.34700100	1.22514400
C	0	-4.75265400	3.47630200	1.22010100
H	0	-10.76107000	3.54514200	-4.65713700
C	0	-5.11370800	1.16529800	-1.15504500
H	0	-5.48894500	4.24059700	0.94738500
H	0	-3.93061100	3.56816200	0.50244200
O	0	-4.17623200	0.46000400	-0.79363300
C	0	-5.59110100	1.18555400	-2.54266700
C	0	-5.16475000	0.17181500	-3.40133500
C	0	-6.46630000	2.17367100	-2.99338700
C	0	-5.62258400	0.14573200	-4.70814900
H	0	-4.50692800	-0.60270200	-3.02516700
C	0	-6.91112800	2.14739100	-4.30495400
H	0	-6.79257200	2.95121100	-2.31508400

C	0	-6.49257600	1.13265900	-5.15992000
H	0	-5.30734900	-0.65005600	-5.37320800
H	0	-7.59068200	2.91456500	-4.65739300
H	0	-6.84971700	1.10859000	-6.18415700
Cl	0	-1.89068700	0.74196100	1.48506800
Ti	0	-0.01353300	0.79894000	-0.03644700
Cl	0	1.88304200	0.64350300	-1.54663900
C	0	-0.04354800	2.84019500	-0.18390200
C	0	0.89292800	2.81461800	0.99666600
H	0	-1.01736700	3.28320800	0.02006900
H	0	0.39629500	3.21742400	-1.10614300
H	0	0.46892800	3.25122100	1.90164500
H	0	1.16581500	1.76701200	1.32753300
H	0	1.87131100	3.25116000	0.78986500
C	0	-4.22325800	3.69810000	2.63001400
H	0	-3.72132300	4.66539200	2.70498700
H	0	-5.02233600	3.68491000	3.37695800
H	0	-3.50072700	2.92224300	2.89844500
C	0	4.19996300	3.54690400	-2.83212300
H	0	3.69088500	4.50595100	-2.95127500
H	0	4.99959700	3.50583200	-3.57738000
H	0	3.48312400	2.75453200	-3.06472400
C	0	-8.09032100	2.76903800	4.54375900
H	0	-7.78232400	3.81603200	4.63002100
H	0	-8.84389600	2.57977000	5.31079600
H	0	-7.22614500	2.14189300	4.77951300
C	0	8.06346400	2.51117800	-4.70146200
H	0	7.73016600	3.54402800	-4.84543700
H	0	8.82198100	2.29838000	-5.45736900
H	0	7.21543500	1.85075500	-4.90167200

$TiCl_2Et$-$MgCl_2$-2*RS*-HDDB-propene

0 2

Mg	-1	12.60691000	-2.85390400	-0.61767000
Mg	-1	9.45728900	-1.04464600	-0.44400700
Mg	-1	6.31554100	-2.87137600	-0.32443000
Mg	-1	3.16616000	-1.06205900	-0.15074300
Mg	-1	0.02400500	-2.88873100	-0.03128200
Mg	-1	-3.12542700	-1.07955300	0.14249600
Mg	-1	-6.26738200	-2.90620800	0.26199900
Mg	-1	-9.41672500	-1.09696200	0.43568100
Mg	-1	-12.55873500	-2.92365500	0.55521200
Cl	0	14.43071800	-4.06889200	-0.78014500
Cl	0	11.35883900	-1.40441000	-2.07706500
Cl	0	8.35493800	-3.18734200	-1.44022300
Cl	0	5.01916400	-1.45944800	-1.81565700

Cl	0	1.93186100	-3.19193500	-1.21403300
Cl	0	-1.17718400	-1.11188000	-1.41432600
Cl	0	-4.42835800	-2.90400700	-1.21962200
Cl	0	-7.43968700	-1.02045500	-0.89983600
Cl	0	-11.05137000	-2.21448700	-1.14177200
Cl	0	11.10557200	-2.18534700	1.10391100
Cl	0	7.48432100	-1.02055600	0.89992200
Cl	0	4.47507800	-2.90332100	1.16609200
Cl	0	1.16859900	-1.02881200	1.33046200
Cl	0	-1.85623000	-3.19542600	1.19986500
Cl	0	-4.96379300	-1.55873600	1.80928600
Cl	0	-8.30832200	-3.26590300	1.36229100
Cl	0	-11.31664100	-1.51070500	2.05758900
Cl	0	-14.38395900	-4.14040000	0.68485500
C	0	-4.04529300	3.13033600	1.64282300
C	0	-5.08326100	2.03483100	1.48281500
H	0	-3.22946400	2.92681100	0.94594700
H	0	-4.50382400	4.08143500	1.34649300
C	0	-6.26286000	2.21156400	2.42650800
H	0	-4.63949400	1.05130800	1.63504900
C	0	-7.54768200	1.50620800	2.04058400
H	0	-5.94488900	1.83709500	3.40228100
H	0	-6.49659600	3.27445800	2.55487500
H	0	-7.32964900	0.60286500	1.46133100
C	0	-8.43403500	1.18881400	3.23562400
H	0	-8.46046900	2.06276800	3.89653400
H	0	-9.45933700	1.02115300	2.89532400
O	0	-5.58405500	2.08240600	0.11788800
O	0	-8.22023900	2.44269800	1.13806800
C	0	-5.06351300	1.31050200	-0.81069700
O	0	-4.16009400	0.50495300	-0.60102200
C	0	-9.16492900	2.00568700	0.35146300
O	0	-9.60744100	0.85994200	0.40081100
C	0	-5.64275300	1.51571100	-2.14396200
C	0	-5.29253000	0.63038100	-3.16413700
C	0	-6.55966600	2.54017600	-2.38026800
C	0	-5.86644900	0.77147800	-4.41682700
H	0	-4.60681600	-0.18218500	-2.95255700
C	0	-7.13219200	2.67429700	-3.63434000
H	0	-6.82347400	3.21698000	-1.57850800
C	0	-6.78626000	1.78926400	-4.65084400
H	0	-5.61119000	0.07550600	-5.20765100
H	0	-7.85556100	3.46209900	-3.81371600
H	0	-7.24183300	1.88805100	-5.63048100
C	0	-9.65923800	2.98790600	-0.62349900
C	0	-10.54782100	2.55221600	-1.60686000

C	0	-9.21862100	4.31263000	-0.60494100
C	0	-10.98830000	3.44070400	-2.57507100
H	0	-10.87202100	1.51805900	-1.60868300
C	0	-9.66708700	5.19676400	-1.57292300
H	0	-8.52865800	4.63520600	0.16542500
C	0	-10.54894500	4.76036300	-2.55817800
H	0	-11.67378700	3.10374300	-3.34419400
H	0	-9.33174200	6.22772100	-1.56129200
H	0	-10.89707900	5.45482100	-3.31547100
C	0	3.97505100	3.23171200	-1.33397000
C	0	5.06252500	2.17490100	-1.26850100
H	0	3.17237500	2.92568100	-0.65958800
H	0	4.38855500	4.16933500	-0.94379500
C	0	6.24064900	2.47956200	-2.17791500
H	0	4.66316900	1.18927600	-1.51003800
C	0	7.52603000	1.73056700	-1.87883000
H	0	5.92722800	2.23242400	-3.19519100
H	0	6.46938600	3.55119900	-2.16742100
H	0	7.30996000	0.77621600	-1.38756500
C	0	8.39152600	1.51782900	-3.11183700
H	0	8.40372900	2.44216900	-3.70071900
H	0	9.42245600	1.32373000	-2.80589000
O	0	5.55597100	2.12644000	0.09646400
O	0	8.21916800	2.56702900	-0.89687900
C	0	5.01852900	1.29293600	0.96179700
O	0	4.10530400	0.52248400	0.67783500
C	0	9.17839900	2.04492000	-0.18267300
O	0	9.61541400	0.91151200	-0.37261300
C	0	5.60520200	1.37523300	2.30360500
C	0	5.21167500	0.44147200	3.26217600
C	0	6.58134000	2.32674900	2.60213400
C	0	5.80386000	0.45805800	4.51424100
H	0	4.47189300	-0.30748500	3.00562400
C	0	7.17169000	2.33645100	3.85439400
H	0	6.87776400	3.04280200	1.84740300
C	0	6.78422600	1.40029600	4.80860500
H	0	5.51223400	-0.27522800	5.25724200
H	0	7.94100900	3.06659400	4.08071600
H	0	7.25348200	1.40103100	5.78677700
C	0	9.69641200	2.91011900	0.88583100
C	0	10.59483400	2.36144500	1.80165900
C	0	9.26926800	4.23271800	1.01964300
C	0	11.05853100	3.13451000	2.85433600
H	0	10.90891500	1.33038500	1.68744300
C	0	9.74149000	5.00149900	2.07114400
H	0	8.57079800	4.64389600	0.30084400

C	0	10.63315300	4.45198000	2.98863800
H	0	11.75064800	2.70858700	3.57161100
H	0	9.41618400	6.03015600	2.17817900
H	0	10.99910000	5.05596800	3.81217700
Cl	0	1.88485300	0.43984800	-1.60569300
Ti	0	0.09439000	0.78211200	-0.02546400
Cl	0	-1.79219300	0.49583300	1.48478200
C	0	-1.43440000	2.42623900	-1.11830700
C	0	0.98368100	2.38724600	0.94014500
C	0	-0.56999900	2.35145900	-2.14757600
H	0	-1.26189300	3.10225600	-0.28639900
H	0	-2.38641000	1.90813600	-1.15340900
C	0	0.33822700	2.99553900	2.17618500
H	0	1.18653100	3.15167400	0.17629600
H	0	1.95705400	1.93578800	1.18946300
H	0	0.35018100	2.93204300	-2.10096400
C	0	-0.81555500	1.61189800	-3.41509900
H	0	0.96339700	3.78288600	2.61399500
H	0	-0.63459800	3.44210800	1.94718700
H	0	0.16594400	2.24212900	2.94856600
H	0	-0.00280000	0.91403200	-3.63222700
H	0	-1.75767500	1.06308900	-3.38969800
H	0	-0.85107300	2.33341900	-4.23926100
C	0	-3.49210000	3.21964800	3.05821100
H	0	-3.06216800	2.26462100	3.37019600
H	0	-2.70024000	3.97002600	3.11084100
H	0	-4.26059500	3.50681900	3.78045300
C	0	3.41776100	3.43950900	-2.73519400
H	0	3.04902000	2.49990200	-3.15440900
H	0	2.58390100	4.14631000	-2.71009900
H	0	4.16581900	3.85053500	-3.41813600
C	0	-7.94816000	-0.04439400	3.98657500
H	0	-6.92350500	0.06797100	4.35097800
H	0	-8.59090300	-0.24154700	4.84643300
H	0	-7.96567700	-0.93628300	3.35289900
C	0	7.88940200	0.34720700	-3.94768300
H	0	6.85323100	0.48178700	-4.27031300
H	0	8.50555500	0.22443500	-4.84025400
H	0	7.92912400	-0.59213900	-3.38754400

$TiCl_2Et$-$MgCl_2$-2*SS*-HDDB-propene

0 2

Mg	-1	12.58692300	-2.80205100	-0.85148600
Mg	-1	9.44379500	-0.99080700	-0.60087000
Mg	-1	6.30187200	-2.81463900	-0.44404800
Mg	-1	3.15844600	-1.00338100	-0.19341900

Mg	-1	0.01693100	-2.82747500	-0.03653000
Mg	-1	-3.12624300	-1.01588500	0.21401900
Mg	-1	-6.26829100	-2.83993500	0.37087500
Mg	-1	-9.41127800	-1.02872100	0.62152700
Mg	-1	-12.55331800	-2.85251800	0.77833800
Cl	0	14.38258100	-4.04753600	-1.07560100
Cl	0	11.36771800	-1.24548700	-2.21515400
Cl	0	8.30837000	-3.03862700	-1.67130800
Cl	0	4.96335300	-1.38515600	-1.87403200
Cl	0	1.89104000	-3.07320100	-1.28601200
Cl	0	-1.17950100	-1.01599200	-1.36522500
Cl	0	-4.44621300	-2.93352600	-1.11459700
Cl	0	-7.52630400	-1.07948800	-0.88251700
Cl	0	-11.06184300	-2.16098200	-0.94702900
Cl	0	11.09627700	-2.20174200	0.90731200
Cl	0	7.55282000	-1.10641300	0.88862500
Cl	0	4.47843400	-2.94859900	1.05139600
Cl	0	1.15884800	-0.96920800	1.32149400
Cl	0	-1.83193000	-3.11650100	1.24665900
Cl	0	-4.92616100	-1.47017000	1.86534400
Cl	0	-8.26976600	-3.10948900	1.60048800
Cl	0	-11.33896800	-1.36300800	2.21849700
Cl	0	-14.34630700	-4.11212500	0.93261800
O	0	-9.25986600	0.93635800	0.80320500
C	0	-9.01664600	2.04711100	0.33159000
O	0	-8.28933700	2.93503500	0.95411400
C	0	-9.52291300	2.50162100	-0.97334300
C	0	-7.63153000	2.63468600	2.22692700
C	0	-10.15793200	1.58941600	-1.81630900
C	0	-9.35534700	3.82892000	-1.37846300
C	0	-8.63137300	2.29080400	3.32307800
C	0	-6.52480400	1.61268400	2.04762600
H	0	-7.19703300	3.60954300	2.46165500
C	0	-10.61536800	1.99912400	-3.05846800
H	0	-10.28951000	0.55680400	-1.51777500
C	0	-9.82360700	4.23543300	-2.61711700
H	0	-8.86217700	4.52935100	-0.71602200
H	0	-8.96641900	1.25663700	3.22499000
H	0	-9.51294800	2.92569500	3.18514300
C	0	-5.35918700	1.98367400	1.14601700
H	0	-6.10350100	1.41683300	3.03791300
H	0	-6.92488400	0.65461600	1.71317300
C	0	-10.45190400	3.32042200	-3.45773200
H	0	-11.09876400	1.28315600	-3.71279000
H	0	-9.70218900	5.26691100	-2.92851200
O	0	-5.79158500	1.88579400	-0.24194000

H	0	-4.58198000	1.23815200	1.30225200
C	0	-4.76421300	3.36661600	1.33458700
H	0	-10.81724100	3.64067400	-4.42782500
C	0	-5.12504600	1.11729100	-1.07374200
H	0	-5.51598100	4.12973700	1.10402400
H	0	-3.96798300	3.48356200	0.59215300
O	0	-4.16068700	0.43295400	-0.73722000
C	0	-5.59204500	1.17160300	-2.46338400
C	0	-5.10448900	0.21905100	-3.35963800
C	0	-6.50884500	2.13514600	-2.88298000
C	0	-5.54294700	0.23050400	-4.67353100
H	0	-4.41647800	-0.54076600	-3.00756700
C	0	-6.93631200	2.14524500	-4.20049900
H	0	-6.88237400	2.86426200	-2.17617400
C	0	-6.45624900	1.19241500	-5.09348000
H	0	-5.18113700	-0.51874400	-5.36831800
H	0	-7.65109500	2.89151100	-4.52746900
H	0	-6.79994600	1.19592900	-6.12255200
O	0	9.28996300	0.98381100	-0.68704800
C	0	9.04475200	2.06131900	-0.14387600
O	0	8.30776400	2.98335100	-0.70260300
C	0	9.56019900	2.43728800	1.18247500
C	0	7.65052200	2.76943000	-1.99245900
C	0	10.20060100	1.47736500	1.96612100
C	0	9.39790000	3.73897500	1.66561800
C	0	8.65374700	2.50736000	-3.10827200
C	0	6.54886400	1.73149200	-1.88790400
H	0	7.21154300	3.75641500	-2.15868300
C	0	10.66828300	1.81351500	3.22638200
H	0	10.32867700	0.46351100	1.60801700
C	0	9.87683400	4.07242800	2.92180400
H	0	8.90008500	4.47722900	1.04936900
H	0	8.99165700	1.46975400	-3.08211500
H	0	9.53307800	3.13350000	-2.92388400
C	0	5.36264200	2.04280900	-0.99069900
H	0	6.14828100	1.58429600	-2.89513100
H	0	6.95177100	0.76061300	-1.59627400
C	0	10.51025100	3.10955300	3.70296300
H	0	11.15512100	1.05990100	3.83422000
H	0	9.75897700	5.08411000	3.29363800
O	0	5.77687800	1.89829700	0.39579500
H	0	4.60563500	1.28704100	-1.19037500
C	0	4.73629000	3.41773900	-1.13189900
H	0	10.88312800	3.37239500	4.68731700
C	0	5.10477700	1.09416100	1.19071900
H	0	5.45972600	4.18779000	-0.84132200

H	0	3.91427300	3.47465200	-0.41053700
O	0	4.14663800	0.42267100	0.81658400
C	0	5.57695200	1.08226400	2.57935100
C	0	5.08079400	0.10021600	3.43731300
C	0	6.51617900	2.00888600	3.03218100
C	0	5.53464800	0.04248700	4.74470300
H	0	4.36868000	-0.62468800	3.06183700
C	0	6.95961400	1.94999300	4.34276300
H	0	6.89415700	2.76362600	2.35541300
C	0	6.47209100	0.96543800	5.19666500
H	0	5.16357300	-0.72885900	5.40971700
H	0	7.69214800	2.66709700	4.69478700
H	0	6.82759800	0.91489000	6.22050000
Cl	0	1.91995600	0.64721500	-1.53181800
Ti	0	0.08121400	0.89129900	0.03085700
Cl	0	-1.84538400	0.63602700	1.51830600
C	0	-1.48030200	2.54608000	-1.00955800
C	0	1.00179600	2.46739800	1.01403000
C	0	-0.62631800	2.47887000	-2.04851000
H	0	-1.30326100	3.21492100	-0.17264700
H	0	-2.42829600	2.01953800	-1.03313400
C	0	0.35175000	3.08824700	2.24207100
H	0	1.24171500	3.22166000	0.25007800
H	0	1.95920900	1.98884500	1.27495000
H	0	0.28994600	3.06649700	-2.01057200
C	0	-0.88083700	1.73940700	-3.31503300
H	0	0.98482200	3.86625100	2.68529700
H	0	-0.61317300	3.55019900	2.00691500
H	0	0.16274400	2.33825400	3.01399400
H	0	-0.06367600	1.05033900	-3.54396900
H	0	-1.81732500	1.18140700	-3.27930600
H	0	-0.93326700	2.46208400	-4.13729900
C	0	-4.18780400	3.56095100	2.73010700
H	0	-3.69510900	4.53252900	2.81136700
H	0	-4.95981000	3.51969200	3.50405400
H	0	-3.44660200	2.78837500	2.95310700
C	0	4.19959900	3.66800800	-2.53427500
H	0	3.49550600	2.88256400	-2.82368600
H	0	3.67573700	4.62567200	-2.58063900
H	0	4.99680300	3.69510700	-3.28292600
C	0	-8.04796500	2.52630600	4.71008800
H	0	-7.71234300	3.56105600	4.83357400
H	0	-8.80071900	2.32751300	5.47548700
H	0	-7.19916400	1.86832200	4.91477500
C	0	8.07319300	2.83867600	-4.47668900
H	0	7.73639400	3.87915900	-4.52805400

H	0	8.82774800	2.69496700	-5.25260800
H	0	7.22561600	2.19565300	-4.72885000

$TiCl_2Et-MgCl_2$-2*RS*-HDDB-TS-*si*

0 2

Mg	-1	-12.56379300	-2.94549400	0.71680700
Mg	-1	-9.42495400	-1.11956900	0.52482000
Mg	-1	-6.27684700	-2.92992800	0.33943200
Mg	-1	-3.13811400	-1.10388400	0.14742200
Mg	-1	0.01010600	-2.91428500	-0.03794300
Mg	-1	3.14883000	-1.08832800	-0.22996400
Mg	-1	6.29701200	-2.89863000	-0.41535600
Mg	-1	9.43578000	-1.07270800	-0.60732200
Mg	-1	12.58393700	-2.88308100	-0.79273500
Cl	0	-14.38778400	-4.15681100	0.90616100
Cl	0	-11.30837700	-1.48989300	2.16973100
Cl	0	-8.29324300	-3.25044000	1.50117400
Cl	0	-4.97231700	-1.50611700	1.81836900
Cl	0	-1.87858800	-3.21522000	1.19318300
Cl	0	1.18867700	-1.15954600	1.31762600
Cl	0	4.46987200	-2.98972600	1.06063800
Cl	0	7.50970300	-1.07920900	0.81146000
Cl	0	11.10441400	-2.19685100	0.93996700
Cl	0	-11.07686300	-2.28298700	-1.01196400
Cl	0	-7.48112800	-1.09281700	-0.86300200
Cl	0	-4.45711400	-2.96392100	-1.16537400
Cl	0	-1.15154600	-1.11226600	-1.37328000
Cl	0	1.88093500	-3.18246000	-1.31367900
Cl	0	5.00242000	-1.41966400	-1.86708400
Cl	0	8.30578000	-3.18548100	-1.60229300
Cl	0	11.32913700	-1.43770300	-2.24701800
Cl	0	14.40110500	-4.10786700	-0.95720600
C	0	3.90100700	3.22516300	-1.07972100
C	0	5.02363500	2.20253600	-1.13444000
H	0	3.53160400	3.35804700	-2.10245000
H	0	3.07752600	2.77226800	-0.52525900
C	0	6.14832500	2.59038900	-2.07801300
H	0	4.62884300	1.23283200	-1.43724900
C	0	7.44478900	1.82256800	-1.90343100
H	0	5.77216500	2.41959700	-3.09052000
H	0	6.38463700	3.65567600	-2.00258600
H	0	7.25136600	0.83417400	-1.47444500
C	0	8.24952000	1.69901400	-3.18769000
H	0	8.24016000	2.66310700	-3.70903700
H	0	9.29138200	1.47955400	-2.94224100
O	0	5.57936600	2.04601700	0.19838100

O	0	8.18491200	2.58504200	-0.89587000
C	0	5.03807900	1.17256300	1.02170900
O	0	4.09810300	0.44674200	0.70421800
C	0	9.15701800	2.00001800	-0.25185500
O	0	9.57490800	0.88225300	-0.54902400
C	0	5.64063500	1.16077200	2.35734200
C	0	5.22549600	0.18862300	3.26763600
C	0	6.64745700	2.06565300	2.69728700
C	0	5.82721200	0.12039600	4.51341200
H	0	4.46182500	-0.52322600	2.97750900
C	0	7.24630800	1.99051800	3.94298300
H	0	6.96386000	2.80954100	1.97828200
C	0	6.83742800	1.01615900	4.84900600
H	0	5.51945400	-0.64313000	5.21845900
H	0	8.03939200	2.68357900	4.20128300
H	0	7.31379700	0.95013200	5.82148600
C	0	9.70871800	2.76964700	0.87094000
C	0	10.62292300	2.14145700	1.71770000
C	0	9.29342200	4.07874500	1.12433600
C	0	11.11439400	2.82169900	2.82070800
H	0	10.92797500	1.12148500	1.51367500
C	0	9.79406400	4.75469000	2.22513700
H	0	8.58119300	4.55145800	0.45876700
C	0	10.70167700	4.12567100	3.07348100
H	0	11.81821300	2.33320500	3.48469600
H	0	9.47853600	5.77253100	2.42481700
H	0	11.08984900	4.65683200	3.93612400
C	0	-4.03380600	3.17260100	1.27188300
C	0	-5.08765800	2.07970200	1.23650200
H	0	-3.70206400	3.26855000	2.31180000
H	0	-3.16746100	2.80771800	0.71851900
C	0	-6.23053200	2.32818700	2.20906300
H	0	-4.63272900	1.11733900	1.46584000
C	0	-7.52572400	1.59714000	1.92030300
H	0	-5.86770800	2.01821200	3.19279000
H	0	-6.46856600	3.39365700	2.28037700
H	0	-7.32407500	0.65448500	1.40104200
C	0	-8.37207000	1.36468100	3.16271800
H	0	-8.37025400	2.27809500	3.76848000
H	0	-9.40916900	1.18413600	2.86821000
O	0	-5.62455800	1.99461700	-0.10938100
O	0	-8.22614800	2.46540600	0.97242000
C	0	-5.05930700	1.18987300	-0.98707200
O	0	-4.11703000	0.45075300	-0.71928900
C	0	-9.18950100	1.96749500	0.24696400
O	0	-9.63279200	0.83187900	0.40577400

C	0	-5.65432400	1.27145000	-2.32618500
C	0	-5.25540800	0.34568100	-3.29023700
C	0	-6.63739400	2.21780100	-2.61851200
C	0	-5.84810700	0.36603800	-4.54214200
H	0	-4.51209500	-0.40107400	-3.03695200
C	0	-7.22690300	2.23233200	-3.87136200
H	0	-6.94223400	2.92534200	-1.85891500
C	0	-6.83339600	1.30481400	-4.83143900
H	0	-5.55306000	-0.36185600	-5.28913600
H	0	-8.00052000	2.95927100	-4.09332900
H	0	-7.30220200	1.30895200	-5.80981800
C	0	-9.70294000	2.86399000	-0.79765000
C	0	-10.62027600	2.35054900	-1.71498100
C	0	-9.25005500	4.18005600	-0.91052800
C	0	-11.07849000	3.15283700	-2.74808400
H	0	-10.95214000	1.32348100	-1.61619900
C	0	-9.71612400	4.97773200	-1.94309200
H	0	-8.53552000	4.56265400	-0.19163400
C	0	-10.62754900	4.46376700	-2.86180400
H	0	-11.78607800	2.75494800	-3.46639300
H	0	-9.37061200	6.00130800	-2.03451600
H	0	-10.98887800	5.09049600	-3.67026200
Cl	0	-1.85095000	0.50535400	1.51083000
Cl	0	1.84561500	0.52703000	-1.58546900
Ti	0	0.04369100	0.74861400	-0.00295200
C	0	1.13048000	1.95943400	1.48305900
C	0	-0.70124800	2.52659200	-0.93509200
C	0	0.59362600	3.05454000	0.81420300
H	0	2.14713600	1.64485400	1.26923500
H	0	0.72170800	1.66311300	2.44462800
C	0	-0.13021800	3.47844600	-1.95826300
H	0	-1.63184400	2.89695300	-0.50435700
H	0	-0.98097700	1.57813600	-1.47197500
H	0	1.22331700	3.53804100	0.07410800
C	0	-0.42248100	3.93560000	1.47507200
H	0	-0.81562700	3.60357600	-2.80359900
H	0	0.02590200	4.47314800	-1.52857000
H	0	0.82209300	3.11742700	-2.35438800
H	0	-0.95607900	4.57533600	0.76987800
H	0	-1.14228400	3.34555600	2.04480900
H	0	0.11045500	4.58557700	2.17766400
C	0	-4.48500400	4.51711800	0.72104400
H	0	-3.66912900	5.24254200	0.77300300
H	0	-4.79019000	4.43149300	-0.32480400
H	0	-5.32600400	4.93654300	1.28013600
C	0	-7.86712400	0.17632500	3.97141000

H	0	-6.82748400	0.29998100	4.28636200
H	0	-8.47593500	0.04044600	4.86710200
H	0	-7.91686500	-0.75356400	3.39647900
C	0	4.27860200	4.56232800	-0.45980100
H	0	3.41042900	5.22581000	-0.42887300
H	0	4.63659000	4.43454800	0.56476600
H	0	5.05853200	5.07922000	-1.02528300
C	0	7.70185500	0.59176600	-4.07948700
H	0	6.65315200	0.75244000	-4.34465600
H	0	8.27505500	0.52978600	-5.00635800
H	0	7.76094400	-0.38344400	-3.58656600

$TiCl_2Et$-$MgCl_2$-2*RS*-HDDB-TS-*re*

0 2

Mg	-1	-12.58197800	-2.97176800	0.43294100
Mg	-1	-9.44048900	-1.14517200	0.30151600
Mg	-1	-6.28797600	-2.95471700	0.20210400
Mg	-1	-3.14648700	-1.12812100	0.07068000
Mg	-1	0.00602500	-2.93766600	-0.02873100
Mg	-1	3.14751400	-1.11106900	-0.16015200
Mg	-1	6.30002700	-2.92061600	-0.25956400
Mg	-1	9.44151700	-1.09401900	-0.39098300
Mg	-1	12.59403000	-2.90356400	-0.49040800
Cl	0	-14.40658000	-4.19026400	0.55838800
Cl	0	-11.34355400	-1.56015400	1.93293400
Cl	0	-8.35446200	-3.33727900	1.23642100
Cl	0	-5.00073800	-1.62648500	1.76499900
Cl	0	-1.89211000	-3.19382200	1.19350400
Cl	0	1.14631400	-1.07107200	1.27547400
Cl	0	4.45456200	-2.90453200	1.22274300
Cl	0	7.44291600	-1.01325800	0.90559700
Cl	0	11.06570500	-2.22009300	1.19670500
Cl	0	-11.07523100	-2.25696600	-1.26671800
Cl	0	-7.43731700	-1.06688300	-0.98651300
Cl	0	-4.43012100	-2.95452600	-1.26610700
Cl	0	-1.15418000	-1.21420800	-1.46572500
Cl	0	1.92094900	-3.27721400	-1.19860800
Cl	0	5.00770500	-1.58561700	-1.81371000
Cl	0	8.35709300	-3.26730400	-1.33224000
Cl	0	11.35772500	-1.48712800	-1.99626100
Cl	0	14.43294300	-4.09991600	-0.61874500
C	0	4.06901000	3.02582300	-1.59418300
C	0	5.18527600	2.01253000	-1.43680500
H	0	3.25033700	2.70099900	-0.94863100
H	0	4.42794200	3.99203100	-1.21908700
C	0	6.38856800	2.31478200	-2.31148100

H	0	4.82818800	1.00310300	-1.64629900
C	0	7.67137000	1.59779700	-1.93500600
H	0	6.11798400	2.02975700	-3.33093700
H	0	6.59629600	3.39050500	-2.32613900
H	0	7.44190700	0.67086800	-1.39842300
C	0	8.57880200	1.32526000	-3.12484200
H	0	8.62171600	2.22406900	-3.75051700
H	0	9.59636000	1.13574100	-2.77340700
O	0	5.62704300	2.04217100	-0.05325900
O	0	8.32603200	2.48927000	-0.97720100
C	0	5.03756700	1.27799500	0.84232200
O	0	4.11236000	0.51593400	0.57472600
C	0	9.24901700	2.00353800	-0.19138100
O	0	9.68031800	0.85704900	-0.29065500
C	0	5.57972600	1.43475700	2.19686200
C	0	5.14319800	0.56475000	3.19596500
C	0	6.55700100	2.39273800	2.47057200
C	0	5.69475800	0.65050600	4.46385200
H	0	4.40347600	-0.19112900	2.96017400
C	0	7.10590800	2.47221800	3.73909100
H	0	6.88778100	3.05857200	1.68473800
C	0	6.67618800	1.59895000	4.73399400
H	0	5.37097900	-0.03449100	5.23894800
H	0	7.87655700	3.20656800	3.94659200
H	0	7.11340200	1.65337900	5.72541400
C	0	9.73044200	2.92736700	0.84456700
C	0	10.58812000	2.42642600	1.82448200
C	0	9.30977800	4.25807200	0.88521000
C	0	11.01865500	3.25625600	2.84761200
H	0	10.89553200	1.38803300	1.78044400
C	0	9.74809300	5.08322200	1.90847500
H	0	8.64336100	4.63189400	0.11720000
C	0	10.59982000	4.58202400	2.88933700
H	0	11.67990700	2.86857300	3.61404800
H	0	9.42808800	6.11849300	1.94358500
H	0	10.93988000	5.23042300	3.68988900
C	0	-4.10215000	3.04379400	1.81909100
C	0	-5.09255700	1.92256600	1.56102100
H	0	-3.29041800	2.95238700	1.09617800
H	0	-4.60650100	3.99881700	1.62801100
C	0	-6.27977600	1.98964000	2.51235400
H	0	-4.60987400	0.95030000	1.64576300
C	0	-7.56367600	1.32398900	2.06270600
H	0	-5.96154900	1.52081800	3.44629100
H	0	-6.52138100	3.03271300	2.74677100
H	0	-7.34915000	0.45387700	1.43369000

C	0	-8.47541400	0.95596400	3.22400700
H	0	-8.51415600	1.80258500	3.91910100
H	0	-9.49347100	0.80809000	2.85366200
O	0	-5.57849000	2.05360200	0.19765700
O	0	-8.21269700	2.32551800	1.21311400
C	0	-5.09798700	1.30472000	-0.77511500
O	0	-4.24023500	0.44250300	-0.62351200
C	0	-9.15231700	1.95846000	0.38653800
O	0	-9.60249000	0.81569000	0.33708800
C	0	-5.68130300	1.61457200	-2.08801100
C	0	-5.37466000	0.78114400	-3.16390600
C	0	-6.56051400	2.68500800	-2.25242400
C	0	-5.95490700	1.01834000	-4.39900000
H	0	-4.71308200	-0.06345700	-3.01001600
C	0	-7.14156800	2.91467200	-3.48888800
H	0	-6.78931200	3.32268300	-1.40904100
C	0	-6.83951400	2.08038100	-4.56069100
H	0	-5.73147600	0.36368100	-5.23354300
H	0	-7.83717200	3.73768100	-3.61135500
H	0	-7.30089500	2.25444500	-5.52707400
C	0	-9.63479300	3.02173200	-0.50675300
C	0	-10.52244400	2.67687100	-1.52598800
C	0	-9.18714000	4.33742500	-0.37469800
C	0	-10.95545900	3.64675200	-2.41633700
H	0	-10.85220000	1.64853300	-1.61586800
C	0	-9.62732800	5.30310000	-1.26544400
H	0	-8.49804200	4.58935500	0.42229600
C	0	-10.50884500	4.95761200	-2.28638400
H	0	-11.64066300	3.38027000	-3.21288000
H	0	-9.28626700	6.32738600	-1.16524000
H	0	-10.85069200	5.71601100	-2.98274900
Cl	0	-1.84861800	0.53374600	1.31329300
Ti	0	0.04880000	0.73441700	-0.23141500
Cl	0	1.89376200	0.38554100	-1.70359500
C	0	0.94444000	2.13018100	1.22443200
C	0	-0.67160100	2.38041800	-1.38881600
C	0	0.19602400	3.10157300	0.56644400
H	0	2.02436100	2.10301800	1.10383400
H	0	0.54323700	1.66878100	2.12087300
C	0	-1.99188500	3.10810900	-1.39842800
H	0	-0.83951800	1.38252000	-1.87815000
H	0	0.08880700	2.86596200	-2.00464300
C	0	0.83327900	4.29350200	-0.07885800
H	0	-0.81857000	3.25447100	0.92686500
H	0	-2.37745900	3.21897900	-2.41797800
H	0	-2.73545400	2.55755800	-0.82303700

H	0	-1.90811100	4.11443300	-0.97661100
H	0	0.17814100	4.80120200	-0.78772100
H	0	1.07385700	5.00459700	0.71932600
H	0	1.76629200	4.03224000	-0.57773800
C	0	-3.52433000	3.01094300	3.22720900
H	0	-2.75302800	3.77712800	3.33929600
H	0	-4.28414400	3.20115200	3.98959700
H	0	-3.06165600	2.04273400	3.43482400
C	0	3.56469400	3.15844200	-3.02353800
H	0	2.69485300	3.81963800	-3.06035000
H	0	4.32285400	3.58310100	-3.68667400
H	0	3.25678500	2.18923600	-3.42281300
C	0	-8.01578100	-0.30907300	3.93727800
H	0	-7.00308600	-0.21618700	4.33836700
H	0	-8.68675700	-0.53717700	4.76750300
H	0	-8.01782500	-1.17373400	3.26763000
C	0	8.09597300	0.12551200	-3.93019500
H	0	7.07788700	0.25962000	-4.30599000
H	0	8.75016800	-0.04472700	-4.78702800
H	0	8.09690700	-0.79044600	-3.33096100

$TiCl_2Et$-$MgCl_2$-2*SS*-HDDB-TS-*si*

0 2

Mg	-1	12.57366500	-2.86635500	-0.80659200
Mg	-1	9.43190800	-1.04749800	-0.59749500
Mg	-1	6.28842000	-2.86491900	-0.40196900
Mg	-1	3.14663800	-1.04608000	-0.19285300
Mg	-1	0.00317200	-2.86350200	0.00265500
Mg	-1	-3.13862000	-1.04465600	0.21175900
Mg	-1	-6.28207200	-2.86210700	0.40727600
Mg	-1	-9.42380600	-1.04323500	0.61640500
Mg	-1	-12.56731500	-2.86064300	0.81189200
Cl	0	14.37030600	-4.11504800	-1.00401600
Cl	0	11.35919300	-1.33431400	-2.20246900
Cl	0	8.29612000	-3.11342300	-1.62275600
Cl	0	4.95003500	-1.46295900	-1.86078200
Cl	0	1.87875000	-3.12058100	-1.25509300
Cl	0	-1.16474500	-1.08177900	-1.33370700
Cl	0	-4.45913600	-2.99105800	-1.07358100
Cl	0	-7.53221000	-1.11571500	-0.87856800
Cl	0	-11.06703800	-2.22486700	-0.92539000
Cl	0	11.07763300	-2.23220200	0.93641200
Cl	0	7.53522400	-1.11796200	0.88862200
Cl	0	4.47029200	-2.98826700	1.09262700
Cl	0	1.14391900	-1.04227400	1.33766500
Cl	0	-1.85384500	-3.11903900	1.29507300

Cl	0	-4.94711700	-1.44969500	1.86756000
Cl	0	-8.28585100	-3.10786700	1.63675100
Cl	0	-11.35447200	-1.33286700	2.21448200
Cl	0	-14.36638100	-4.10660000	1.00333500
O	0	-9.27835900	0.92923100	0.73968600
C	0	-9.02855100	2.02035000	0.22736500
O	0	-8.29817300	2.92771800	0.81801800
C	0	-9.52884700	2.42833300	-1.09516500
C	0	-7.64478600	2.67254400	2.10233700
C	0	-10.13331600	1.47932100	-1.91952400
C	0	-9.38582100	3.74734200	-1.53527300
C	0	-8.64736900	2.36574500	3.20739400
C	0	-6.53794900	1.64368300	1.96417100
H	0	-7.21149400	3.65550500	2.30373400
C	0	-10.58531900	1.84408000	-3.17750200
H	0	-10.24173800	0.45205500	-1.59518100
C	0	-9.85029200	4.10920400	-2.78913900
H	0	-8.91561800	4.47690300	-0.88763300
H	0	-8.97069800	1.32465500	3.15182000
H	0	-9.53525300	2.98403800	3.03851800
C	0	-5.35512400	1.98828300	1.07436700
H	0	-6.13483900	1.46733200	2.96571800
H	0	-6.93541500	0.67959100	1.64374000
C	0	-10.44841700	3.15754500	-3.61079200
H	0	-11.04337000	1.09909700	-3.81749100
H	0	-9.74909000	5.13440000	-3.12737200
O	0	-5.76793000	1.87534900	-0.31631200
H	0	-4.59031100	1.23560000	1.25465100
C	0	-4.74398200	3.36575200	1.25156000
H	0	-10.81005600	3.44274900	-4.59312400
C	0	-5.10362600	1.08186100	-1.12794700
H	0	-5.47456400	4.13532800	0.97840300
H	0	-3.92193600	3.45036000	0.53229900
O	0	-4.14721700	0.39772200	-0.77017500
C	0	-5.57546600	1.10286100	-2.51631200
C	0	-5.09917000	0.12356200	-3.38888700
C	0	-6.49186600	2.05833200	-2.95600700
C	0	-5.54949500	0.09827700	-4.69839800
H	0	-4.40764500	-0.62587900	-3.02265200
C	0	-6.92965000	2.03280200	-4.26969600
H	0	-6.85635700	2.80927800	-2.26737000
C	0	-6.46189500	1.05158300	-5.13845900
H	0	-5.19542600	-0.67152400	-5.37438900
H	0	-7.64259600	2.77347900	-4.61279200
H	0	-6.81389800	1.02722100	-6.16446300
O	0	9.28973400	0.92507600	-0.71243700

C	0	9.03152400	2.01577700	-0.20340600
O	0	8.29953700	2.91818700	-0.79940100
C	0	9.52401700	2.42867000	1.12042000
C	0	7.65083900	2.65496700	-2.08454400
C	0	10.11944900	1.48158100	1.95349000
C	0	9.38380300	3.75061100	1.55229700
C	0	8.65697600	2.34475100	-3.18551600
C	0	6.54590300	1.62424900	-1.94352200
H	0	7.21601800	3.63592000	-2.29215100
C	0	10.56553400	1.85155000	3.21208000
H	0	10.22448400	0.45209600	1.63499100
C	0	9.84366400	4.11784000	2.80625700
H	0	8.92031700	4.47835400	0.89777800
H	0	8.97864200	1.30329900	-3.12732700
H	0	9.54539600	2.96183800	-3.01509100
C	0	5.35437700	1.97688000	-1.06828900
H	0	6.15213400	1.43346800	-2.94611900
H	0	6.94388800	0.66564800	-1.60752300
C	0	10.43270400	3.16822100	3.63672600
H	0	11.01616700	1.10828700	3.85928300
H	0	9.74573500	5.14555300	3.13782900
O	0	5.75580500	1.88971300	0.32678600
H	0	4.59310000	1.21931800	-1.24295800
C	0	4.73981000	3.34880600	-1.27420000
H	0	10.79027200	3.45744400	4.61937500
C	0	5.10719700	1.08674500	1.14383400
H	0	5.46115200	4.12562200	-0.99680900
H	0	3.90322400	3.43660000	-0.57258900
O	0	4.17044600	0.37596900	0.79391700
C	0	5.58453800	1.12923100	2.53132900
C	0	5.14802500	0.13580800	3.40849300
C	0	6.47041500	2.11615800	2.96363800
C	0	5.60563900	0.12942500	4.71578500
H	0	4.48277100	-0.63916000	3.04652800
C	0	6.91545300	2.10939200	4.27538100
H	0	6.80598400	2.87647400	2.27046500
C	0	6.48610000	1.11528300	5.14912600
H	0	5.28262300	-0.65091500	5.39527600
H	0	7.60440900	2.87482100	4.61310800
H	0	6.84373200	1.10603400	6.17344700
Cl	0	1.92135800	0.67160500	-1.47527700
Ti	0	-0.02675900	0.83503400	-0.00666300
Cl	0	-1.89789700	0.67470800	1.51336500
C	0	-1.12874800	2.03692800	-1.49858600
C	0	0.73247900	2.60678300	0.91351600
C	0	-0.61949700	3.13353900	-0.81372300

H	0	-2.13870700	1.69813100	-1.29052300
H	0	-0.70579100	1.75742000	-2.45938300
C	0	0.18770900	3.57988400	1.93039400
H	0	1.66386500	2.94881900	0.45709100
H	0	0.99773900	1.65981800	1.46250600
H	0	-1.25858900	3.57920400	-0.05658100
C	0	0.38747000	4.04220100	-1.45184200
H	0	0.88056700	3.70048600	2.77041100
H	0	0.04679400	4.57307800	1.49235800
H	0	-0.76959000	3.24189300	2.33561400
H	0	0.88760600	4.69526000	-0.73466400
H	0	1.13781300	3.46958600	-2.00051500
H	0	-0.14223000	4.67783500	-2.16974200
C	0	-4.21185200	3.58519400	2.66078900
H	0	-3.69521100	4.54509600	2.73326400
H	0	-5.01192700	3.58711400	3.40664000
H	0	-3.50270900	2.79844500	2.93324900
C	0	4.23355700	3.54914500	-2.69562400
H	0	3.53843400	2.75200900	-2.97429900
H	0	3.70878900	4.50304000	-2.78739000
H	0	5.04756900	3.55246600	-3.42612400
C	0	-8.07373800	2.66661800	4.58573100
H	0	-7.76072300	3.71231600	4.67009100
H	0	-8.82423300	2.48114100	5.35669300
H	0	-7.21132100	2.03562700	4.81756900
C	0	8.08810200	2.64392800	-4.56617500
H	0	7.77971100	3.69069000	-4.65435500
H	0	8.83959200	2.45288000	-5.33481400
H	0	7.22346500	2.01593900	-4.79805400

$TiCl_2Et$-$MgCl_2$-2*SS*-HDDB-TS-*re*

0 2

Mg	-1	-12.57333400	-2.93247600	0.55586100
Mg	-1	-9.43380900	-1.10066600	0.45321000
Mg	-1	-6.28503600	-2.90215600	0.20312200
Mg	-1	-3.14545400	-1.07044400	0.10049300
Mg	-1	0.00328800	-2.87198600	-0.14961300
Mg	-1	3.14285000	-1.04024100	-0.25227500
Mg	-1	6.29156600	-2.84183000	-0.50236500
Mg	-1	9.43104600	-1.00993900	-0.60495800
Mg	-1	12.57988400	-2.81130600	-0.85509400
Cl	0	-14.35903900	-4.20879000	0.64883500
Cl	0	-11.35757900	-1.51206500	2.05767100
Cl	0	-8.31006700	-3.23038900	1.36679100
Cl	0	-4.93905800	-1.62732800	1.76290500
Cl	0	-1.87153300	-3.18099700	1.09307300

Cl	0	1.14910300	-1.10597800	1.24214300
Cl	0	4.45517200	-3.00929000	0.96776500
Cl	0	7.53973500	-1.16561200	0.87512900
Cl	0	11.08255400	-2.22250100	0.90145600
Cl	0	-11.08903800	-2.15069000	-1.14502100
Cl	0	-7.51538700	-1.07285900	-0.99534200
Cl	0	-4.45452800	-2.90742300	-1.29412700
Cl	0	-1.15683300	-1.04766700	-1.45241100
Cl	0	1.87573200	-3.08676800	-1.42186100
Cl	0	4.96669200	-1.37971200	-1.91530400
Cl	0	8.30596300	-3.03438000	-1.72398700
Cl	0	11.36668500	-1.24649400	-2.21407900
Cl	0	14.37825400	-4.05342900	-1.07690400
O	0	9.30881200	0.95831300	-0.65380200
C	0	9.06001600	2.04808400	-0.13968800
O	0	8.34943800	2.96357600	-0.74140000
C	0	9.53957100	2.44032500	1.19426600
C	0	7.71191400	2.70487300	-2.03365900
C	0	10.17764200	1.49241700	1.99476600
C	0	9.34028700	3.73933000	1.67016200
C	0	8.72246600	2.38306200	-3.12674600
C	0	6.58982900	1.69273600	-1.89782000
H	0	7.29175800	3.69042200	-2.24833200
C	0	10.60771700	1.83936100	3.26543000
H	0	10.33248500	0.48153300	1.63818700
C	0	9.77939200	4.08253000	2.93832200
H	0	8.84423000	4.46701900	1.04003500
H	0	9.04394300	1.34234200	-3.06017700
H	0	9.61016500	3.00148000	-2.95708200
C	0	5.43625600	2.04472000	-0.97156000
H	0	6.16167300	1.55286200	-2.89471700
H	0	6.97207100	0.71205100	-1.61177100
C	0	10.41178400	3.13260000	3.73610100
H	0	11.09426900	1.09658900	3.88671200
H	0	9.63170100	5.09154500	3.30661000
O	0	5.84460200	1.81704300	0.40631400
H	0	4.62154300	1.35629100	-1.18994700
C	0	4.92409100	3.47058500	-1.04947900
H	0	10.75451500	3.40357800	4.72919800
C	0	5.12855800	1.01621500	1.16776200
H	0	5.72788100	4.16889500	-0.79200400
H	0	4.16307000	3.58500500	-0.27164800
O	0	4.15179900	0.39279400	0.75821100
C	0	5.56669200	0.95707900	2.56646000
C	0	5.01583400	-0.02606300	3.38945200
C	0	6.51955600	1.84520600	3.06588200

C	0	5.42876100	-0.12400000	4.70789300
H	0	4.29339900	-0.72157000	2.97969100
C	0	6.92083000	1.74707500	4.38775300
H	0	6.94154000	2.60126000	2.41716500
C	0	6.37907100	0.76101400	5.20647300
H	0	5.01540200	-0.89727700	5.34513500
H	0	7.66302000	2.43502500	4.77595800
H	0	6.70216000	0.67926400	6.23898800
O	0	-9.32132700	0.84437400	0.72467500
C	0	-9.05112800	2.00131300	0.40690500
O	0	-8.35388200	2.79648400	1.17214900
C	0	-9.49114200	2.60987200	-0.85734600
C	0	-7.74021000	2.30975000	2.41028900
C	0	-10.07293900	1.80000400	-1.83320600
C	0	-9.31297700	3.97548700	-1.09334900
C	0	-8.76554100	1.79372600	3.41144100
C	0	-6.61072800	1.34181500	2.11014100
H	0	-7.32958900	3.24093700	2.80711900
C	0	-10.47023500	2.35252700	-3.04012200
H	0	-10.20396900	0.73877600	-1.66005100
C	0	-9.72220700	4.52403500	-2.29779400
H	0	-8.85970000	4.59333800	-0.32806400
H	0	-9.07108200	0.77564800	3.16485900
H	0	-9.65995700	2.42066100	3.33326800
C	0	-5.44035600	1.86923300	1.29255700
H	0	-6.20374500	1.01189300	3.07016400
H	0	-6.98318600	0.43457900	1.63296200
C	0	-10.29942900	3.71264800	-3.27089200
H	0	-10.91221500	1.71859000	-3.79984400
H	0	-9.59451100	5.58533900	-2.47949300
O	0	-5.81921400	1.90661400	-0.11285900
H	0	-4.62031100	1.15986500	1.39160800
C	0	-4.95373500	3.25785800	1.66337200
H	0	-10.61836000	4.14432900	-4.21380500
C	0	-5.15681600	1.18624500	-0.99505800
H	0	-5.76298200	3.98359500	1.52702600
H	0	-4.17196200	3.53804200	0.95323900
O	0	-4.23599900	0.42982400	-0.70648900
C	0	-5.59557500	1.38960100	-2.38229800
C	0	-5.16020400	0.48318200	-3.34984400
C	0	-6.43470300	2.44782800	-2.73071600
C	0	-5.57184000	0.63540900	-4.66358200
H	0	-4.53133100	-0.34878600	-3.05500700
C	0	-6.83303900	2.60023100	-4.04901500
H	0	-6.76964000	3.14031600	-1.96947400
C	0	-6.40502900	1.69330800	-5.01319800

H	0	-5.24970000	-0.07683300	-5.41454500
H	0	-7.48465100	3.42227400	-4.32163700
H	0	-6.72592600	1.80893800	-6.04317100
Cl	0	-1.92170700	0.62508800	1.40152000
Ti	0	0.01964900	0.80893600	-0.09336900
Cl	0	1.92686400	0.69785600	-1.55958800
C	0	1.03323700	2.04403600	1.43538200
C	0	-0.70347400	2.54931000	-1.08820500
C	0	0.42536500	3.11405800	0.79093700
H	0	2.09651300	1.86454600	1.30301100
H	0	0.57061900	1.62258100	2.32196300
C	0	-1.94094300	3.38923900	-0.90280200
H	0	-1.02339400	1.58493300	-1.57212100
H	0	0.01236000	2.99187400	-1.78329600
C	0	1.23498100	4.14808200	0.06901800
H	0	-0.53336000	3.44092700	1.18437700
H	0	-2.48292900	3.51278300	-1.84733400
H	0	-2.61668600	2.91909000	-0.18647600
H	0	-1.69523700	4.39296300	-0.54172400
H	0	0.63022100	4.79414900	-0.56907800
H	0	1.72321000	4.77977400	0.81918700
H	0	2.01171000	3.67928800	-0.53755600
C	0	-4.39490600	3.30917900	3.07851300
H	0	-3.98715400	4.29883300	3.29705300
H	0	-5.15804100	3.09575700	3.83267500
H	0	-3.59141700	2.57734500	3.20191100
C	0	4.32375800	3.79539500	-2.40989600
H	0	3.89198100	4.79908800	-2.41334700
H	0	5.07074900	3.75844600	-3.20814900
H	0	3.53258300	3.08410900	-2.66507400
C	0	-8.22937400	1.84738300	4.83609600
H	0	-7.93226000	2.86346000	5.11528500
H	0	-8.99605400	1.51924100	5.54070000
H	0	-7.36495500	1.19187700	4.97233700
C	0	8.15959600	2.67105700	-4.51244500
H	0	7.83920200	3.71372300	-4.60680800
H	0	8.91929900	2.48662800	-5.27454200
H	0	7.30508800	2.03110400	-4.74819800

$TiCl_2Et$-$MgCl_2$-2*RS*-HDDB-H_2-TS

0 2

Mg	-1	-12.53400700	-2.85363300	0.78121600
Mg	-1	-9.39668800	-1.01626300	0.62757900
Mg	-1	-6.25133400	-2.81502000	0.32115200
Mg	-1	-3.11552800	-0.98033300	0.16585800
Mg	-1	0.03216900	-2.77510200	-0.14215000

Mg	-1	3.16559400	-0.93118900	-0.29537900
Mg	-1	6.31489800	-2.72321100	-0.60170600
Mg	-1	9.44668800	-0.88204200	-0.75640300
Mg	-1	12.59449300	-2.67675400	-1.06452500
Cl	0	-14.34128900	-4.09467400	0.92683200
Cl	0	-11.26731200	-1.44982400	2.26901600
Cl	0	-8.23673600	-3.15600100	1.52948900
Cl	0	-4.94833200	-1.35706400	1.79930300
Cl	0	-1.82568300	-3.09976900	1.14278500
Cl	0	1.18765500	-1.06689100	1.26739100
Cl	0	4.48826400	-2.94383900	0.85685700
Cl	0	7.56491900	-1.01578300	0.73423100
Cl	0	11.14029200	-2.07887600	0.72245600
Cl	0	-11.07586900	-2.10121400	-0.94251700
Cl	0	-7.49031000	-0.96276000	-0.82495600
Cl	0	-4.43723000	-2.84037900	-1.17750700
Cl	0	-1.15184400	-0.96577300	-1.39421700
Cl	0	1.89041600	-2.95234800	-1.45595900
Cl	0	5.02238600	-1.10278400	-1.91876100
Cl	0	8.29845600	-2.93532400	-1.84918600
Cl	0	11.33010600	-1.14605400	-2.41598300
Cl	0	14.40056400	-3.90333300	-1.31291800
C	0	3.76872500	3.53940600	-0.49153200
C	0	4.90656200	2.56073100	-0.73215300
H	0	3.00058500	3.01650500	0.08536200
H	0	4.15145200	4.35332500	0.13532900
C	0	6.02341200	3.13939800	-1.57902200
H	0	4.53629500	1.64339300	-1.19361000
C	0	7.30509200	2.32636200	-1.62672700
H	0	5.63512400	3.22860000	-2.59630400
H	0	6.27552500	4.15229000	-1.24568500
H	0	7.10644500	1.27018700	-1.41815100
C	0	8.05408500	2.47297000	-2.94188900
H	0	8.07944200	3.53248700	-3.22211300
H	0	9.08862000	2.15162200	-2.80371600
O	0	5.47322400	2.21222400	0.55656300
O	0	8.10909600	2.83340100	-0.51356900
C	0	4.97651400	1.19567000	1.23189900
O	0	4.05877500	0.49961200	0.80747100
C	0	9.09371500	2.10473200	-0.06469000
O	0	9.46454400	1.07603600	-0.63110000
C	0	5.61588100	0.97944500	2.53293900
C	0	5.22525700	-0.12228300	3.29381700
C	0	6.64257700	1.81568000	2.97450100
C	0	5.87105500	-0.38941800	4.48972300
H	0	4.44462100	-0.77648900	2.92535400

C	0	7.28729000	1.54049200	4.16783200
H	0	6.93834700	2.66330300	2.37093500
C	0	6.90303600	0.43628900	4.92311100
H	0	5.58047600	-1.25306700	5.07661500
H	0	8.09788800	2.17914100	4.50099600
H	0	7.41566000	0.21423600	5.85314200
C	0	9.71376500	2.60025400	1.17129300
C	0	10.62118300	1.77776000	1.84009900
C	0	9.37029600	3.84727200	1.69906200
C	0	11.17472500	2.19903800	3.03884500
H	0	10.87811500	0.80725400	1.43170000
C	0	9.93566900	4.26645100	2.89208500
H	0	8.66382600	4.47482900	1.16941800
C	0	10.83468600	3.44129900	3.56302600
H	0	11.87036700	1.55503500	3.56428000
H	0	9.67666600	5.23606000	3.30227200
H	0	11.27260200	3.77029100	4.49949400
C	0	-3.87990400	3.39773400	1.11783100
C	0	-4.96964500	2.34097900	1.15986300
H	0	-3.08942500	3.02700500	0.46112400
H	0	-4.29947900	4.29839700	0.65392900
C	0	-6.11596000	2.71415700	2.08195000
H	0	-4.56289300	1.37302200	1.45646300
C	0	-7.39646400	1.91837100	1.90986100
H	0	-5.75696200	2.57233500	3.10419000
H	0	-6.36481800	3.77619300	1.97797900
H	0	-7.18460200	0.92981400	1.48969500
C	0	-8.19299800	1.79124100	3.19967300
H	0	-8.19585000	2.75939200	3.71354800
H	0	-9.23236000	1.55183100	2.96439300
O	0	-5.50691400	2.20100400	-0.18165500
O	0	-8.15467800	2.65505000	0.89642600
C	0	-4.97804400	1.32927700	-1.01513100
O	0	-4.03813300	0.59730500	-0.71490700
C	0	-9.13640800	2.05244000	0.28355400
O	0	-9.54119700	0.93829500	0.61235400
C	0	-5.60606700	1.32377200	-2.34021600
C	0	-5.20852000	0.35652900	-3.26350100
C	0	-6.62655600	2.22319200	-2.65319300
C	0	-5.84036000	0.28774500	-4.49429100
H	0	-4.43548700	-0.35345900	-2.99477400
C	0	-7.25603200	2.14732200	-3.88358300
H	0	-6.92874500	2.96329200	-1.92439000
C	0	-6.86421500	1.17778300	-4.80218500
H	0	-5.54588900	-0.47261700	-5.20837200
H	0	-8.06048400	2.83527600	-4.11942400

H	0	-7.36496800	1.11068700	-5.76225400
C	0	-9.71667600	2.79719000	-0.84144400
C	0	-10.62307200	2.13879500	-1.67363700
C	0	-9.33872500	4.11468000	-1.10926300
C	0	-11.14310000	2.79713200	-2.77686100
H	0	-10.90038800	1.11287000	-1.46000000
C	0	-9.86906300	4.76905900	-2.20915400
H	0	-8.63370700	4.61185800	-0.45395700
C	0	-10.76822100	4.10969200	-3.04340800
H	0	-11.84005100	2.28465500	-3.42990300
H	0	-9.58366400	5.79376100	-2.41886300
H	0	-11.17953700	4.62390000	-3.90559800
Cl	0	1.86237100	0.78302500	-1.53628400
Cl	0	-1.81890500	0.65229300	1.52689600
Ti	0	0.05915700	0.82840200	0.06081600
C	0	-0.17920200	3.04145500	-0.08888100
H	0	1.10539900	1.57297500	1.29751900
H	0	0.66391700	2.30662600	0.81079900
C	0	-0.92787700	2.60186900	-1.32707800
H	0	-0.83383500	3.48160700	0.66383100
H	0	0.62275700	3.73467900	-0.34009900
H	0	-1.27004300	1.53440200	-1.34738800
H	0	-1.88363900	3.11893100	-1.43779800
H	0	-0.33845800	2.71850800	-2.23577700
C	0	-3.29488000	3.72469700	2.48441600
H	0	-2.45735400	4.41975200	2.38351600
H	0	-4.02921000	4.19941500	3.14008100
H	0	-2.92024400	2.82428800	2.97695200
C	0	-7.61909700	0.70093500	4.09587200
H	0	-6.57179300	0.88306800	4.35309800
H	0	-8.18438800	0.63491500	5.02730300
H	0	-7.66313200	-0.27851400	3.60974100
C	0	7.41246500	1.63385600	-4.03962000
H	0	6.37405300	1.92077000	-4.22878800
H	0	7.96168800	1.74551200	-4.97645700
H	0	7.41102700	0.57231800	-3.77544900
C	0	3.16484200	4.09631800	-1.77314200
H	0	2.82607400	3.29135900	-2.43000700
H	0	2.30237300	4.72878400	-1.54646200
H	0	3.87632600	4.71639300	-2.32438000

$TiCl_2Et$-$MgCl_2$-2*SS*-HDDB-H_2-TS

0 2

Mg	-1	-12.55296000	-2.79767100	0.79299600
Mg	-1	-9.41300800	-0.97090500	0.63021500
Mg	-1	-6.26972600	-2.77879000	0.35902100

Mg	-1	-3.12968800	-0.95208800	0.19644300
Mg	-1	0.01351400	-2.76000900	-0.07516400
Mg	-1	3.15364500	-0.93334300	-0.23759200
Mg	-1	6.29682400	-2.74107500	-0.50895100
Mg	-1	9.43674100	-0.91432500	-0.67168600
Mg	-1	12.58007700	-2.72225200	-0.94294200
Cl	0	-14.35047000	-4.04857900	0.96378800
Cl	0	-11.33519900	-1.30042200	2.22611900
Cl	0	-8.26139800	-3.04929900	1.60451400
Cl	0	-4.92920600	-1.39236900	1.84560700
Cl	0	-1.84195500	-3.04065500	1.21486100
Cl	0	1.16127000	-1.01500600	1.32272100
Cl	0	4.47050000	-2.94010800	0.96255900
Cl	0	7.56129300	-1.07220000	0.84426500
Cl	0	11.08323600	-2.16727200	0.81943200
Cl	0	-11.05931200	-2.12569300	-0.93397700
Cl	0	-7.53248200	-1.02019500	-0.88434000
Cl	0	-4.44721900	-2.86671700	-1.12994400
Cl	0	-1.13603900	-0.92746500	-1.33880800
Cl	0	1.86863800	-2.94664000	-1.38063600
Cl	0	4.95226500	-1.26789000	-1.91071800
Cl	0	8.28583800	-2.92529100	-1.77796100
Cl	0	11.36249400	-1.13756700	-2.28007000
Cl	0	14.38034600	-3.95554200	-1.19649100
O	0	9.29723000	1.06069000	-0.67523000
C	0	9.05185200	2.11983400	-0.09686000
O	0	8.31592900	3.05937500	-0.62676800
C	0	9.56570100	2.45163500	1.24145700
C	0	7.65618600	2.88193700	-1.92078000
C	0	10.20208500	1.46500300	1.99462100
C	0	9.40526200	3.73738600	1.76610900
C	0	8.65509300	2.64914700	-3.04696000
C	0	6.55219500	1.84412100	-1.84045600
H	0	7.21882000	3.87382800	-2.05900300
C	0	10.66772700	1.75905000	3.26612900
H	0	10.32764300	0.46311100	1.60359200
C	0	9.88271600	4.02879500	3.03321700
H	0	8.91045400	4.49638900	1.17306000
H	0	8.98753300	1.60951900	-3.05471000
H	0	9.53851600	3.26430000	-2.84595500
C	0	5.37136100	2.13563700	-0.92915500
H	0	6.14671400	1.72622000	-2.84957700
H	0	6.95271800	0.86415400	-1.57728800
C	0	10.51204700	3.03939500	3.78398700
H	0	11.15111000	0.98485900	3.85040600
H	0	9.76684300	5.02823000	3.43741700

O	0	5.78561300	1.94329600	0.45105600
H	0	4.60597600	1.39497500	-1.15120000
C	0	4.76115800	3.52172300	-1.02487000
H	0	10.88348400	3.26926000	4.77710900
C	0	5.12406200	1.10166500	1.21750100
H	0	5.49189700	4.27154800	-0.70140300
H	0	3.93558900	3.56143600	-0.30644900
O	0	4.17669600	0.42925300	0.82218700
C	0	5.60101400	1.04850500	2.60426300
C	0	5.12858000	0.02475800	3.42598500
C	0	6.52350400	1.97590700	3.08864200
C	0	5.58857300	-0.07291800	4.72887100
H	0	4.43085100	-0.70057700	3.02499200
C	0	6.97292300	1.87688400	4.39490100
H	0	6.88422100	2.76268100	2.43937900
C	0	6.50869000	0.85132100	5.21267100
H	0	5.23599100	-0.87644700	5.36525300
H	0	7.69231600	2.59482400	4.77146300
H	0	6.86905300	0.76956700	6.23279600
O	0	-9.28528200	1.00099200	0.76165500
C	0	-9.04229800	2.09674200	0.25543200
O	0	-8.30537100	2.99940400	0.84499300
C	0	-9.56009900	2.51662900	-1.05643300
C	0	-7.64633400	2.73776900	2.12521200
C	0	-10.19760600	1.58131700	-1.87163800
C	0	-9.40314600	3.83477500	-1.49462600
C	0	-8.64608300	2.43217700	3.23309400
C	0	-6.54311600	1.70642100	1.97855300
H	0	-7.20877800	3.71836900	2.32814400
C	0	-10.66792900	1.95886200	-3.11918400
H	0	-10.32103700	0.55560500	-1.54722900
C	0	-9.88491100	4.20925400	-2.73810300
H	0	-8.90832400	4.55361600	-0.85345700
H	0	-8.97894300	1.39440500	3.17248600
H	0	-9.52908300	3.05959400	3.07215500
C	0	-5.35905900	2.05548100	1.09231900
H	0	-6.13996900	1.52105500	2.97839200
H	0	-6.94405000	0.74674100	1.64976900
C	0	-10.51553900	3.27113200	-3.55112200
H	0	-11.15285600	1.22480500	-3.75198400
H	0	-9.77219700	5.23371400	-3.07492600
O	0	-5.77003700	1.95427000	-0.29940400
H	0	-4.59611600	1.30011200	1.26751800
C	0	-4.74841600	3.43166300	1.28164700
H	0	-10.89105000	3.56624600	-4.52528500
C	0	-5.09858600	1.17779700	-1.12250600

H	0	-5.47696300	4.20165200	1.00385000
H	0	-3.91855400	3.51846800	0.57185000
O	0	-4.13597600	0.49723000	-0.77787400
C	0	-5.57803100	1.21302300	-2.50903000
C	0	-5.09304300	0.25499800	-3.40009500
C	0	-6.51572600	2.15765200	-2.92678800
C	0	-5.55488500	0.24082700	-4.70596600
H	0	-4.38571000	-0.48774600	-3.05091200
C	0	-6.96677300	2.14251000	-4.23609500
H	0	-6.88821900	2.89089200	-2.22373500
C	0	-6.48941800	1.18287400	-5.12342300
H	0	-5.19349800	-0.51252300	-5.39648400
H	0	-7.69849000	2.87326500	-4.56064300
H	0	-6.85161200	1.16592500	-6.14600800
Cl	0	-1.91832300	0.80404100	1.48400800
Cl	0	1.91598500	0.89158200	-1.41489900
Ti	0	0.04880300	0.89443800	0.11248700
H	0	1.04063500	1.63479400	1.39705800
C	0	-0.95173800	2.66085700	-1.26456100
H	0	0.63020200	2.36873300	0.89208700
C	0	-0.15399500	3.10920100	-0.06159400
H	0	-1.14645300	1.55831700	-1.36916100
H	0	-1.97202400	3.04735900	-1.25390700
H	0	-0.46215900	2.90825000	-2.20605000
H	0	-0.77252800	3.61295400	0.68137000
H	0	0.68685900	3.73994100	-0.35031400
C	0	-4.22836500	3.64817100	2.69575200
H	0	-3.72223300	4.61292200	2.77629100
H	0	-5.03317500	3.63864600	3.43644500
H	0	-3.51180100	2.86845000	2.96787200
C	0	-8.06392100	2.71933000	4.61082000
H	0	-7.73537900	3.75999000	4.69832500
H	0	-8.81493200	2.54194700	5.38317100
H	0	-7.21041300	2.07474900	4.83776300
C	0	4.23563400	3.83016200	-2.41980600
H	0	3.51923700	3.06835500	-2.73944200
H	0	3.72808200	4.79754100	-2.43490100
H	0	5.03703300	3.87034600	-3.16328000
C	0	8.07211600	3.02622900	-4.40246800
H	0	7.74382900	4.07050200	-4.42111700
H	0	8.82242100	2.89970800	-5.18547000
H	0	7.21813000	2.39818000	-4.67032800
